# Structure-switching M_3_L_2_ Ir(iii) coordination cages with photo-isomerising azo-aromatic linkers†
†Data accessibility: data are available at https://doi.org/10.5518/438
‡
‡Electronic supplementary information (ESI) available: Full experimental details, details of crystallography, spectra. CCDC 1839930, 1839931, 1859217 and 1859218. For ESI and crystallographic data in CIF or other electronic format see DOI: 10.1039/c8sc03499k


**DOI:** 10.1039/c8sc03499k

**Published:** 2018-09-06

**Authors:** Samuel Oldknow, Diego Rota Martir, Victoria E. Pritchard, Mark A. Blitz, Colin W. G. Fishwick, Eli Zysman-Colman, Michaele J. Hardie

**Affiliations:** a School of Chemistry , University of Leeds , Woodhouse Lane , Leeds LS2 9JT , UK . Email: m.j.hardie@leeds.ac.uk; b Organic Semiconductor Centre , EaStCHEM School of Chemistry , University of St Andrews , St Andrews , Fife KY16 9ST , UK

## Abstract

Cyclotriguaiacylene has been functionalised with 3- or 4-pyridyl-azo-phenyl groups to form a series of molecular hosts with three azobenzene-type groups that exhibit reversible photo-isomerisation. Reaction of the host molecules with [Ir(C^N)_2_(NCMe)_2_]^+^ where C^N is the cyclometallating 2-phenylpyridinato, 2-(4-methylphenyl)pyridinato or 2-(4,5,6-trifluorophenyl)pyridinato results in the self-assembly of a family of five different [{Ir(C^N)_2_}_3_(L)_2_]^3+^ coordination cages. Photo-irradiation of each of the cages with a high energy laser results in *E* → *Z* photo-isomerisation of the pyridyl-azo-phenyl groups with up to 40% of groups isomerising. Isomerisation can be reversed by exposure to blue light. Thus, the cages show reversible structure-switching while maintaining their compositional integrity. This represents the largest photo-induced structural change yet reported for a structurally-integral component of a coordination cage. Energy minimised molecular models indicate a switched cage has a smaller internal space than the initial all-*E* isomer. The [Ir(C^N)_2_(NCMe)_2_]^+^ cages are weakly emissive, each with a deep blue luminescence at *ca.* 450 nm.

## Abstract 2

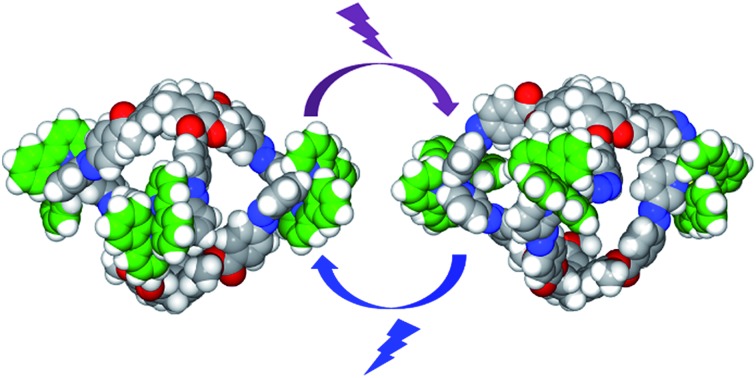
Deep-blue luminescent [{Ir(C^N)_2_}_3_(L)_2_]^3+^ coordination cages with structurally integral pyridyl-azo-phenyl groups can be reversibly photo-isomerised with no compositional change.

## Introduction

Coordination cages, also referred to as metallosupramolecular cages, metallo-cages or as metal–organic polyhedra, are 3-D assemblies of metal cations and bridging ligands with well-defined discrete nano-sized structures often related to Platonic or Archimedean solids.^1^ As container molecules, they feature internal cavities in which other molecules or ions may be bound. Exploitation of their binding properties leads to a raft of potential applications as sensors, catalysts and as nano-scale reaction vessels where confinement of reagents affects properties.^1^

There has been recent interest in stimuli-responsive behavior of coordination cages, thus moving away from static to more complicated dynamic systems.^2^ Most examples are chemo-responsive, and often involve a degree of compositional change.^2^ We were interested, however, in inducing significant and reversible structural – but not compositional – change in a coordination cage. Such cages would show controllable dynamic size and shape behavior of both their external morphology and internal cavity space, the latter significant for control of host–guest properties and concomitant functionality. Photo-irradiation is an attractively clean and efficient mechanism for structural changes. Photo-isomerisable coordination cages are rare. Clever *et al.* have reported the only examples of 3D coordination cage species where bridging ligands inherent to the cage structure have been photo-isomerised.^3^,^4^ These are a [Pd_2_L_4_]^4+^ cage with a bis(3-pyridyl)dithienylethene (DTE) ligand that displays reversible ring-open ↔ ring-closed photo-switching,^3^ and a [Pd_3_L_6_]^6+^ ↔ [Pd_24_L_48_]^48+^ cage transformation that occurs between ring-open and ring-closed ligand isomers of the bridging L which is a bis(4-pyridyl)DTE ligand.^4^
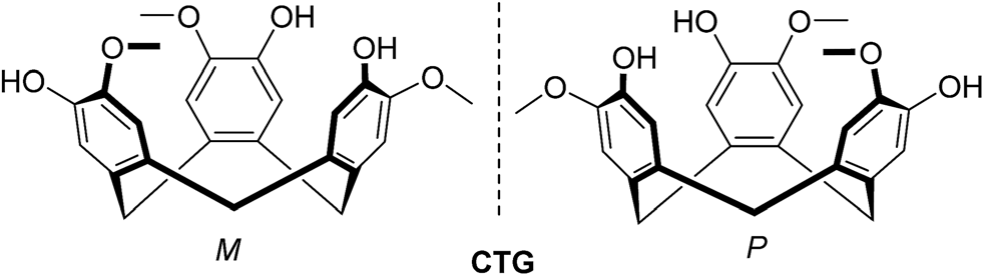


One of the most widely employed photo-switching motifs is azobenzene (AZB) which exhibits reversible and robust *E* → *Z* photo-isomerisation.^5^ The AZB motif has been used as a photosensitive component in a number of different types of responsive systems including liquid crystals and polymers,^6^ molecular machines,^7^ metal complexes,^8^ and host–guest systems.^9^ The use of AZB in coordination cage chemistry is limited, and photo-switching has only been shown where the AZB unit is an endohedral or exohedral decoration that is not part of the structural integrity of the cage.^10^,^11^ Fujita *et al.* have reported a Pd_12_L_24_ cage where the L-ligand contains pendant AZB units that occupy the inside of the cage.^10^ Photo-switching of the AZB controls the internal cage hydrophobicity. Photo-responsive 2-D metallacyclic assemblies with bridging AZB-type ligands have been reported,^12^,^13^ although photo-switching can induce both structural and compositional change.^12^ Hydrogen-bonded capsule or cage assemblies have been reported with photo-responsive, though structurally peripheral, AZB groups for both hexameric calix[4]resorcinarenes cages^14^ and capsule assemblies of urea-functionalised calix[4]arenes.^15^ Their photo-responsive properties can be used to regulate the stability of the assembly or host–guest behaviour.

In this work, we report a series of coordination cages of type [{Ir(C^N)_2_}_3_(L)_2_]^3+^ where C^N is a cyclometallating ligand and L is a tripodal cyclotriguaiacylene (CTG) derivative functionalised with pyridyl-azo-phenyl groups. CTG is a chiral host molecule with *M* and *P* isomers and appropriate functionalisation gives classes of ligands (L) that have proved ideal for assembly of cages including M_3_L_2_ (ref. 16–18) and M_6_L_8_ (ref. 19 and 20) species. An azobenzene-appended CTG has been reported as a chemosensor for Hg(i) but its photo-responsive properties were not investigated.^21^ Other classes of host molecules have also been decorated with AZB-type groups,^14^,^15^,^22^ or linked together into photo-responsive dimers *via* an AZB.^23^ Each [{Ir(C^N)_2_}_3_(L)_2_]^3+^ cage features six azobenzene-type linkages and photo-isomerises. These are the first examples of a coordination cage with AZB-type linkers that display reversible photo-switching, as well as being only the second example of any type of coordination cage where structural building units can photo-isomerise without compositional change. These cages display unusual luminescence properties for cationic Ir(iii) complexes, with deep blue phosphorescence, albeit at low quantum yields (*Φ*_PL_). Despite its inherent luminescent properties there are very few known examples of homonuclear,^17^,^24^ or mixed-metal^25^ coordination cages employing a Ir(C^N)_2_ motif.

## Results and discussion

### Photo-switchable host-type ligands

**Scheme 1:**
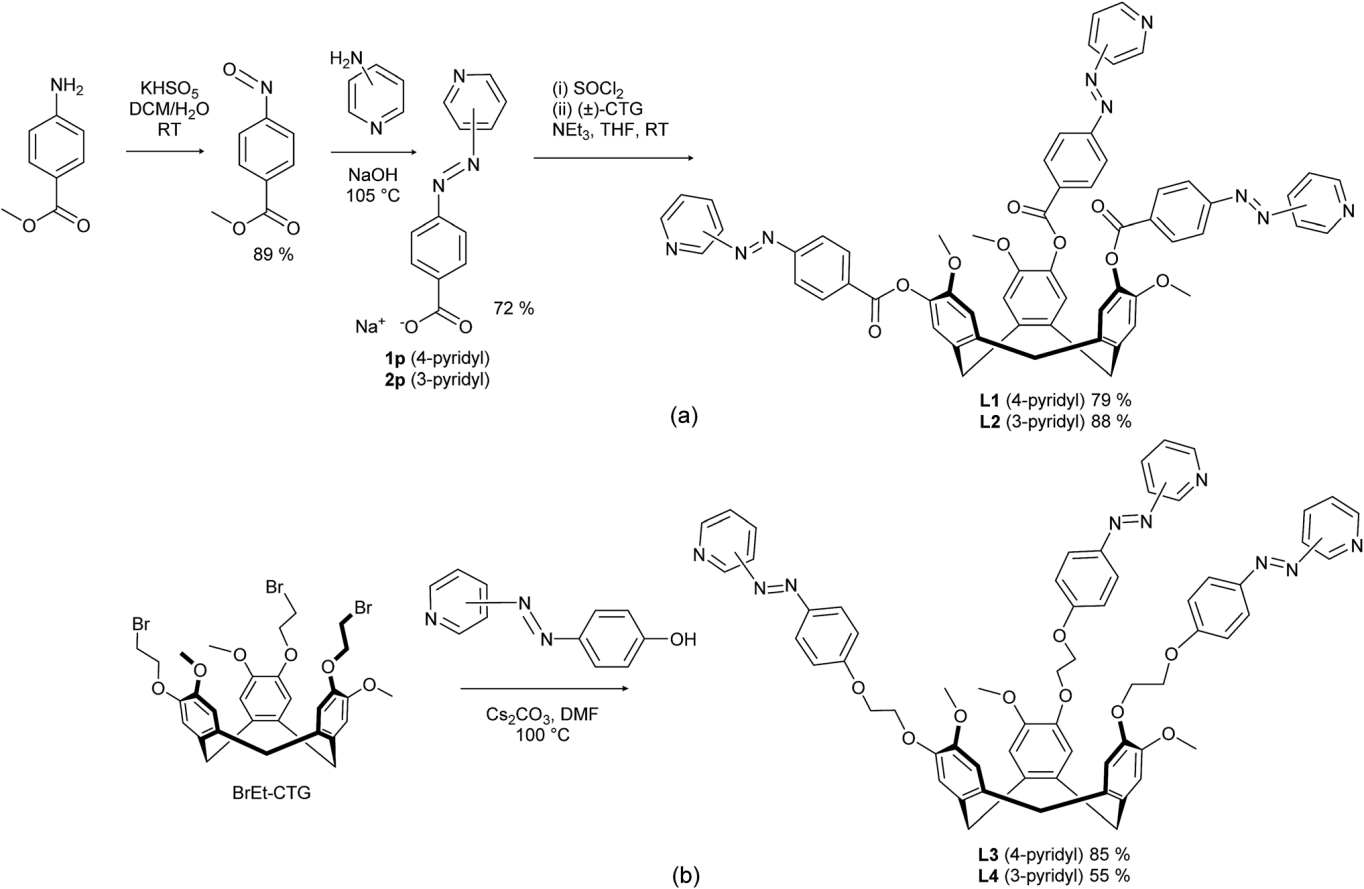
Synthesis of (a) ester-linked ligands **L1** and **L2**; (b) glycol-linked ligands **L3** and **L4**.

The ligands (±)-2,7,12-trimethoxy-3,8,13-tris(4,3′-pyridyl-azophenylcarboxy)-10,15-dihydro-5*H*-tribenzo[*a*,*d*,*g*]cyclononene **L1** and (±)-2,7,12-trimethoxy-3,8,13-tris(4,3′-pyridyl-azophenylcarboxy)-10,15-dihydro-5*H*-tribenzo[*a*,*d*,*g*]cyclononene **L2** were synthesised in good yields by reaction of (±)-CTG with the acid chloride of the appropriate sodium [2-(pyridyl)diazenyl]-benzoate (see ESI‡ for crystal structures)¶
¶CCDC 1839930 (**2p**·5H_2_O), 1839931 (**L2**), 1859217 (**1p**·4H_2_O), 1859218 (**L3**)·2(CH_3_NO_2_), the latter accessed by reaction of 4-nitrosomethylbenzoate with 3- or 4-aminopyridine, Scheme 1a. Reaction of (±)-2,7,12-trimethoxy-3,8,13-tris(2-bromoethoxy)-10,15-dihydro-5*H*-tribenzo[*a*,*d*,*g*]cyclononene (BrEt-CTG)^26^ with the appropriate 3- or 4-pyridylazophenol^27^ according to Scheme 1b gives the tripodal ligands (±)-2,7,12-trimethoxy-3,8,13-tris(2-(4-pyridylazo)ethoxy)-10,15-dihydro-5*H*-tribenzo[*a*,*d*,*g*]cyclononene **L3** and (±)-2,7,12-trimethoxy-3,8,13-tris(2-(3-pyridylazo)ethoxy)-10,15-dihydro-5*H*-tribenzo[*a*,*d*,*g*]cyclononene **L4**. All ligands **L1–L4** were characterised by mass spectrometry (MS), NMR and UV-visible spectroscopies with spectra consistent with the proposed structure (Fig. S6–S47, ESI†). **L1** proved insufficiently soluble in non-coordinating solvents for self-assembly studies and will not be discussed further.

**Fig. 1:**
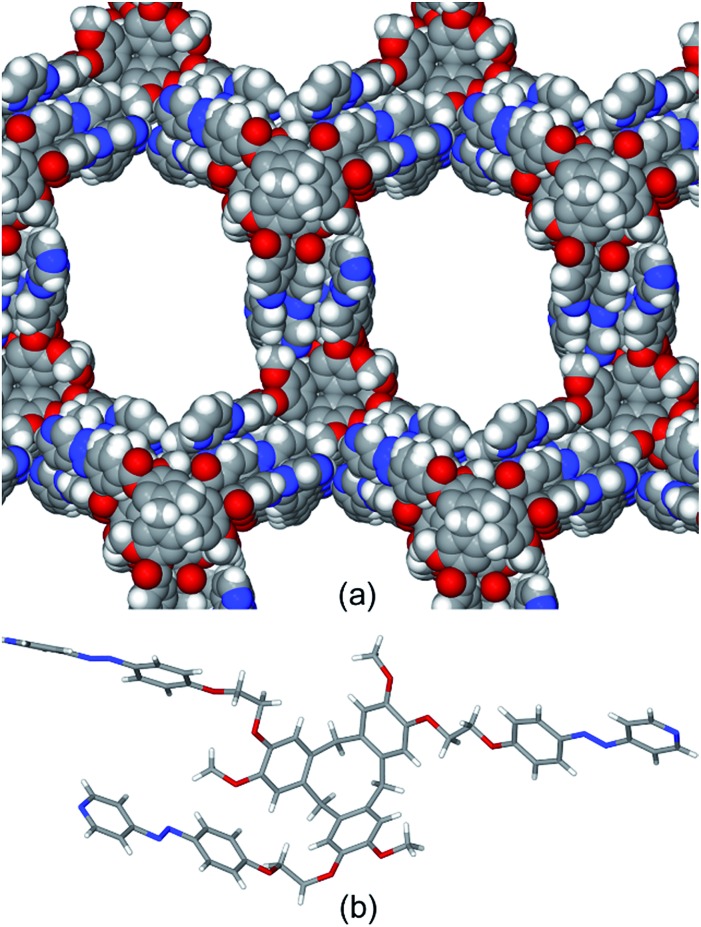
From the crystal structures of (a) **L2** (some disorder not shown, see Fig. S3†); (b) **L3**·2(MeNO_2_).

The crystal structures of **L2** and **L3** were determined.¶ There are two crystallographically distinct molecules of **L1** each of opposite enantiomer. The ligands have approximate *C*_3_-symmetry and alternating enantiomers of **L2** stack in an offset bowl-in-bowl fashion, forming columns running along the *a* axis. Adjacent columns inter-lock through π–π stacking interactions between the 3-pyridylazophenyl groups to create a crystal lattice with void channels of over 2.5 nm diameter, Fig. 1a. The solvent-accessible void space accounts for 48% of the unit cell volume, which is extremely high for a molecular crystal. **L3** crystallises as a CH_3_NO_2_ clathrate, and the host molecule has all three 4-pyridylazophenyl groups in different orientations, Fig. 1b.

**Fig. 2:**
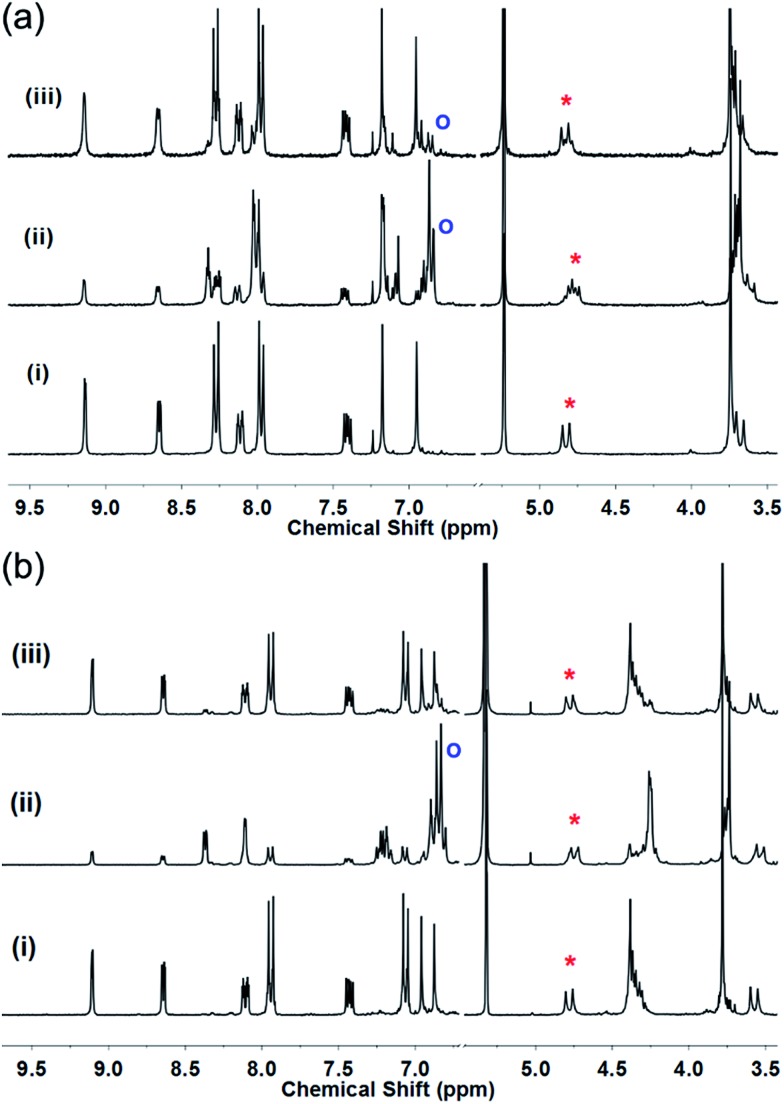
^1^H NMR spectra (300 MHz) in CD_2_Cl_2_. (a) Ligand **L2** and (b) ligand **L4** showing in each case (i) initial spectrum, (ii) after irradiation with 355 nm laser to *Z*-rich form; (iii) after irradiation with 450 nm light to return to *E* state. Red asterix indicates CTG–CH_2_– proton and blue circle indicates aromatic proton shielded in *Z*-isomer.

**Fig. 3:**
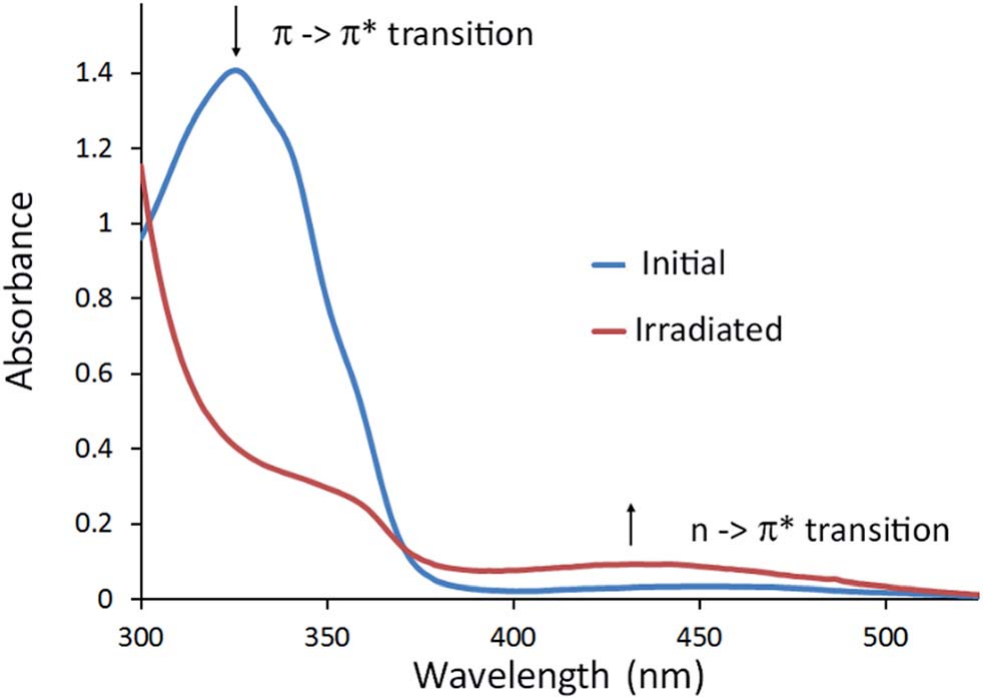
UV-visible spectra of **L2** in CHCl_3_ (30 μM), blue trace initial spectrum, red trace after irradiation with 355 nm laser to photo-stationary state.

Photo-isomerisation of each of **L2–L4** can result in four possible isomers: (*EEE*), (*EEZ*), (*EZZ*), or (*ZZZ*). Changes to the ^1^H NMR spectrum of **L2** in CD_2_Cl_2_ on irradiation with a 355 nm Nd:YAG laser indicate a mixture of isomers is formed as the diastereotopic bridging methylene CTG protons become a complex multiplet, Fig. 2a. A new doublet appears at 6.9 ppm due to an increase in shielding of the aromatic protons *ortho* to the azo groups on isomerisation to the *Z* isomer. Relative integrations gives approximately 76% conversion to *Z* isomers, corroborated by UV-visible spectroscopy (Fig. S81†) where the π → π* transition that typically occurs between 300–350 nm for *E*-AZB-type groups reduces in intensity on isomerisation to the *Z* isomer with a concomitant small increase in the n → π* transition, Fig. 3. Azobenzene itself shows 80% *Z*-isomer at the photo-stationary state on irradiation with 313 nm light,^5^ hence the presence of three photo-isomerisable units on a single scaffold does not significantly inhibit the isomerisation. The reverse *Z* → *E* isomerisation is induced with 450 nm light, albeit not with 100% conversion (Table S3, Fig S81†). Solutions of *E* → *Z* switched **L2** show significant amounts of *Z* isomer present, even after standing for 48 hours in the dark (Fig. S84†), indicating a *Z*-rich photo-stationary state of unusually high stability. The other ligands can also be photo-switched (Table S3, Fig. S82–S84†) though interestingly NMR studies of switched glycol-linked **L4** (73% conversion) did not show significant de-symmetrisation, Fig. 2b. This implies fewer species are present in solution compared to the photo-stationary state of **L2**, and that there is a predominant species. Irradiated **L4** shows near complete conversion back to the all-*E* isomer after 24 hours standing in the dark (Fig. S85†). This is due to the more electron-donating alkoxy substituent of **L4** leads to a decreased energy barrier for the thermal isomerisation *via* the donation of electrons into the π* orbital.^5^

### Photo-switchable [{Ir(C^N)_2_}_3_(L)_2_]^3+^ coordination cages

**Scheme 2:**
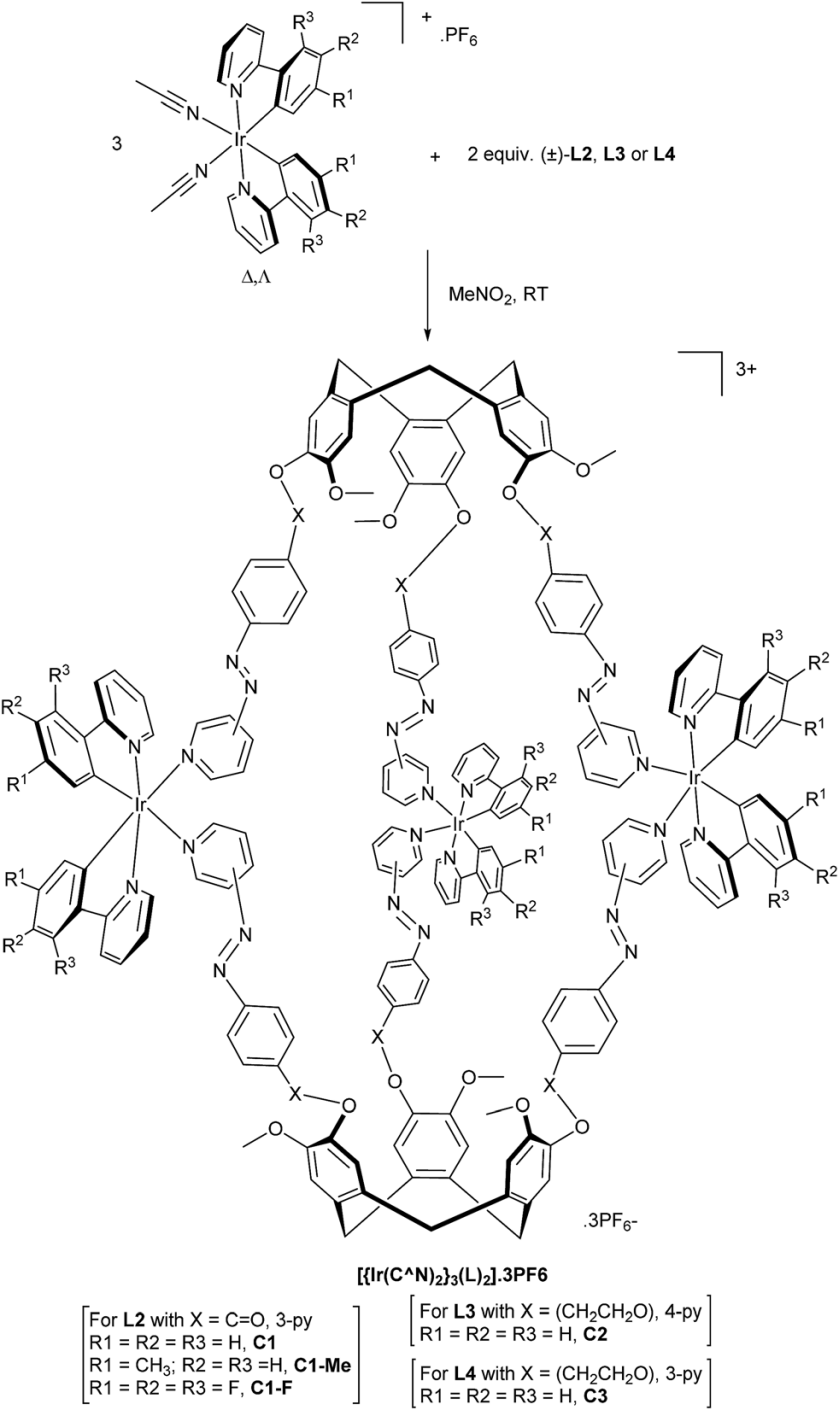
Synthesis of [{Ir(C^N)_2_}_3_(L)_2_]·3PF_6_ coordination cages.

**Fig. 4:**
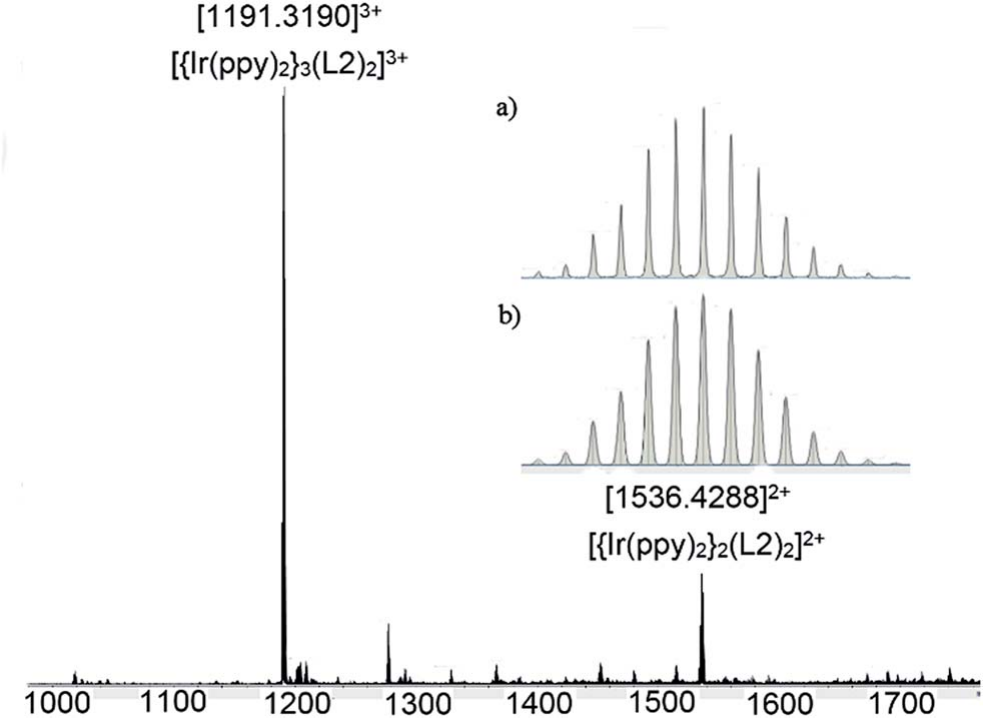
ESI-MS of [{Ir(ppy)_2_}_3_(**L2**)_2_]·3PF_6_**C1** after 24 h equilibration. Inset shows (a) experimental and (b) calculated isotope pattern for [{Ir(ppy)_2_}_3_(**L2**)_2_]^3+^.

A series of coordination cages of type [{Ir(C^N)_2_}_3_(L)_2_]·3PF_6_ were assembled in nitromethane through reaction of a 3 : 2 mixture of Δ,Λ-Ir(C^N)_2_(NCMe)_2_·PF_6_ and L-type ligand Scheme 2. Cages did not form in more coordinating solvents such as dimethylsulfoxide. The cages show similar spectroscopic behaviour, hence only [{Ir(ppy)_2_}_3_(**L2**)_2_]·3PF_6_ (**C1**, ppy = 2-phenylpyridinato) will be discussed in detail. Self-assembly of **C1** is demonstrated by ESI-MS which shows a dominant peak at *m*/*z* 1191.3190 corresponding to [{Ir(ppy)_2_}_3_(**L2**)_2_]^3+^, along with a small amount of fragmentation product, Fig. 4. This level of fragmentation is consistent with MS seen for our previously reported [{Ir(ppy)_2_}_3_(L)_2_]^3+^ cages where L are simpler 4-pyridyl-decorated CTG ligands.^17^ In that study, as here, M_2_L_2_ species seen in MS were attributed to fragmentation in the mass spectrometer as DOSY NMR showed only a single large species in solution.^17^ Despite the inertness of the d^6^ Ir(iii), the self-assembly of **C1** is largely complete after only one hour of equilibration, Fig. S49.†

**Fig. 5:**
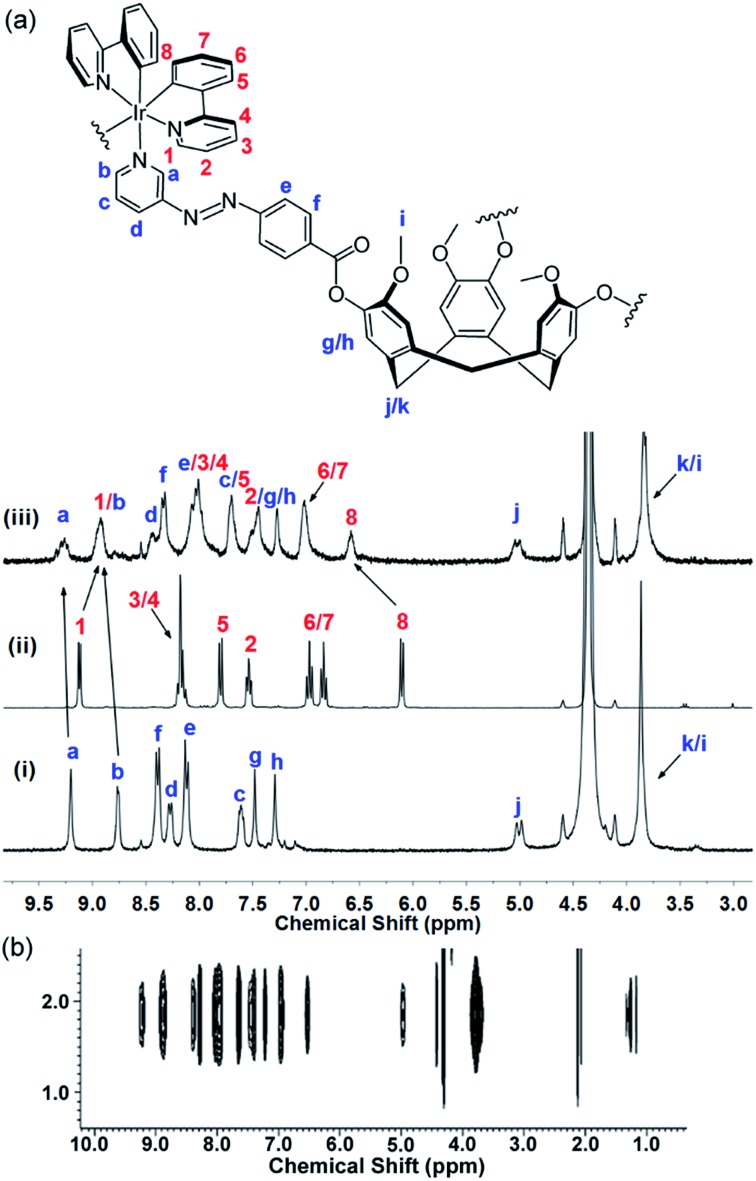
Interpreted ^1^H NMR of **C1** in CD_3_NO_2_ with numbering scheme. (a) ^1^H spectra (300 MHz) of (i) ligand **L2**; (ii) precursor [Ir(ppy)_2_(NCMe)_2_]·2PF_6_; (iii) [{Ir(ppy)_2_}_3_(**L2**)_2_]·3PF_6_**C1** after overnight equilibration. (b) Section of ^1^H DOSY spectra (600 MHz) of **C1**.

The ^1^H NMR spectrum of **C1** shows coordination-induced shifts that are indicative of complex formation and the degree of spectral broadening is typical of coordination cages, especially where different stereoisomers may be present, Fig. 5a. Significant shifts are observed for the protons closest to the iridium centre on the ppy ligands with H_1_ shifted upfield from 9.12 to 8.92 ppm and H_8_ shifted downfield from 6.10 to 6.58 ppm. The **L2***H*_a_ resonances are shifted downfield from 9.20 to 9.29 ppm. Spectra were insufficiently resolved to distinguish between ppy ligands. ^1^H ROESY NMR provided further evidence for cage formation with unambiguous through-space coupling between protons on the ppy and **L2** ligands, which will be discussed further below in the context of the modelling studies. Significant shifts are observed for the protons on the ppy ligands closest to the iridium centre and the proton *ortho* to the coordinating N on **L2**. ^1^H DOSY NMR (Fig. 5b) shows a single large species in solution with a diffusion constant of 1.86 × 10^–10^ m^2^ s^–1^ giving an approximate hydrodynamic radius of 17.32 Å. As expected, this is larger than those reported for M_3_L_2_ cages with smaller CTG-type ligands.^16^,^17^ The diffusion constant of **L2** in MeNO_2_ could not be obtained due to its poor solubility. Monitoring of the ^1^H NMR spectrum of **C1** over 9 months indicated no degradation of the cage in solution (Fig. S50†).

The UV-visible spectrum of **C1** in CH_2_Cl_2_ (Fig. S54†) is similar to that of reported for mononuclear [Ir(ppy)_2_(N)_*n*_]^+^ complexes (N = AZB-decorated pyridyl-donor ligand(s)),^28^ with ligand-centred transitions at *λ*_max_ 274 nm and 293 nm localized on the ppy ligands, and a pyridylazophenyl π → π* transition in the region of 300–390 nm, which overlaps with the spin-allowed mixed metal-to-ligand and ligand-to-ligand charge transfer transitions. The weak band at 400 nm is likely linked to the spin-forbidden n → π* pyridylazophenyl-centred transitions, which overlap with both the spin-allowed and spin-forbidden mixed charge transfer transitions.

**Fig. 6:**
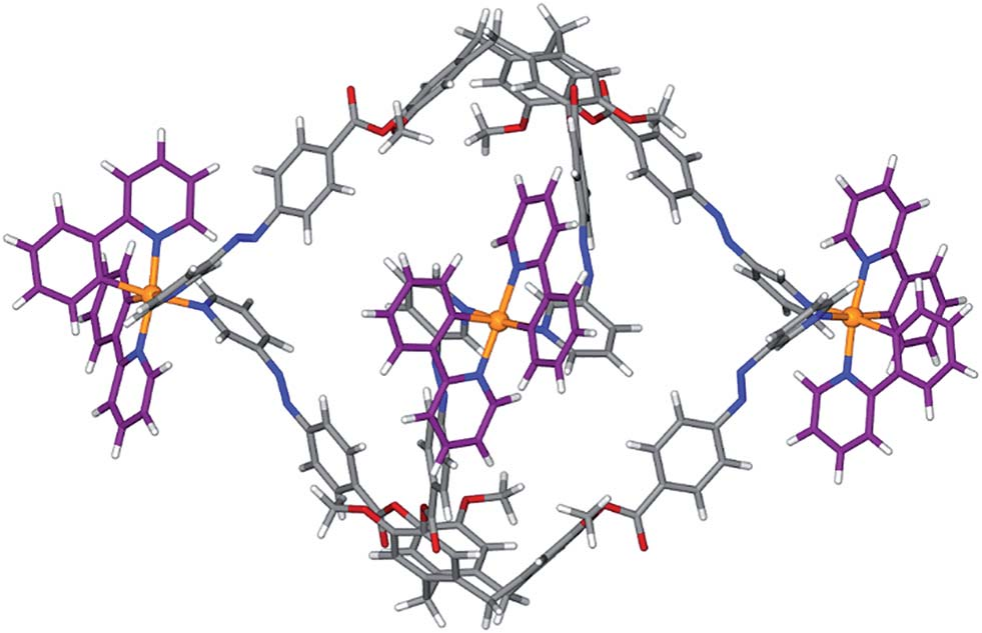
Energy-minimized structure of [{Ir(ppy)_2_}_3_(**L2**)_2_]^3+^.

Despite numerous attempts, we could not obtain crystals suitable for X-ray analysis, so molecular modelling was undertaken. The ^1^H NMR spectrum of **C1** undergoes some sharpening on standing in CD_3_NO_2_ with no concomitant changes to the ESI-MS. We have previously demonstrated that such behaviour indicates chiral self-sorting in [{Ir(ppy)_2_}_3_(L)_2_]^3+^ (ref. 17) and other cages^19^ where L = 4-pyridyl-appended CTG. Hence, the geometry of a chiral *MM*,ΔΔΔ isomer of [{Ir(ppy)_2_}_3_(**L1**)_2_]^3+^ was optimised using a MMFF force field.^29^ The structural model is an oblate trigonal prism with a triangular arrangement of Ir(iii) centres at Ir···Ir separations of 21.3, 23.6 and 25.7 Å, Fig. 6. The distance from the centres of the cage to its edges averages *ca.* 15 Å, consistent with the hydrodynamic radius of **C1** measured by DOSY NMR. There is a degree of twisting of the 3-pyridylazophenyl moieties away from planarity, with average angle between aromatic azo planes of 52°. Similar twisting has been observed in coordination polymers of *E*-azo-bis(pyridines).^30^ The energy-minimized structure is consistent with experimental observations from the 2D ROESY studies (Fig. S52†), where through-space interactions may be observed to a separation limit of approximately 5-to-6 Å. A weak ROE is observed between *H*_a_ on **L2** and H_8_ of ppy, which are separated by 5.14 Å in the model. An intense peak is observed for coupling between *H*_a_ (**L2**) and an overlapped peak of H_1_ (ppy, 2.32 Å distance to *H*_a_) and *H*_b_ (**L2**, 4.18 Å to *H*_a_). Overall, all the observed ROEs are consistent with the structure proposed by the model and full assignments are given Table S2 (ESI†).

A family of cages with pyridyl-azophenyl-derived CTG ligands can be accessed by variation of the cyclometallating ligand, or through variation of the CTG-ligand. The analogous cages [{Ir(Meppy)_2_}_3_(**L2**)_2_]·3PF_6_**C1-Me** (Meppy = 2-(4-methylphenyl)pyridinato), [{Ir(4,5,6-tFppy)_2_}_3_(**L2**)_2_]·3PF_6_**C1-F** (4,5,6-tFppy = 2-(4,5,6-trifluorophenyl)pyridinato) [{Ir(ppy)_2_}_3_(**L3**)_2_]·3PF_6_**C2** and [{Ir(ppy)_2_}_3_(**L4**)_2_]·3PF_6_**C3** all form in a similar manner to **C1** (Figs. S55–S80†).‖
‖Cage **C3** showed higher levels of fragmentation in the mass spectra than did other cages (Fig. S76†), and had a broader 1D ^1^H NMR spectrum (Fig. S77†), however the ^1^H DOSY spectrum (Fig. S79†) was definitive being very similar to that of cage **C4** (Fig. S73†) and clearly showing a single large species in solution, along with solvent. The increased conformational flexibility of **L3** and **L4** allows for cages to be formed from either 3-pyridyl or 4-pyridyl ligand donor groups. The majority of coordination cages with coordinating 3-pyridyl groups are M_2_L_4_ cages,^3^,^31^ with remarkably few reported examples of other types of cage constructs.^32^

**Fig. 7:**
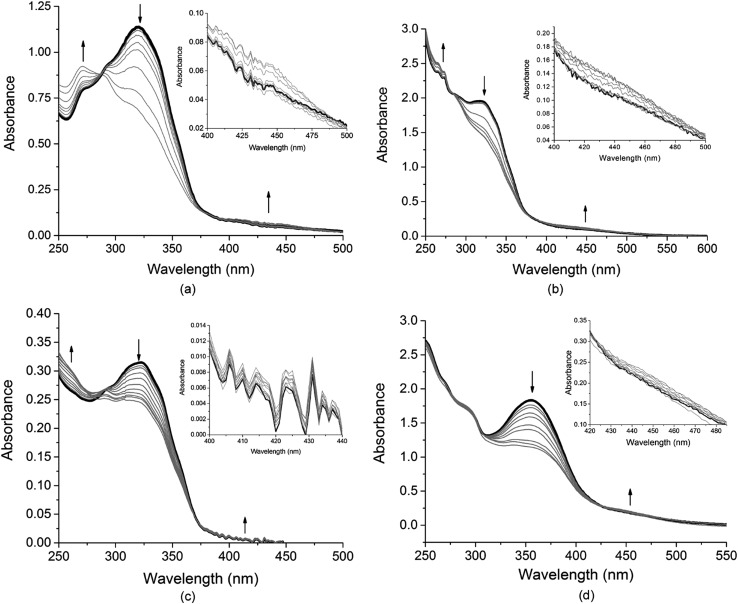
UV-visible spectra showing *E* → *Z* photo-isomerization of cages in DCM solution indicated by reduction in intensity of π → π* absorption on step-wise irradiation with 355 nm Nd:YAG laser, and insets showing expansion of n → π* transition increasing in intensity with isomerisation. Each sample was irradiated in steps to a photo-stationary state where further irradiation did not produce more spectral changes. (a) Cage **C1** with total irradiation time 255 s; (b) cage **C1-Me** with total 967 s irradiation; (c) cage **C1-F** irradiated for total 767 s; (d) cage **3** irradiated for a total of 499 s. Black line is initial spectrum, grey lines spectra on subsequent irradiations.

Photo-isomerisation experiments on all coordination cages were performed in CH_2_Cl_2_ (DCM).**
**Cages are most soluble in MeNO_2_ but this is not a suitable solvent for photo-isomerization experiments due to its high UV cut-off and possible photo-decomposition. All cage complexes show photo-induced changes to their absorption spectra indicative of *E* → *Z* isomerisation on irradiation at 355 nm, Fig. 7 and S87.† Control experiments with irradiation of the starting material [Ir(ppy)_2_(NCMe)_2_]·PF_6_ show no spectral changes (Fig. S86†). For each cage, the absorbance associated with the azoaromatic π → π* decreased in intensity while the weak band associated with n → π* transition of the *Z* isomer increased slightly.

Genuine isosbestic points are not observed, as is expected for formation of a complicated mixture of different cage isomers that are likely produced here. Cages **C1** and **C4** gave the highest degree of photo-isomerisation at 39% and 40% conversion to *Z* isomers, compared with 35% for **C1-Me** and 26% for **C1-F**, and 16% for **C2**. Notably, the only cage with a 4-pyridyl donor group (**C2**) has the lowest conversion at only 16%, mirroring the lower photo-conversion of **L3** compared with the 3-pyridyl ligands (Table S3†). Conversions are generally significantly higher than was observed in Fujita's Pd_12_L_24_ cage with *endo*-pendant azobenzenes, which had 17% *E*→*Z* conversion.^10^

The low degree of switching observed in Fujita's Pd_12_L_24_ cage was ascribed to the dominant absorption of a Pd-Py MLCT band in the same region as the azo π → π* band, and may also reflect constricted space inside the cage.^10^ Similar effects are likely to be a contributing factor for the cages reported here as the azo π → π* band overlaps with Ir(iii)-based mixed CT bands, but these do not appear to inhibit isomerisation to an excessive degree. The pattern of spectral changes we observe are very similar to those observed by Hecht *et al.* for a mononuclear [Ir(C^N)_2_(acac-AZB)] complex which showed photo-isomerisation to a photostationary state containing 46% *Z*-isomer.^33^ Mononuclear [Ir(C^N)_2_(NN)_*n*_]^+^ complexes with AZB-decorated ligands have been reported to have greatly reduced or even entirely suppressed *E* → *Z* photo switching due to low energy MLCT bands, which provide a pathway for relaxation of the π* azo excited state, but these studies featured very little reduction of the π → π* band on irradiation.^28^ This is clearly not the case here (Fig. 7) thus there is no evidence for competing photochemical pathways substantially suppressing *E* → *Z* isomerisation of these [{Ir(C^N)_2_}_3_(L)_2_]^3+^ coordination cages.

All cages could be converted back to the all-*E* form near quantitatively by irradiating at 450 nm for 15 minutes, Table S3 and Fig. S88–S92.† Repeated cycles of *E* → *Z* and *Z* → *E* photo-isomerisations were performed for **C2**, Fig S93.† The cage does experience a degree of photo-chemical fatigue across eight photo-switching cycles but the degree of *E* → *Z* switching remains unchanged, while recovery of the *E* form decreases suggesting a small amount of decomposition with repeated cycles of photo-isomerisation.

**Fig. 8:**
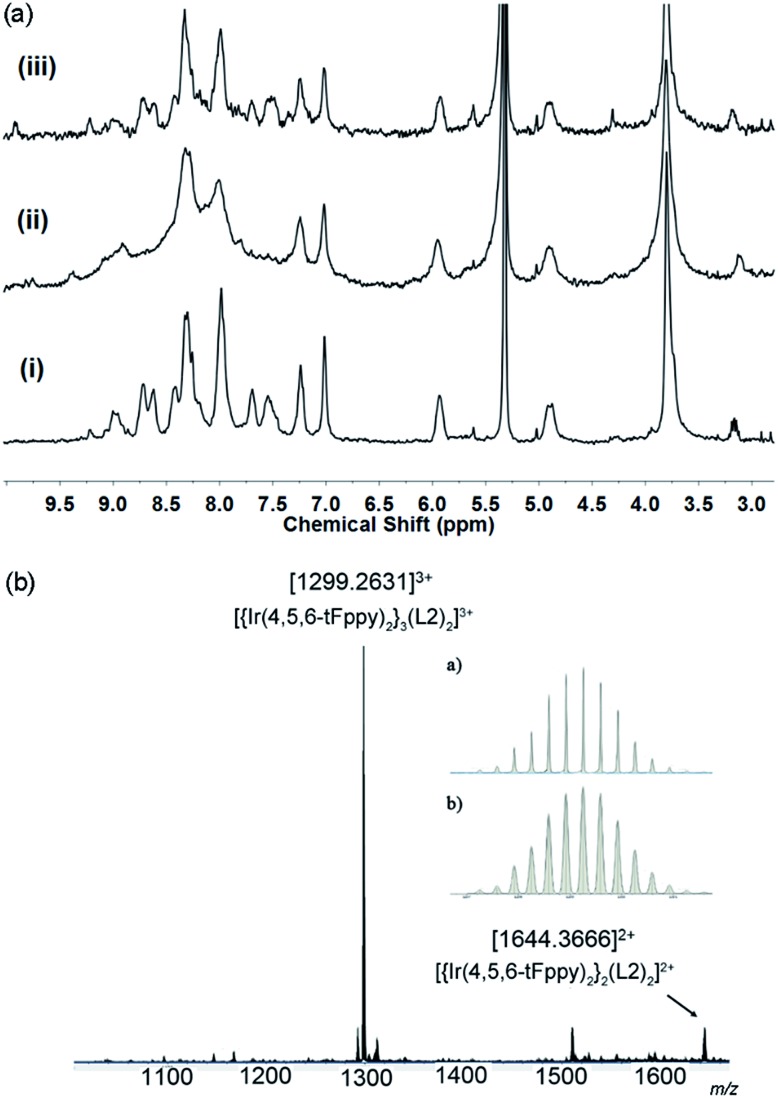
(a) ^1^H NMR (300 MHZ, CD_2_Cl_2_) of (i) initial **C1-F**; (ii) after irradiation with 355 nm laser for 200 s; (iii) after re-irradiation with 450 nm Xe lamp for 40 min. (b) ESI-MS of solution taken at conditions (ii).

Cage **C1-F** was the only cage sufficiently soluble in CD_2_Cl_2_ for ^1^H NMR studies. Its spectrum was recorded, re-recorded for the same sample after irradiation at 355 nm, then recorded again after further irradiation at 450 nm, Fig. 8. The spectrum after 355 nm irradiation is extremely broad and cannot be interpreted, however this is as expected for a mixture of cage isomers in solution, noting there are twelve possible *E*,*Z* isomers for an intact cage. The ESI-MS of this solution, Fig. 8b, is identical to the initial, un-switched cages and dominated by the 3+ peak at *m*/*z* 1229.2631 corresponding to [{Ir(4,5,6-tFppy)_2_}_3_(**L1**)_2_]^3+^. Hence, there are no compositional changes to the system. ^1^H DOSY NMR spectra of the 355 nm irradiated solution could not be obtained due to thermal *Z* → *E* relaxation that occurs over the long acquisition time required for DOSY. There is near complete conversion back to the initial spectrum on irradiation at 450 nm, Fig. 8a.

**Fig. 9:**
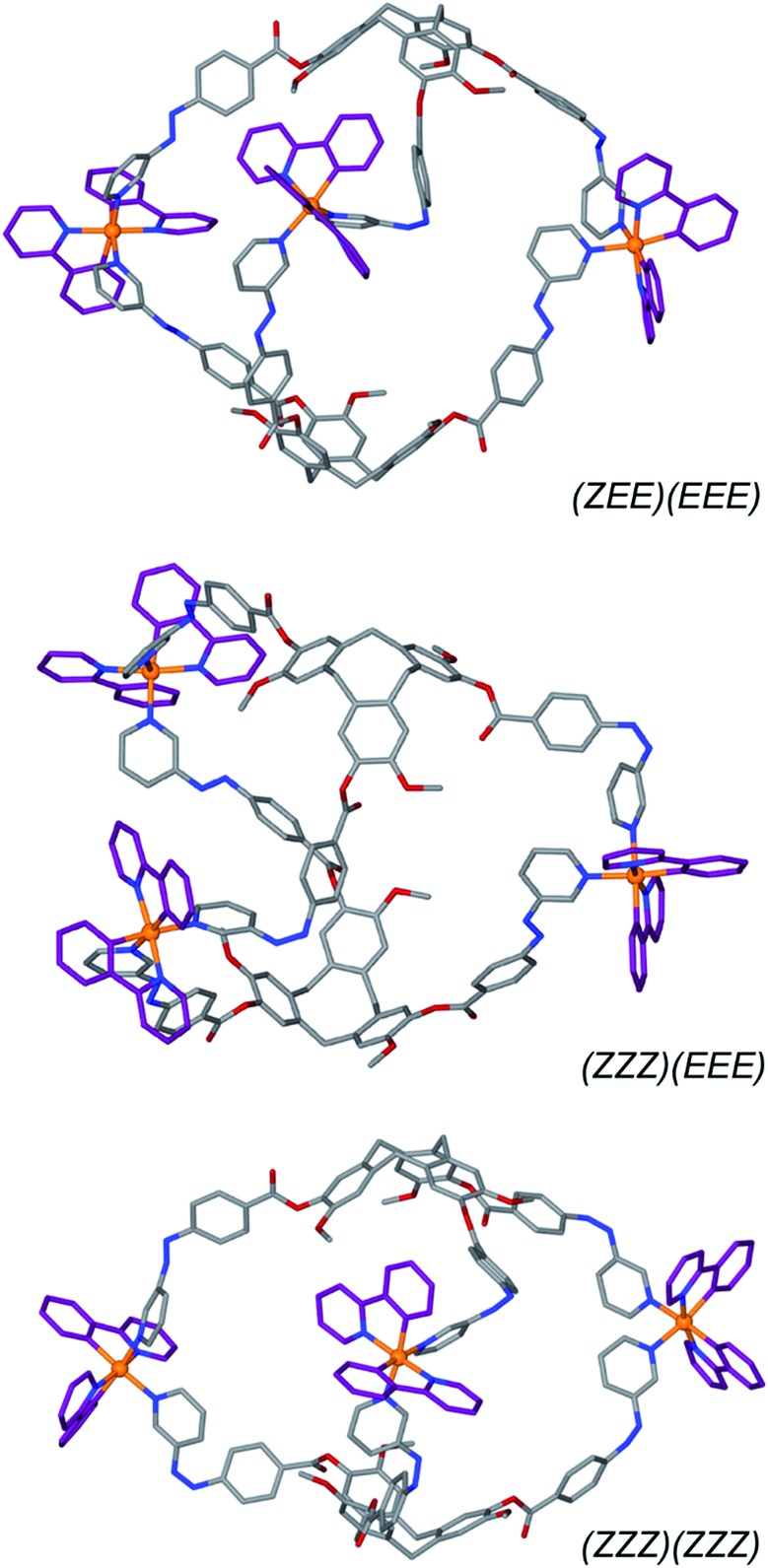
Energy-minimised models of [{Ir(ppy)_2_}_3_(**L2**)_2_]^3+^ with differing numbers of *Z-*isomer ligand arms. Hydrogens excluded for clarity.

To further demonstrate structural viability of the switched cage, energy-minimized molecular models were generated for the (*ZEE*)(*EEE*), (*ZZZ*)(*EEE*) and (*ZZZ*)(*ZZZ*) isomers of the cationic cage of **C1**. The resulting structures are given in Fig. 9. Introduction of *Z*-conformation pyridylazophenyl groups can be accommodated by rotations of the ester-linkages of the **L2** ligands. In the model of the all-*E* cage, the carbonyl groups are all *exo* to the cage, however the *Z*-containing cages all show a mixture of *exo* and *endo* orientations of these groups. Overall the *Z*-isomer-containing cages are smaller than the all-*E* cage with the distance between the centre of the three CTG-(CH_2_) groups reduced from 22.34 Å for the all-*E* isomer to 20.46 Å for the all-*Z* isomer.

### Emissive properties of coordination cages

**Fig. 10:**
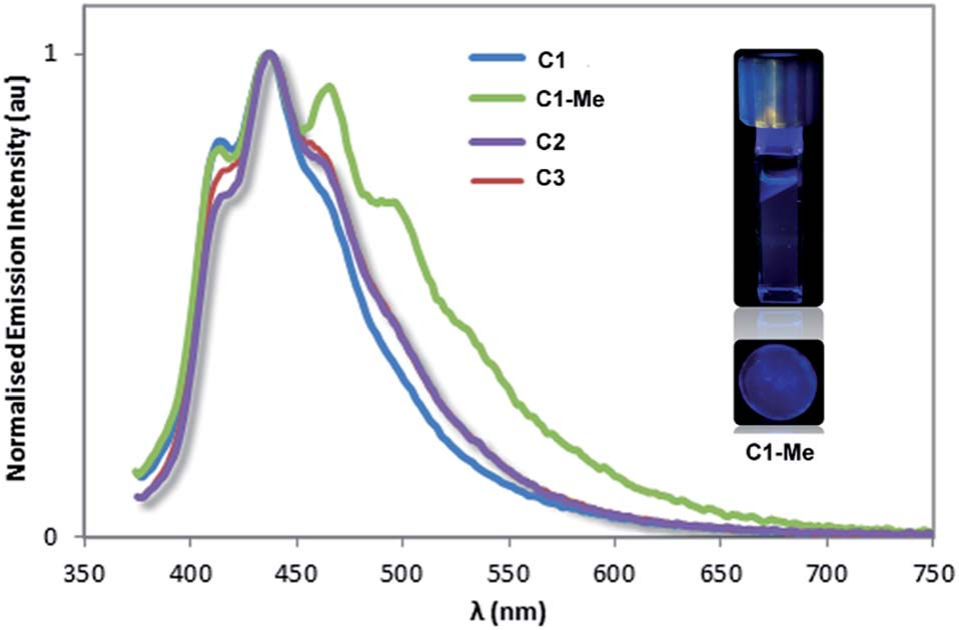
Normalized photoluminescence spectra of coordination cages in deaerated DCM solution. Inset shows darkroom images of emission of **C1-Me** in DCM (upper) and in PMMA matrix (lower).

[Ir^III^(C^N)_2_(A)_*n*_]^+^ species where A is either two monodentate or one bidentate ligand(s) represent an important subclass of emissive materials,^34^ and azobenzenes can themselves be weakly emissive. Freixa *et al.* showed that [Ir^III^(C^N)_2_(A)_*n*_]^+^ complexes bearing cyclometallated^35^ or bipyridyl appended^28^ AZB units have negligible luminescence owing to competitive electron transfer from the mixed CT triplet to the azo group. The emission properties of cages **C1**, **C1-Me**, **C2** and **C3** were determined in DCM solution, Fig. 10, and polymethylmethacrylate (PMMA)-doped films (5 wt% of cage, Fig. S99†), and results are summarised in Table 1. In deaerated DCM, all cages show similar deep blue structured emissions with maxima between 410 nm and 414 nm albeit with low photoluminescence quantum yields, *Φ*_PL_, of approximately 1%. These emissions are significantly blue-shifted compared with those of the previously reported [{Ir(ppy)_2_}_3_(L)_2_]^3+^ cages, which showed yellow-orange emission (*λ*_PL_ = 604 nm), or cyan emission (*λ*_PL_ = 485 nm) in DCM.^17^ DFT studies of similar mononuclear complexes indicate that the HOMOs of these coordination cages are likely located on the [Ir(C^N)_2_] moieties, whereas the LUMOs lie on the high-energy azobenzene fragment.^28^ Therefore, the presence of the AZB units attached to coordinating pyridines in **C1**, **C1-Me**, **C2** and **C3** implicate large HOMO–LUMO gaps and account for the deep-blue emissions exhibited by these cages. To the best of our knowledge, **C1**, **C1-Me**, **C2** and **C3** exhibit the bluest emissions reported for metallosupramolecular cages. On the other hand, the low *Φ*_PL_ values of these cages is probably the result of concomitant population of emissive π* orbitals involving the azobenzene ligand, access to non-radiative higher-lying metal-centred (MC) d–d states, which are located at similar energies, and non-radiative pathways associated with the conformationally flexible CTG-based ligands.^36^ Spin-coated PMMA-doped films of the four cage exhibited broad blue emissions, respectively, at *λ*_PL_ = 452 nm, *λ*_PL_ = 446 nm, *λ*_PL_ = 430 nm and *λ*_PL_ = 436 nm with low *Φ*_PL_ values between 0.7 and 1.7%, which are similar to those observed in DCM. However, as a result of the reduction of non-radiative vibration motion in the PMMA-doped films, the photoluminescence lifetimes, *τ*_PL_, of the ester-linked cages **C1** and **C1-Me** are significantly longer (2922 ns for **C1** and 3002 ns for **C1-Me**) compared to those in DCM (265 ns for **C1** and 275 ns for **C1-Me**). This is not the case for the glycol-linked cages **C2** and **C3**, which exhibit in both media short multi-exponential *τ*_PL_ in the nanosecond-time scale.

**Table 1:** Photophysical properties of cages

	*λ*_PL_ (nm)		*Φ*_PL_(%)^*c*^		*τ*_PL_	
	DCM^*a*^ ^,^^*b*^	Film^*d*^	DCM	Film^*d*^ ^,^^*e*^	DCM^*a*^ ^,^^*f*^ (ns)	Film^*d*^ ^,^^*f*^ (ns)
**C1**	414 (0.8), 434 (1), 466 (0.7)	452	1.3	1.7	6.0 (69), 20.8(13), 262.5 (18)	7.4 (49), 56.3 (10), 2922 (41)
**C1-Me**	414 (0.8), 438 (1), 464 (0.9)	446	1.5	1.9	7.2 (70), 24.0 (11), 274.9 (19)	7.4 (47), 56.9 (10), 3002 (43)
**C2**	410 (0.7), 432 (1), 464 (0.7)	430	0.8	0.9	1.5 (85), 4.5 (10), 13.1 (5)	1.7 (24), 9.2 (54), 54.8 (22)
**C3**	413 (0.7), 437 (1), 459 (0.8)	436	0.7	1.0	1.5 (82), 4.7 (12), 13.2 (6)	1.3 (41), 6.6 (48), 33 (11)

^*a*^Measurements in DCM under N_2_ at 298 K.

^*b*^Principal emission peaks listed with relative intensities in brackets.

^*c*^Quinine sulfate employed as the external reference (*Φ*_PL_ = 54.6% in 0.5 M H_2_SO_4_ at 298 K).^37^

^*d*^PMMA doped films (5 wt% of complexes). *Φ*_PL_ measurements were carried out under nitrogen.

^*e*^Values obtained using an integrating sphere.

^*f*^Values in parentheses are pre-exponential weighting factor, in relative % intensity, of the emission decay kinetics (*λ*_exc_ = 378 nm).

## Conclusions

Ligands **L2–L4** exhibits reversible photo-switching behavior, as do the family of [{Ir(C^N)_2_}_3_(L)_2_]^3+^ coordination cages. These cages represent the first examples of coordination cages with switchable AZB-type functionality that is inherent to the structural integrity of the cage. Remarkably, despite both the caged nature of these pyridyl-azo-phenyl groups, and overlap of the azo π → π* absorption with Ir(iii)-based mixed CT bands, extensive reversible *E* → *Z* photo-isomerization is observed. This occurs without compositional change. The degree of structural change required for *E* ↔ *Z* isomerization seen here is significantly greater than for ring-open ↔ ring-closed isomerization. The latter is the mechanism for the only previous example of a coordination cage exhibiting photo-isomerism of non-peripheral ligand groups.^3^ The remarkable stability of the [{Ir(C^N)_2_}_3_(L)_2_]^3+^ cages to large-scale reversible structure-switching is likely to be a function of both the high degree of rotational flexibility inherent in the **L2–L4** ligand designs, and the chemical inertness of Ir(iii) preventing dissociation of the cages. The rotational flexibility, however, contributes to the low photoluminescence quantum yields of these unusual blue-emitting cages. Cages form in a facile manner from [Ir(ppy)_2_]-precursors due to the trans-labilising nature of the ppy ligands.^24^ Once formed they are quite inert, demonstrated by the lack of spectral changes of these cages over several months in solution, as well as by previously studied heteroleptic ligand exchange in analogous [{Ir(ppy)_2_}_3_(L)_2_]^3+^ cages occurring only over months of equilibration.^17^

Given the wealth of interesting photophysical properties of many inert metal complexes, their use for coordination cage assembly is an area of considerable promise for expansion in general. The significant conformational and subsequent size changes that these [{Ir(C^N)_2_}_3_(L)_2_]^3+^ cages undergo, coupled with their luminescent properties, points to their future development as responsive hosts or shape-changing sensors, and their molecular recognition behavior is currently under investigation.

## Conflicts of interest

There are no conflicts to declare.

## Supplementary Material

Supplementary information

Crystal structure data
