# Supplementary material for: Structure-switching M3L2 Ir(iii) coordination cages with photo-isomerising azo-aromatic linkers
Source: Chem Sci. 2018 Sep 6;9(42):8150–9. doi: 10.1039/c8sc03499k (PMC6238882; doi:10.1039/c8sc03499k)
Supplement: Supplementary file 1 [file SC-009-C8SC03499K-s001.pdf]

Electronic Supplementary Material (ESI) for Chemical Science.
This journal is © The Royal Society of Chemistry 2018

Structure-switching M_3L_2 Ir(III) coordination cages with photo-isomerising azo-aromatic linkers

Samuel Oldknow,† Diego Rota Martir,§ Victoria E. Pritchard,† Mark A. Blitz,† Colin W. G. Fishwick,† Eli Zysman-Colman§ and Michaele J. Hardie†

† School of Chemistry, University of Leeds, Woodhouse Lane, Leeds LS2 9JT, UK. § Organic Semiconductor Centre, EaStCHEM School of Chemistry, University of St Andrews, St Andrews, Fife KY16 9ST, UK

Supplementary Information

Contents

1. **Experimental Procedures**
   1.1 Synthesis
   1.2 Crystal Structure Determinations (including additional Figures)
   1.3 Molecular Modelling
   1.4 Photoswitching Experiments
   1.5 Emission Studies
2. **Spectra supporting synthesis**
3. **Spectra and plots supporting photoswitching experiments**
   3.1 Ligand Photoswitching
   3.2 Coordination cage Photoswitching
4. **Spectra and images supporting emission studies**
5. **References**

1. Experimental Procedures

1.1 Synthesis

^1H and ^{13}C NMR spectra were recorded on a Bruker DPX 300MHz and a Bruker Ascend™ 400MHz NMR spectrometer. Where appropriate, routine NMR assignments were confirmed by 2D ^1H-^1H coupling experiments (COSY, HMQC, HMBC) which were all recorded on a Bruker Ascend™ 400MHz NMR spectrometer. 2-D ROESY ^1H-NMR experiments were performed using a Varian Unity Inova 500 spectrometer (Varian Inc., Palo Alto, California, USA) operating at 499.97 MHz proton frequency. Data were recorded at 298K using a 5 mm $^1H\{^{13}C, ^{15}N, ^{31}P\}$ ID/PFG VT probe. Phase-sensitive 2D ROESY experiments were performed with a mixing time of 300 ms, 64 transients, a relaxation delay of 4.0 s, 256 increments (states phase cycling, so 512 increments in total) and a spectral width of 6000 Hz and 2K data points. Data were processed using ACD Spectrus Processor 2015 of the ACD labs 2015 software package from Advanced Chemistry Development, (Toronto, Canada). DOSY measurements were made on a Jeol ECA 600ii 600 MHz spectrometer operating under regulated temperature conditions (20°C), with a 5mm probe. The pulse sequence is a bipolar pulse pair simulated echo (BPPSTE) operating in the ONESHOT experiment and spectra were processed using the DOSY toolbox. High-resolution electrospray mass spectra (ESI-MS) were recorded on a Bruker micro-TOF-Q mass spectrometer and FT-IR spectra were recorded as solid phase samples using a Perkin Elmer Spectrum One spectrometer. Melting points were recorded on a Stuart SMP3 melting point apparatus. Samples for microanalysis were dried under vacuum before analysis and determined by services at the University of Leeds or London Metropolitan University. UV-Visible spectra were recorded on a Lambda 900 UV/Vis spectrophotometer or an Agilent Cary 100 UV/Vis spectrophotometer.

Cyclotriguaiacylene,[1] 4-nitroso methylbenzoate,[2] *p*-(4-pyridylazo)phenol,[3] *p*-(3-pyridylazo)phenol,[4] 2,7,12-tris-(2-bromoethoxy)-3,8,13-trimethoxy-10,15-dihydro-2H-tribenzo[*a,d,g*]cyclononene,[5] $[Ir(C\text{^}N)_2(NCMe)_2](BF_4)$ where C^N = 2,phenylpyridine (ppy),[6] 2-(4-methylphenyl)pyridine (Meppy)[7] or

2-(4,5,6-trifluorophenyl)pyridine (4,5,6-tFppy)[8] were synthesised by literature procedures. Where stated, reactions were carried out under an inert atmosphere of argon using an argon/vacuum dual manifold and standard Schlenk techniques. All chemicals and solvents were purchased from commercial suppliers (Sigma, Fluka) and were used as received. Dry solvents were obtained by passing through a column of activated alumina. Argon and nitrogen gas were pre-dried by passing through a small column of P_2O_5 before use. Coordination cages were insufficiently soluble for their ^{13}C NMR spectra to be obtained.

Sodium 4-[2-(4-pyridyl)diazenyl]-benzoate (1p)

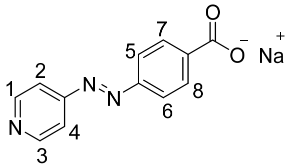

Sodium hydroxide (3.00 g, 75 mmol) was dissolved in 100 mL of water to form a 3% NaOH solution. 4-Aminopyridine (0.52 g, 5.6 mmol) and 4-nitroso methylbenzoate (0.5 g, 3.0 mmol) were added and the mixture was heated to reflux overnight. The resulting bright orange solution was then cooled to room temperature resulting in the formation of a bright orange precipitate. The solid was filtered and dried *in vacuo* to give the product (0.53g, 2.19 mmol, 73%) as an orange solid. Single crystals with formula **1p**·4(H_2O) were obtained by recrystallization from water. M.pt >300˚C; ^1H NMR (300 MHz, DMSO-d^6) δ (ppm) 8.83 (2H, dd, J 6.0, 1.8, Ar-H (1), Ar-H (3)), 8.04 (2H, dt, J 9.0, 1.8, Ar-H (7), Ar-H (8)), 7.85 (2H, d, J 9.0, 1.8, Ar-H (5), Ar-H (6)), 7.76 (2H, d, J 6.0, 1.8, Ar-H (2), Ar-H (4)); ^{13}C NMR (75 MHz, MeOD-d^4) δ (ppm) 159.26, 151.80, 131.24, 123.81, 117.76. HR-MS (ES$^+$) m/z 228.0766 {M + H}$^+$ (calculated for $\{C_{12}H_{10}N_3O_2\}^+$ 228.0768); FT-IR (cm^{-1}) = 3241, 1676, 1591, 1536, 1381, 1306, 1224, 1095, 830, 792, 691, 598, 588. 506; CHN analysis for $Na(C_{12}H_8N_3O_2)(H_2O)_4$ (% calc.; found) C (44.86, 44.52), H (5.02, 4.67), N (13.08, 12.98); UV-Vis (DMSO) λ_{max} (nm) 315 (π → π*), 445 (n → π*).

Sodium 4-[2-(3-pyridyl)diazenyl]-benzoate (2p)

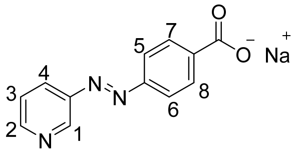

Sodium hydroxide (3.00 g, 75 mmol) was dissolved in 100 mL of water to form a 3% NaOH solution. 3-Aminopyridine (1.48 g, 15.7 mmol) and 4-nitroso methylbenzoate (1.40 g, 8.47 mmol) were added and the mixture was heated to reflux overnight. The resulting red solution was then cooled to room temperature resulting in the formation of orange-red crystals. The crystals were filtered and washed with acetone and diethyl ether to give the product (2.07 g, 6.10 mmol, 72% for hydrate) as a bright orange solid. M.pt >300˚C; ^1H NMR (300 MHz, D_2O) δ (ppm) 9.07 (1H, dd, J 2.4, 0.7, Ar-H (1)), 8.70 (1H, dd, J 4.9, 1.5, Ar-H (2)), 8.28 (1H, m, Ar-H (4)), 8.05 (2H, dt, J 8.7, 1.8, Ar-H (7), Ar-H (8)), 7.95 (2H, dt, J 8.7, 2.1, Ar-H (5), Ar-H (6)), 7.67 (1H, m, J 8.3, Ar-(3)); Over time in solution also observe new peaks corresponding to *cis (Z)* isomer: ^1H NMR (300 MHz, D_2O) δ (ppm) 8.30 (1H, dd, J 4.2, 2.3), 8.21 (1H, dd, J 2.1, 1.2), 7.73 (2H, dt, J 8.4, 1.8), 7.34 – 7.31 (2H, m), 6.92 (2H, dt, J 8.4, 2.1); ^{13}C NMR (75 MHz, D_2O) δ_C 156.19, 153.30, 151.13, 147.83, 145.08, 139.45, 129.88, 128.63, 124.95, 122.43; HR-MS (ES$^+$) m/z 228.0780 {M + H}$^+$ (calculated for $\{C_{12}H_{10}N_3O_2\}^+$ 228.0768); FT-IR (cm^{-1}) = 1593, 1539, 1385, 1310, 1220, 1099, 1014, 810, 786, 700, 622, 591; Analysis calculated for **2p**.0.5 H_2O (% calculated; found) C (55.82, 56.20), H (3.51, 3.20), N (16.27, 16.50); UV-Vis (DMSO) λ_{max} (nm) 326 (π → π*), 440 (n → π*).

(±)-2,7,12-Trimethoxy-3,8,13-*tris*(4,4'-pyridyl-azophenylcarboxy)-10,15-dihydro-*5H*-tribenzo[*a,d,g*] cyclononene (L1)

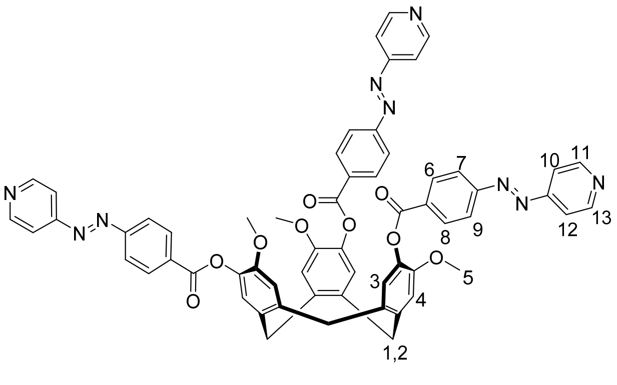

Step 1: Sodium 4-[2-(4-pyridyl)diazenyl]-benzoate benzoate (0.5 g, 2.20 mmol) was suspended in thionyl chloride (15 mL) under an argon atmosphere. The resulting red suspension was heated to reflux overnight. The flask was allowed to cool to room temperature before the thionyl chloride was removed *in vacuo* to give 4,4'-pyridyl-azo-benzoyl chloride as a pale red solid that was used immediately without further purification.

Step 2: CTG (0.11 g, 0.27 mmol) was dissolved in dry THF (100 mL) under argon. The flask was cooled to 0°C in an ice bath and triethylamine (15 mL) was added. The resulting yellow solution was stirred at 0°C for 1 hour before being added dropwise via cannula to solid 4,4'-pyridyl-azo-benzoyl chloride (0.49 g, 2.20 mmol) under argon. The resulting red-orange suspension was allowed to stir at room temperature for 3 days. The solids were filtered off and triturated in methanol (100 mL) and then THF (100 mL) to give the product (0.22 g, 0.21 mmol, 79%) as a yellow-orange solid. M.pt 259-261°C; ^{1}H NMR (300 MHz, DMSO-d^{6}) δ (ppm) 8.89 (6H, d, J 6.0, Ar-H (11), Ar-H (13)), 8.36 (6H, d, J 9.0, Ar-H (6), Ar-H (8)), 8.13 (d, 6H, J 9.0, Ar-H (7), Ar-H (9)), 7.83 (6H, d, J 6.0, Ar-H (10), Ar-H (12)), 7.61 (3H, s, Ar-H (3)), 7.37 (3H, s, Ar-H (4)), 4.93 (3H, d, J 13.7, CTG endo-H (1)), 3.79 – 3.71 (12H, m, O-CH_3 (5) overlapped with CTG exo-H (2)); ^{13}C NMR (75 MHz, $CDCl_3$) δ (ppm) 163.92, 156.91, 154.92, 151.54, 151.46, 149.89, 138.54, 138.18, 132.36, 131.79, 131.53, 131.46, 124.03, 123.26, 116.32, 114.30, 77.44, 77.22, 77.02, 76.59, 56.31, 36.56; HR-MS (ES^{+}) m/z 2072.6807 {2M + H}$^{+}$ (calculated for {$C_{120}H_{91}N_{18}O_{18}$}$^{+}$ 2072.6787), 1555.0155 {3M + 2H}$^{2+}$ (calculated for {$C_{180}H_{137}N_{27}O_{27}$}$^{2+}$ 1555.0117) 1053.3935 {M + H_2O}$^{+}$ (calculated for {$C_{60}H_{47}N_9O_{10}$}$^{+}$ 1053.3446) 1036.3418 {M + H}$^{+}$ (calculated for {$C_{60}H_{46}N_9O_9$}$^{+}$ 1036.3413); FT-IR (cm^{-1}) = 3030, 2931, 1721, 1584, 1505, 1476, 1461, 1405, 1258, 1060, 1005, 941, 924, 893, 860, 825, 683, 555; Analysis calculated for **L1**.$CHCl_3$.H_2O (% calculated; found) C (62.44, 62.40), H (4.12, 4.33), N (10.74, 10.84); UV-Vis (DMSO) λ_{max} (nm) 312 (π → π*), 465 (n → π*).

(±)-2,7,12-Trimethoxy-3,8,13-*tris*(4,3'-pyridyl-azophenylcarboxy)-10,15-dihydro-*5H*-tribenzo[*a,d,g*]cyclononene (L2)

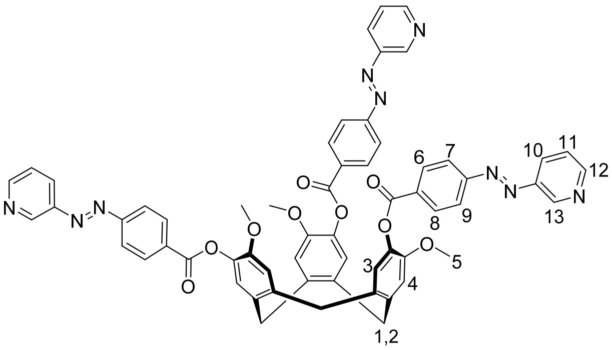

Step 1: Sodium 4-[2-(3-pyridyl)diazenyl]-benzoate benzoate (1.75 g, 7.03 mmol) was suspended in thionyl chloride (15 mL) under an argon atmosphere. The resulting red suspension was heated to reflux overnight. The flask was allowed to cool to room temperature before the thionyl chloride was removed *in vacuo* to give 4,3'-pyridyl-azo-benzoyl chloride as a pale red solid that was used immediately without further purification.

Step 2: CTG (0.52 g, 1.27 mmol) was dissolved in dry THF (100 mL) under argon. The flask was cooled to 0°C in an ice bath and triethylamine (15 mL) was added. The resulting yellow solution was stirred at 0°C for 1 hour before being added dropwise via cannula to solid 4,3'-pyridyl-azo-benzoyl chloride (1.89 g, 7.70 mmol). The resulting red-orange suspension was allowed to stir at room temperature for 3 days. The solids were filtered off and the solvent removed in vacuo to yield a red-orange residue. This crude product was triturated in methanol (2 x 100 mL) to give the product (1.16 g, 1.12 mmol, 88%) as a fine orange solid. M.pt 250-252˚C; ^{1}H NMR (300 MHz, $CDCl_3$) δ (ppm) 9.26 (3H, d, J 2.5, Ar-H (13)), 8.76 (3H, dd, J 4.6, 1.7, Ar-H (12)), 8.38 (6H, d, J 8.5, Ar-H (6), Ar-H (8)), 8.21 (3H, dt, J 8.3, 1.9, Ar-H (10)), 8.05 (6H, d, J 8.5, Ar-H (7), Ar-H (9)), 7.48 (3H, dd, J 8.2, 4.7, Ar-H (11)), 7.22 (3H, s, Ar-H (3)), 7.00 (3H, s, Ar-H (4)), 4.87 (3H, d, J 13.7, CTG endo-H (1)), 3.82 (9H, s, OCH_3 (5)), 3.72, d, J 13.9, CTG exo-H (2)); ^{13}C NMR (75 MHz, $CDCl_3$) δ (ppm) 164.19, 155.30, 152.52, 150.07, 147.91, 147.85, 138.73, 138.31, 131.95, 131.68, 131.59, 127.23, 124.22, 123.12, 114.45, 56.46, 36.68; HR-MS (ES^{+}) m/z 1036.3463{M + H}$^{+}$ (calculated for {$C_{60}H_{46}N_9O_9$}$^{+}$ 1036.3413), 518.6194 {M + 2H}$^{2+}$ (calculated for {$C_{60}H_{46}N_9O_9$}$^{2+}$ 518.6743); FT-IR (cm^{-1}) = 3066, 2933, 2833, 1786, 1724, 1601, 1583, 1506, 1323, 1260,

1177, 1061, 1006, 859, 764, 697; Analysis calculated for **L2**.H_2O (% calculated; found) C (68.37, 68.10), H (4.49, 4.30), N (11.96, 11.90); UV-Vis (DMSO) λ_{max} (nm) 326 (π → π*), 411 (n → π*).

2,7,12-Tris-(2-(4-pyridylazo)ethoxy)-3,8,13-trimethoxy-10,15-dihydro-2H-tribenzo[*a,d,g*]cyclononene (L3)

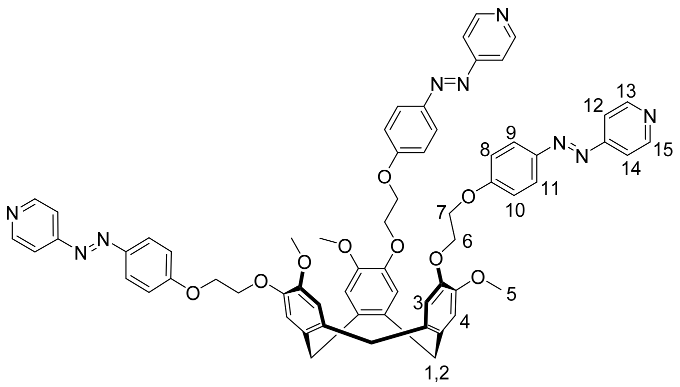

p-(4-Pyridylazo)phenol (1.11 g, 5.55 mmol), 2,7,12-tris-(2-bromoethoxy)-3,8,13-trimethoxy-10,15-dihydro-2H tribenzo [a,d,g]cyclononene (0.45 g, 0.62 mmol) and caesium carbonate (2.65 g, 8.16 mmol) were dissolved in anhydrous DMF (10 mL) under an argon atmosphere. The resulting bright red solution was heated to 100°C for 3 days. The reaction was cooled to room temperature and water (90 mL) was added slowly. The resulting reddy-brown precipitate was filtered off and washed with methanol (50 mL) and diethyl ether (50 mL) to give a crude yellow-orange solid. This crude product was triturated in DCM (100 mL), filtered and the solvent removed under reduced pressure to give the product (0.58 g, 0.53 mmol, 85%) as an orange crystalline powder. M.pt 232-234˚C; ^{1}H NMR (300 MHz, $CDCl_3$) δ (ppm) 8.77 (6H, d, J 6.0, Ar-H (13), Ar-H (15)), 7.93 (6H, d, J 9.0, Ar-H (9), Ar-H (11)), 7.66 (6H d, J 6.0, Ar-H (12), Ar-H (14)), 7.02 (6H, d, J 9.0, Ar-H (8), Ar-H (10)), 6.97 (3H, s, Ar-H (3)), 6.85 (3H, s, Ar-H (4)), 4.76 (3H, d, J 13.9, CTG endo-H (1)), 4.43 – 4.32 (12 H, m, OCH_2CH_2 (6,7)), 3.78 (9H, s, OCH_3 (5)), 3.56 (3H, d, J 13.8, CTG exo-H (2)); ^{13}C NMR (75 MHz, $CDCl_3$) δ (ppm) 162.19, 157.35, 151.23, 148.84, 147.01, 146.71, 133.48, 131.95, 125.54, 117.32, 116.15, 115.03, 114.14, 68.32, 66.96, 56.28, 36.49; HR-MS (ES^+) m/z 1084.4333 {M + H}$^+$ (calculated for $\{C_{63}H_{58}N_9O_9\}^+$ 1084.4352), 542.7416 {M + 2H}$^{2+}$ (calculated for $\{C_{63}H_{58}N_9O_9\}^{2+}$ 542.7212); FT-IR (cm^{-1}) = 3348, 2930, 1598, 1582, 1497, 1452, 1417, 1402, 1250, 1214, 1137, 1089, 1058, 1042, 1001, 989, 922, 834, 743, 623, 559, 518; Analysis calculated for **L3**. DCM (% calculated; found) C (65.75, 65.80), H (5.09, 5.05), N (10.78, 10.40) UV-Vis (DMSO) λ_{max} (nm) 354 (π → π*, E isomer), 445 (n → π*, Z isomer).

2,7,12-Tris-(2-(3-pyridylazo)ethoxy)-3,8,13-trimethoxy-10,15-dihydro-2H-tribenzo[*a,d,g*]cyclononene (L4)

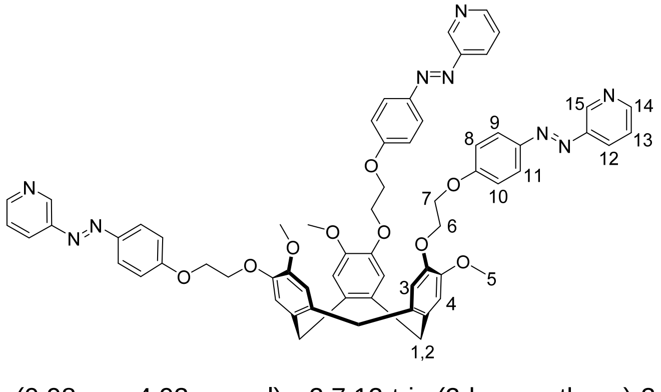

p-(3-Pyridylazo)phenol (0.98 g, 4.93 mmol), 2,7,12-tris-(2-bromoethoxy)-3,8,13-trimethoxy-10,15-dihydro-2H tribenzo [a,d,g]cyclononene (0.40 g, 0.54 mmol) and caesium carbonate (2.32 g, 7.14 mmol) were dissolved in anhydrous DMF (10 mL) under an argon atmosphere. The resulting bright red solution was heated to 100°C for 3 days. The reaction was cooled to room temperature and water (90 mL) was added slowly. The resulting reddy-brown precipitate was filtered off and washed with methanol (50 mL) and diethyl ether (50 mL) to give a crude yellow-orange solid. This crude product was triturated in DCM

(100 mL), filtered and the solvent removed under reduced pressure to give the product (0.32 g, 0.29 mmol, 55%) as a yellow solid. M.pt 159-161˚C; ^{1}H NMR (400 MHz, $CDCl_3$) δ (ppm) 9.14 (3H, d, J 2.3, Ar-H (15)), 8.66 (3H, dd, J 4.7, 1.7, Ar-H (14)), 8.10 (3H, dt, J 8.2, 2.0, Ar-H (12)), 7.92 (6H, dt, J 6.7, 2.7, Ar-H (9), Ar-H (11)), 7.42 (3H, dd, J 8.1, 4.7, Ar-H (13)), 7.01 (6H, dt, J 6.9, 1.5, Ar-H (8), Ar-H (10)), 6.97 (3H, s, Ar-H (3)), 6.85 (3H, s, Ar-H (4)), 4.77 (3H, d, J 13.7, CTG endo-H (1)), 4.43 – 4.32 (12H, m, OCH_2CH_2 (6,7)), 3.78 (9H, s, OCH_3 (5)), 3.57 (3H, d, J 13.8, CTG exo-H (2)); ^{13}C NMR (101 MHz, $CDCl_3$) δ (ppm) 161.76, 151.25, 148.98, 148.09, 147.27, 147.18, 146.86, 133.60, 132.08, 126.89, 125.19, 124.03, 117.46, 115.10, 114.25, 77.16, 68.48, 67.04, 56.41, 36.63; HR-MS (ES$^+$) m/z. 1084.4359 {M + H}$^+$ (calculated for $\{C_{63}H_{58}N_9O_9\}^+$ 1084.4352), 542.7206 {M + 2H}$^{2+}$ calculated for $\{C_{63}H_{59}N_9O_9\}^{2+}$ 542.7212); FT-IR (cm^{-1}) = 3432, 2924,1598, 1582, 1498, 1452, 1398, 1313, 1250, 1217, 1140, 1061, 1019, 1000, 918, 836, 809, 743, 723, 701, 616, 552, 518; Analysis calculated for **L4**. 0.5 DCM (% calculated; found) C (67.75, 67.60), H (5.33, 5.15), N (11.11, 10.73); UV-Vis (DMSO) λ_{max} (nm) 354 (π→π* trans (E) isomer), 448 (n→π* cis (Z) isomer).

$[\{Ir(ppy)_2\}_3(L2)_2]^{3+}\cdot 3PF_6^-$ coordination cage (C1)

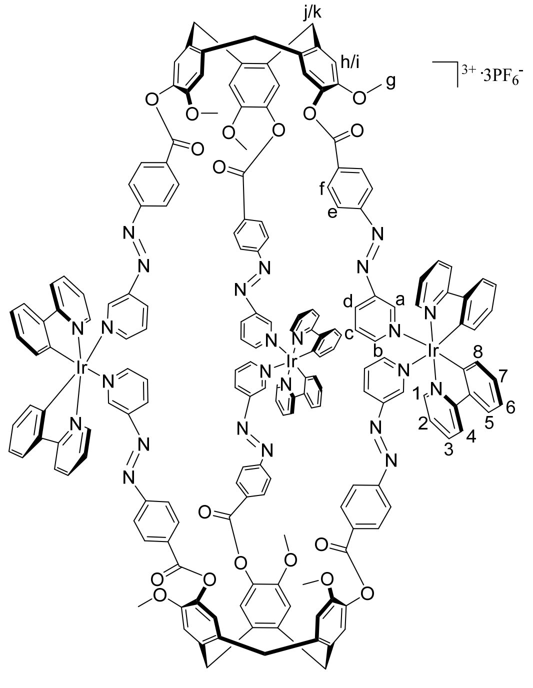

$[Ir(ppy)_2(MeCN)_2]^+\cdot PF_6^-$ (26.3 mgs, 0.037 mmol) and **L2** (25.0 mgs, 0.024 mmol) were suspended in nitromethane (5 mL). The orange suspension was stirred at room temperature overnight during which time all of the material dissolved. Diethyl ether was added slowly to the solution which resulted in the precipitation of a fine orange powder. The solid was filtered and washed with more diethyl ether to give the product (32.1 mgs, 0.008 mmol, 66%) as an orange powder. ^{1}H NMR (300 MHz, $MeNO_2$-d^3) δ (ppm) 9.29 (6H, br s, Ar-H (a)), 8.93 (12 H, br s, Ar-H (b) overlapped with Ar-H (1)), 8.39 (18H, br s, Ar-H (d) overlapped with Ar-H (f)), 8.03 (24H, s, Ar-H (e) overlapped with Ar-H (3) and Ar-H (4)), 7.70 (12H, br s, Ar-H (c) overlapped with Ar-H (5)), 7.46 (12H, br s, CTG Ar-H (h) overlapped with Ar-H (2)), 7.27 (6H, br s, CTG Ar-H (i)), 7.01 (12H, br s, Ar-H^6 overlapped with Ar-H^7), 6.58 (6H, br s, Ar-H^8), 5.02 (6H, s, CTG endo-H (j)), 3.83 (24H, br s, CTG exo-H (k) overlapped with OCH_3 (g)); HR-MS (ES$^+$) m/z 1536.4289 $\{M_2L_2\}^{2+}$ (calculated for $\{C_{164}H_{124}Ir_2N_{22}O_{18}\}^{2+}$ 1536.9361), 1191.3193$\{M_3L_2\}^{3+}$ (calculated for $\{C_{186}H_{140}Ir_3N_{24}O_{18}\}^{3+}$ 1191.3179); FT-IR (cm^{-1}) = 3061, 1732, 1582, 1505, 1478, 1420, 1256, 1176, 1086, 1057, 1009, 835, 756, 735, 697, 586; Analysis indicates incomplete desolvation of material, calculated for **C1** (% calculated; found) C (55.73, 51.01), H (3.47, 3.39), N (8.39, 8.15); UV-Vis λ_{max} (nm) 280 (Intraligand π→π* transitions), 320 (π→π* E isomer), 425 (n→π* Z isomer).

[{Ir(Meppy)$_2$}$_3$(L2)$_2$]$^{3+}$·3PF$_6^-$ coordination cage (C1-Me)

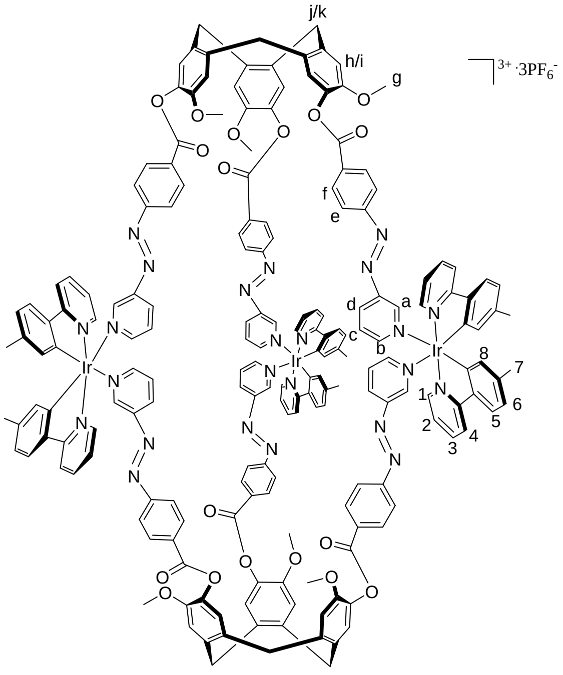

[Ir(Meppy)$_2$(MeCN)$_2$]$^+$·PF$_6^-$ (26.3 mgs, 0.037 mmol) and **L2** (25.0 mgs, 0.024 mmol) were suspended in nitromethane (5 mL). The orange suspension was stirred at room temperature overnight during which time all of the material dissolved. Diethyl ether was added slowly to the solution which resulted in the precipitation of a fine orange powder. The solid was filtered and washed with more diethyl ether to give the product (30.5 mgs, 0.007 mmol, 63%) as an orange powder. ^{1}H NMR (300 MHz, MeNO$_2$-d^3) δ (ppm) 9.27 (6H, br s, Ar-H (a), 8.91 (12 H, br s, Ar-H (b) overlapped with Ar-H (1)), 8.44 (6H, br s, Ar-H (d)) 8.36 (18H, s, Ar-H (f)), 8.00 (24H, br s, Ar-H (e) overlapped with Ar-H (3) and Ar-H (4)), 7.69 (6H, br s, Ar-H (c)), 7.59 (6H, br d, Ar-H (5)), 7.45 (12H, br s, CTG Ar-H (h) overlapped with Ar-H (2)), 7.27 (6H, br s, CTG Ar-H (i)), 6.87 (6H, br s, Ar-H (6)), 6.45 (6H, br s, Ar-H (8)), 5.02 (6H, s, CTG endo-H (j)), 3.84 (24H, br s, CTG exo-H (k) overlapped with OCH$_3$ (g)), 2.20 (18H, br s, Ar-CH$_3$ (7)); HR-MS (ES$^+$) m/z 1219.3517{M$_3$L$_2$}$^{3+}$ (calculated for {C$_{192}$H$_{152}$Ir$_3$N$_{24}$O$_{18}$}$^{3+}$ 1219.3492) ; FT-IR (cm^{-1}) = 2918, 1734, 1605, 1587, 1563, 1464, 1257, 1205, 1176, 1087, 1058, 1009, 836, 768, 698, 555; Analysis calculated for **C1-Me** (% calculated; found) C (56.34, 57.83), H (3.69, 3.77), N (8.21, 7.83); UV-Vis λ_{max} (nm) 258 (Intraligand π→π* transitions), 312 (π→π* E isomer), 426 (n→π* Z isomer).

[{Ir(4,5,6-tFppy)$_2$}$_3$(L2)$_2$]$^{3+}$·3PF_6^- coordination cage (C1-F)

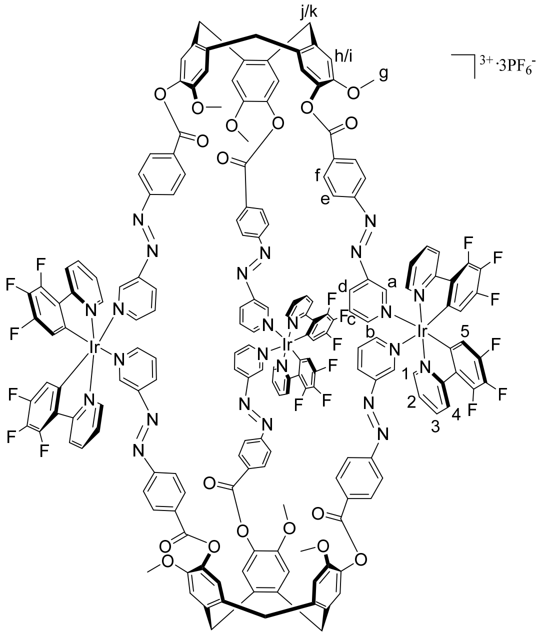

[Ir(4,5,6-tFppy)$_2$(MeCN)$_2$]$^+$·PF_6^- (30.2 mgs, 0.036 mmol) and **L2** (25.0 mgs, 0.024 mmol) were suspended in nitromethane (5 mL). The orange suspension was stirred at room temperature overnight during which time all of the material dissolved. Diethyl ether was added slowly to the solution which resulted in the precipitation of a fine orange powder. The solid was filtered and washed with more diethyl ether to give the product (47.6mgs, 0.012mmol, 50%) as an orange crystalline powder. ^{1}H NMR (300 MHz, $MeNO_2$) δ (ppm) 9.19 (6H, q, Ar-H (a)), 8.99 (6H br s, Ar-H (1)), 8.84 (6H br s, Ar-H (b)), 8.47 (6H, br s, Ar-H (d)), 8.40 – 8.25 (18H, m, Ar-H (f) overlapped with Ar-H (4)), 8.16 -7.90 (18H, m, Ar-H (e) overlapped with Ar-H (3)), 7.72 (6H br s, Ar-H (2)), 7.60 (6H, br s, Ar-H (c)), 7.44 (6H, br s, CTG Ar-H (h)), 7.27 (6H, br s, CTG Ar-H (i)), 6.15 (6H br s, Ar-H (5)), 5.03 (6H d, *J* 4.5, CTG exo-H (j)); 3.84 (24H, br s, CTG endo-H (k) overlapped with OCH_3 (g); HR-MS (ES$^+$) m/z 1299.5033 {M_3L_2}$^{3+}$ (calculated for {$C_{186}H_{122}F_{18}Ir_3N_{24}O_{18}$}$^{3+}$ 1299.2613); FT-IR (cm^{-1}) = 2932, 1733, 1667, 1603, 1587, 1555, 1507, 1486, 1431, 1400, 1375, 1318, 1257, 1204, 1176, 1137, 1087, 1060, 1042, 914, 804, 758, 699, 653, 623, 574, 447; Analysis calculated for **C1-F** (% calculated; found) C (51.56, 51.49), H (2.79, 2.90), N (7.76, 7.80)UV-Vis λ_{max} (nm) 262 (Intraligand π→π* transitions), 290 (Intraligand π→π* transitions), 324 (π→π* *E* isomer), 432 (n→π* *Z* isomer).

[{Ir(ppy)$_2$}$_3$(L3)$_2$]$^{3+}\cdot$3PF$_6^-$ coordination cage (C2)

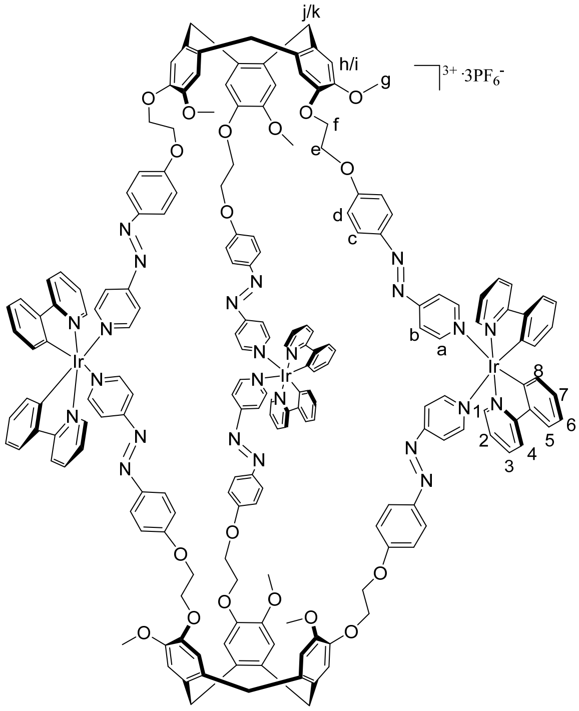

[Ir(ppy)$_2$(MeCN)$_2$]$^+\cdot$PF$_6^-$ (26.3 mgs, 0.037 mmol) and **L3** (25.0 mgs, 0.024 mmol) were suspended in nitromethane (5 mL). The orange suspension was stirred at room temperature overnight during which time all of the material dissolved. Diethyl ether was added slowly to the solution which resulted in the precipitation of a fine orange powder. The solid was filtered and washed with more diethyl ether to give the product (35.3 mgs, 0.009 mmol, 75%) as an orange powder. ^{1}H NMR (300 MHz, MeNO$_2$-d^3) δ (ppm) 8.77 (18H, br s, Ar-*H* (a) overlapped with Ar-*H* (1)), 8.00 (24H, br s, Ar-*H* (c) overlapped with Ar-*H* (3) and Ar-*H* (4)), 7.66 (18H, br s, Ar-*H* (b) overlapped with Ar-*H* (5)), 7.45 (6H, br s, Ar-*H* (2)), 7.09 (24H, br s, Ar-*H* (d) overlapped with Ar-*H* (6) and Ar-*H* (7)), 6.95 (12H, br s, CTG Ar-H (h) overlapped with CTG Ar-H (i)), 6.50 (6H, br s, Ar-*H* (8)), 4.84 (6H, br s, CTG endo-H (j)), 4.44 (24H, br s, OCH$_2$CH$_2$ (e,f – overlaps with MeNO$_2$ signal)), 3.78 (18H, br s, OCH$_3$ (g)), 3.65 (6H, br s, CTG exo-H (k)); HR-MS (ES$^+$) m/z 1584.5199 {M$_2$L$_2$}$^{2+}$ (calculated for {C$_{170}$H$_{148}$Ir$_2$N$_{22}$O$_{18}$}$^{2+}$ 1584.0222) 1223.3784{M$_3$L$_2$}$^{3+}$ (calculated for {C$_{192}$H$_{164}$Ir$_3$N$_{24}$O$_{18}$}$^{3+}$ 1223.3805); FT-IR (cm^{-1}) = 3041, 2929, 1594, 1582, 1497, 1477, 1446, 1405, 1252, 1135, 1089, 926, 831, 755, 732, 628, 555; Analysis calculated for **C2** (% calculated; found) C (54.20, 54.50), H (3.80, 4.19), N (7.60, 7.94); UV-Vis λ$_{max}$ (nm) 263 (Intraligand π→π* transitions), 323 (π→π* *E* isomer), 436 (n→π* *Z* isomer).

[{Ir(ppy)$_2$}$_3$(L4)$_2$]$^{3+}$·3PF$_6^-$ coordination cage (C3)

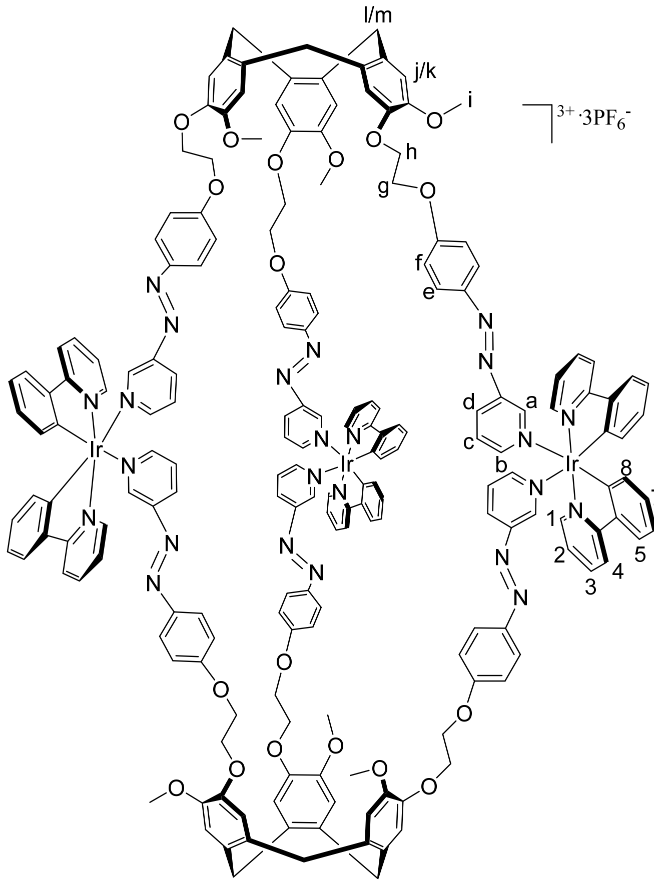

[Ir(ppy)$_2$(MeCN)$_2$]$^+$·PF$_6^-$ (26.3 mgs, 0.037 mmol) and **L4** (25.0 mgs, 0.024 mmol) were suspended in nitromethane (5 mL). The orange suspension was stirred at room temperature overnight during which time all of the material dissolved. Diethyl ether was added slowly to the solution which resulted in the precipitation of a fine orange powder. The solid was filtered and washed with more diethyl ether to give the product (35.3 mgs, 0.009 mmol, 75%) as a yellow-orange powder. ^{1}H NMR (300 MHz, MeNO$_2$) δ (ppm) 9.13 (6H, m, Ar-H (a)), 8.83 (12H, m, Ar-H (b) overlapped with Ar-H (1)), 8.27 (6H, br s, Ar-H (d)), 8.10 – 7.37 (42H, br m, Ar-H (e) and Ar-H(c) overlapped with with Ar-H (3), Ar-H (4), Ar-H (5) and Ar-H (2)), 7.02 (36H, m, Ar-H (f), CTG-H (j) and CTG-H (k) overlapped with Ar-H (6) and Ar-H (7)), 6.54 (6H, br s, Ar-H (8)), 4.83 (6H, br s, CTG endo-H (l)), 3.77 (24H, m, CTG exo-H (m) overlapped with OCH$_3$ (i)); HR-MS (ES$^+$) m/z 1584.5232 {ML}$^+$ (calculated for C$_{85}$H$_{73}$IrN$_{11}$O$_9$}$^+$ 1584.0222) 1223.7167 {M$_3$L$_2$}$^{3+}$ (calculated for {C$_{192}$H$_{164}$Ir$_3$N$_{24}$O$_{18}$}$^{3+}$ 1223.3805); 1042.8080 {M$_2$L}$^{2+}$ (calculated for {C$_{107}$H$_{91}$Ir$_2$N$_{13}$O$_9$}$^{2+}$ 1042.8143); FT-IR (cm^{-1}) = 3049, 2926, 1597, 1582, 1499, 1478, 1449, 1415, 1253, 1226, 1188, 1141, 1090, 1048, 1032, 932, 836, 757, 737, 670, 623, 556, 468; UV-Vis λ$_{max}$ (nm) 251 (Intraligand π→π* transitions), 276 (Intraligand π→π* transitions), 295 (Intraligand π→π* transitions), 358 (π→π* *E* isomer), 463 (n→π* *Z* isomer).

1.2 Crystal Structure Determinations

Crystals were mounted under inert oil on a MiTeGen tip and flash frozen to 100(1) or 120(1) K using an OxfordCryosystems low temperature device. X-ray diffraction data were collected using Cu-K_α (λ= 1.54184 Å) or Mo-K_α (λ= 0.71073 Å) radiation using an Agilent Supernova dual-source diffractometer with Atlas S2 CCD detector and fine-focus sealed tube generator, or or using synchrotron radiation (λ= 0.6889 Å) at station I19 of Diamond Light Source. Data were corrected for Lorenztian and polarization effects and absorption corrections were applied. The structures were solved by direct methods using SHELXS-97 and refined by full-matrix on F^2 using SHELXL.[9] Unless otherwise specified, all non-

hydrogen atoms were refined as anisotropic, and hydrogen positions were included at geometrically estimated positions. Summary of data collections and refinements are given in Table S1. Further details of refinements and additional figures are given below.

Table S1. Details of crystal structure data collections and refinements

	**1p·4(H_2O)**	**2p·5(H_2O)**	**L2**	**L3·2(CH_3NO_2)**
CCDC	1859217	1839930	1839931	1859218
Formula	$C_{12}H_{16}N_3NaO_6$	$C_{12}H_{18}N_3NaO_7$	$C_{60}H_{30}N_9O_9$	$C_{65}H_{63}N_{11}O_{13}$
Mr	321.27	339.28	1020.93	1206.26
Crystal colour	yellow	orange	orange	orange
Crystal size (mm)	0.19 x 0.05 x 0.04	0.35 x 0.16 x 0.09	0.33 x 0.17 x 0.10	0.29 x 0.14 x 0.13
Crystal system	Triclinic	Monoclinic	Triclinic	Monoclinic
Space group	$P\bar{1}$	$P2_1/n$	$P\bar{1}$	*I*2/*a*
a (Å)	6.1765(4)	6.5197(6)	9.6864(5)	35.5103(7)
b (Å)	7.0883(4)	35.222(3)	29.786(2)	9.6091(2)
c (Å)	17.5927(6)	7.2310(7)	34.468(4)	37.3391(8)
α (^{0})	88.175(4)	90	71.898(8)	90
β (^{0})	83.629(5)	102.838(10)	88.532(6)	103.589(2)
γ (^{0})	69.716(6)	90	89.954(5)	90
V (Å^3)	717.98(7)	1619.0(3)	9448.9(13)	12384.3(5)
Z	2	4	4	8
ρ_{calc} (g.cm^{-3})	1.486	1.392	0.718	1.294
λ (Å)	1.54184	0.71073	0.6889	1.54184
T (K)	120(1)	120(1)	120(1)	100(1)
θ range (^{0})	5.06-73.97	3.22-28.28	1.53-20.00	3.09-63.83
No. data collected	13098	11758	60957	41057
No. unique data	2761	4026	19323	10085
R_{int}	0.0336	0.0562	0.1479	0.0276
No. obs. Data ($I > 2\sigma(I)$)	2329	3315	5761	7879
No. parameters	263	248	390	804
No. restraints	0	0	17	3
R_1 (obs data)	0.0342	0.0911	0.2253	0.0959
wR_2 (all data)	0.0973	0.1714	0.4885	0.3231
S	1.056	1.259	1.717	1.365

Sodium 4-[2-(4-pyridyl)diazenyl]-benzoate hydrate 1p·4(H_2O)

Fractional coordinates and isotropic displacement parameters for hydrogen atoms of water molecules were freely refined.

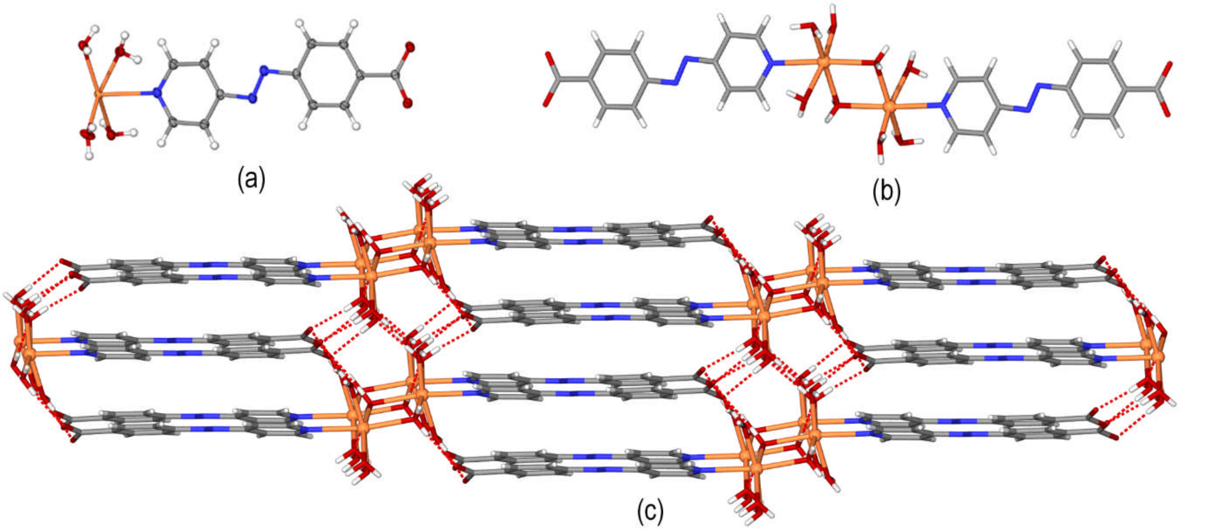

Fig. S1. Crystal structure of **1p**·4(H_2O) where a dinuclear [$Na_2\mu$-$(H_2O)_2(H_2O)_6$(**1p**)$_2$] complex with pyridyl coordination to the Na(I) is formed. (a) Asymmetric unit with ellipsoids shown at 50 % probability levels; (b) dimeric [$Na_2\mu$-$(H_2O)_2(H_2O)_6$(**1p**)$_2$] complex with Na-N distance 2.573(1) Å; (c) section of 3-D hydrogen-bonded network formed by H_2O···H_2O and RCO_2^-···H_2O hydrogen bonds.

Sodium 4-[2-(3-pyridyl)diazenyl]-benzoate hydrate 2p·5(H_2O) Fractional coordinates and isotropic displacement parameters for hydrogen atoms of water molecules were freely refined.

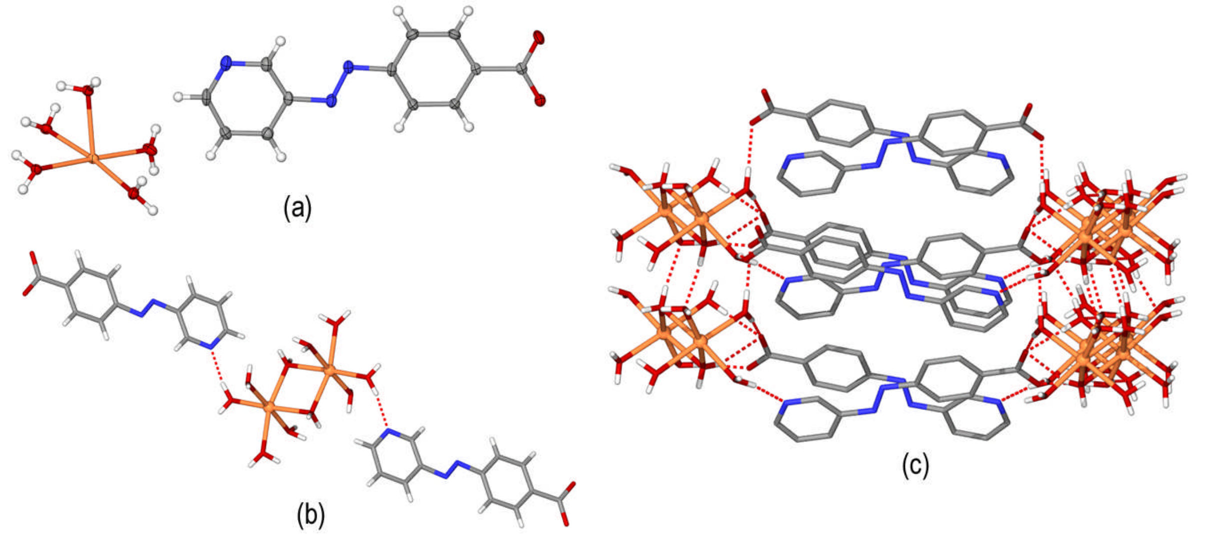

Fig. S2. Crystal structure of **2p**·5(H_2O) where a dinuclear [$Na_2\mu$-$(H_2O)_2(H_2O)_8$]$^{2+}$ cation is formed with 4-[2-(3-pyridyl)diazenyl]-benzoate counter-anions. (a) Asymmetric unit with ellipsoids shown at 50 % probability levels; (b) Hydrogen bonding interactions between [$Na_2\mu$-$(H_2O)_2(H_2O)_8$]$^{2+}$ and pyridyl groups of anion; (c) section of 3-D hydrogen-bonded network formed by H_2O···H_2O, H_2O…N, and RCO_2^-···H_2O hydrogen bonds.

L2 Multiple sets of crystals were examined all of which were of poor quality. Crystals of **L2** diffracted very weakly and did not diffract to high angles even with synchrotron radiation. The high R_{int} of 0.1479 for a triclinic crystal indicates poor crystal quality. The structure was refined isotropically due to poor parameter:observed data ratio. One of the two **L2** molecule in the asymmetric unit showed significant disorder with one azo-phenyl group modelled across two position at 0.65:0.35 occupancies and one azo-pyridine modelled across two positions each at 0.5 occupancy. Half of the aromatic ring groups of the structure were refine with a rigid body constraint (AFIX 66) and some additional restraints were placed on azo N-N and C-N distances (DFIX). Two pyridyl groups were refined each with one group displacement parameter. There was significant solvent accessible void space in the structure (48 % of unit cell volume) and the SQUEEZE routine of Platon was used.[10] Additional electron density was not added to the formula. The structure should be regarded as showing the gross structural features, and positions of pyridyl groups and assignment of N atom assignment pyridyl groups should be regarded as approximate.

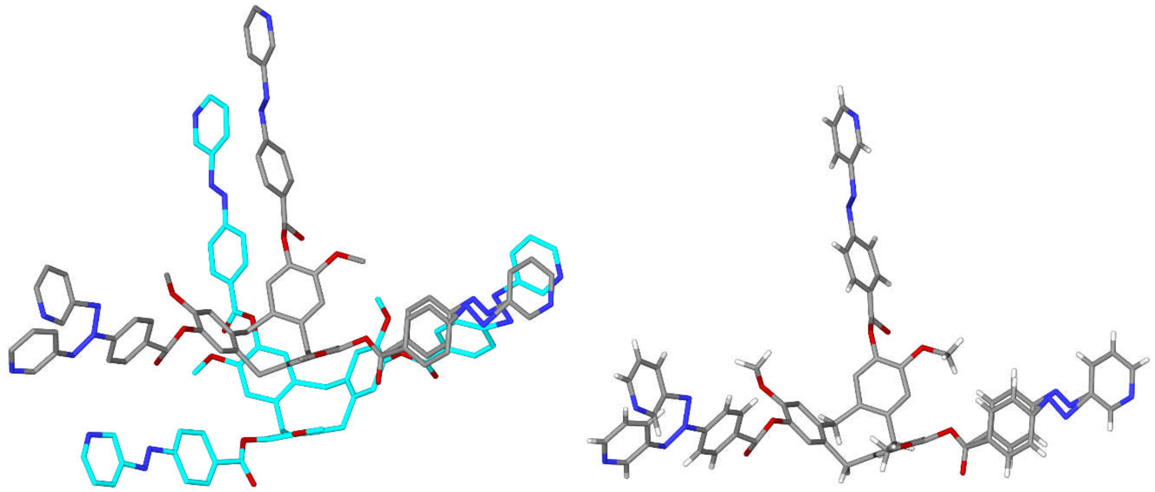

Fig. S3. Crystal structure of **L2** showing asymmetric unit (left) and highlight of disorder in one ligand (right).

L3·2($MeNO_2$) Two $MeNO_2$ positions within **L3**·2($MeNO_2$) were each refined isotropically at 0.5 occupancy. Restraints were used for $MeNO_2$ bond lengths and a FLAT restraint was employed for one such molecule.

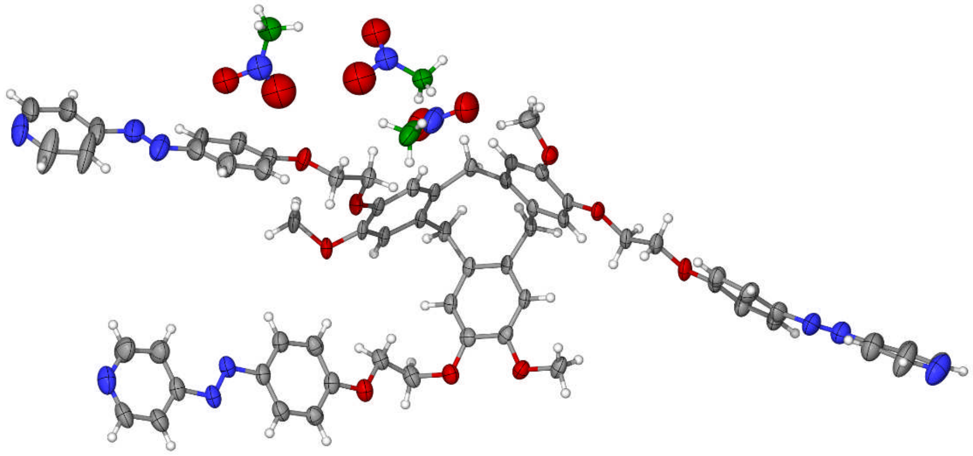

Fig. S4. Asymmetric unit of **L3**·2($MeNO_2$) with ellipsoids shown at 50 % probability levels.

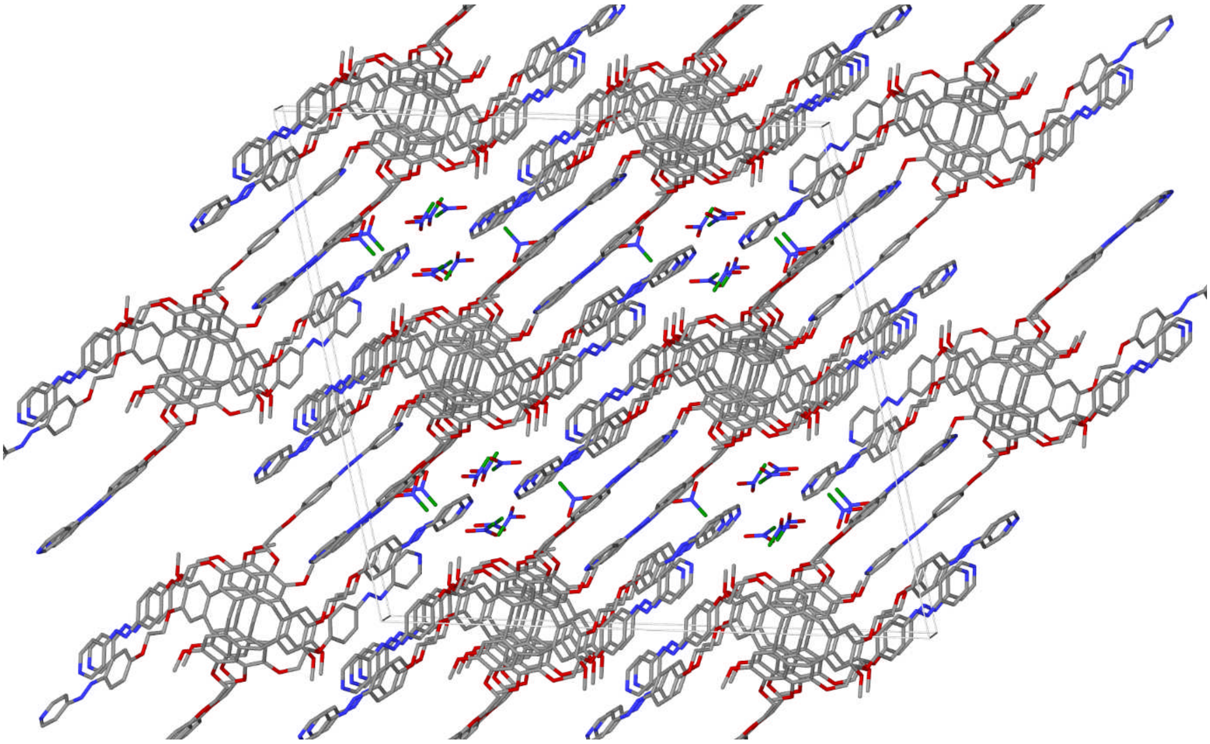

Fig. S5. Packing diagram of **L3**·2(MeNO$_2$) viewed down *b* axis.

1.3 Molecular Modelling

Molecular models were constructed using the Maestro 2016-3 molecular modelling Package (Schrodinger, LLC, New York, NY, 2016). Models of the photoswitchable cages were constructed initially 'by eye' using the 'build' module within Maestro to modify the available crystal structure of the **L2** ligand together with structures corresponding to the metal-ppy units. Following 'by eye' assembly of these modified units to yield a crude model of the appropriate cage, the resulting structure was energy minimised using the gradient minimiser (MMFF force-field) and the default parameters within the programme.

1.4 Photoswitching Experiments

UV-Visible spectra were recorded on a Lambda 900 UV/Vis spectrophotometer and an Agilent Cary 100 UV/Vis spectrophotometer. *E*→*Z* photoswitching experiments were performed with a Xe lamp for ligands and more powerful laser for some ligands and all coordination cages (as cages showed only small amount of switching with Xe lamp), while all *Z*→*E* isomerisations were performed using the Xe lamp.

Isomerisation of ligands with Xe lamp

A 75W Xe lamp equipped with a tunable PowerArc monochromator was used. Note the majority of this 75W is filtered out at wavelengths <400 nm, and wattage at < 400 nm is <1 milliwatt of energy.

E→*Z* isomerisation: A 30 μM solution of the appropriate ligand in DMSO was prepared and the UV spectrum recorded. The sample was irradiated at λ_1 (300-350 nm) for 45 minutes using the Xenon lamp equipped with a tunable power arc monochromator to induce *E*→*Z* isomerisation. The UV spectrum of this solution was then recorded for comparison. *Z*→*E* isomerisation: The solution was irradiated at λ_2 (450 nm) for 15 minutes using the Xenon lamp equipped with a tuneable power arc monochromator to induce *Z*→*E* isomerisation. The UV spectrum of this solution was then recorded for comparison.

Isomerisation of ligands and metallo-cages with 355 nm Nd:YAG laser

A Continuum Powerlite model 8010 355 nm Nd:YAG laser was used which has a natural frequency of 10 Hz. For experiments at 1Hz the natural frequency was split after 200 microseconds using a Q-switch in pulse divide mode set at 1Hz.

E (trans) → Z (*cis)* isomerisation metallo-cage for UV studies: 30 µM solutions of each metallo-cage were prepared in DCM and the UV spectrum recorded. The sample was irradiated at 355 nm using a Nd:YAG laser (1Hz, 15mJ/laser pulse) and UV spectra were recorded at intervals.

E→*Z* isomerisation of metallo-cage **C1-F** and ligands **L2/L4** for NMR studies: 5 mg of material was dissolved in in CD_2Cl_2 (0.5 ml). The sample was irradiated at 355 nm using a Nd:YAG laser (10Hz, 15mJ/laser pulse) for 900 seconds.

The efficiency of isomerisation between *E* and *Z* isomers was calculated either through ^{1}H NMR integration or absorption maxima in UV-visible spectra according to the equation:

$$Conversion = \frac{A_{0(\max)} - A_{PSS\,(max)}}{A_{0(\max)}}$$

where $A_{0\,(max)}$ is the initial absorbance at the maxima of interest and $A_{PSS\,(max)}$ is the absorbance of the maxima at the photostationary/switched state after irradiation.

Reverse Z → E switching of metallo-cages was achieved though irradiated at λ_2 (450 nm) for 15 minutes using the Xenon lamp as described for ligands above.

1.5 Emission Studies

All samples were prepared in HPLC grade dichloromethane with varying concentrations in the order of $10^{-4} – 10^{-6}$ M. Absorption spectra were recorded at room temperature using a Shimadzu UV-1800 double beam spectrophotometer. Molar absorptivity determination was verified by linear least-squares fit of values obtained from at least four independent solutions at varying concentrations with absorbance ranging from 6.05×10^{-5} to 2.07×10^{-5} M.

The sample solutions for the emission spectra were prepared in HPLC-grade DCM and degassed *via* freeze-pump-thaw cycles using a quartz cuvette designed in-house. Steady-state emission and excitation spectra and time-resolved emission spectra were recorded at 298 K using an Edinburgh Instruments F980. All samples for steady-state measurements were excited at 360 nm, while samples for time-resolved measurements were excited at 378 nm using a PDL 800-D pulsed diode laser. Emission quantum yields were determined using the optically dilute method.[11] A stock solution with absorbance of *ca*. 0.5 was prepared and then four dilutions were prepared with dilution factors between 2 and 20 to obtain solutions with absorbances of *ca*. 0.095 0.065, 0.05 and 0.018, respectively. The Beer-Lambert law was found to be linear at the concentrations of these solutions. The emission spectra were then measured after the solutions were rigorously degassed *via* three freeze-pump-thaw cycles prior to spectrum acquisition. For each sample, linearity between absorption and emission intensity was verified through linear regression analysis and additional measurements were acquired until the Pearson regression factor (R^2) for the linear fit of the data set surpassed 0.9. Individual relative quantum yield values were calculated for each solution and the values reported represent the slope value. The equation $\Phi_s = \Phi_r(A_r/A_s)(I_s/I_r)(n_s/n_r)^2$ was used to calculate the relative quantum yield of each of the sample, where Φ_r is the absolute quantum yield of the reference, *n* is the refractive index of the solvent, *A* is the absorbance at the excitation wavelength, and *I* is the integrated area under the corrected emission curve. The subscripts s and r refer to the sample and reference, respectively. A solution of quinine sulfate in 0.5 M H_2SO_4 (Φ_r = 54.6%)[12] was used as external references.[13]

PMMA doped films were prepared by spin coating the samples from a solution of 2-methoxyethanol (HPLC grade) containing 5 % w/w of the desired sample. Steady-state emission and excitation spectra and time-resolved emission spectra of both powders and doped films were recorded at 298 K using an Edinburgh Instruments F980. Solid-state PLQY measurements of thin films were performed in an integrating sphere under a nitrogen purge in a Hamamatsu C9920-02 luminescence measurement system.[14]

2. Spectra supporting synthesis

Sodium 4-[2-(4-pyridyl)diazenyl]-benzoate (1p)

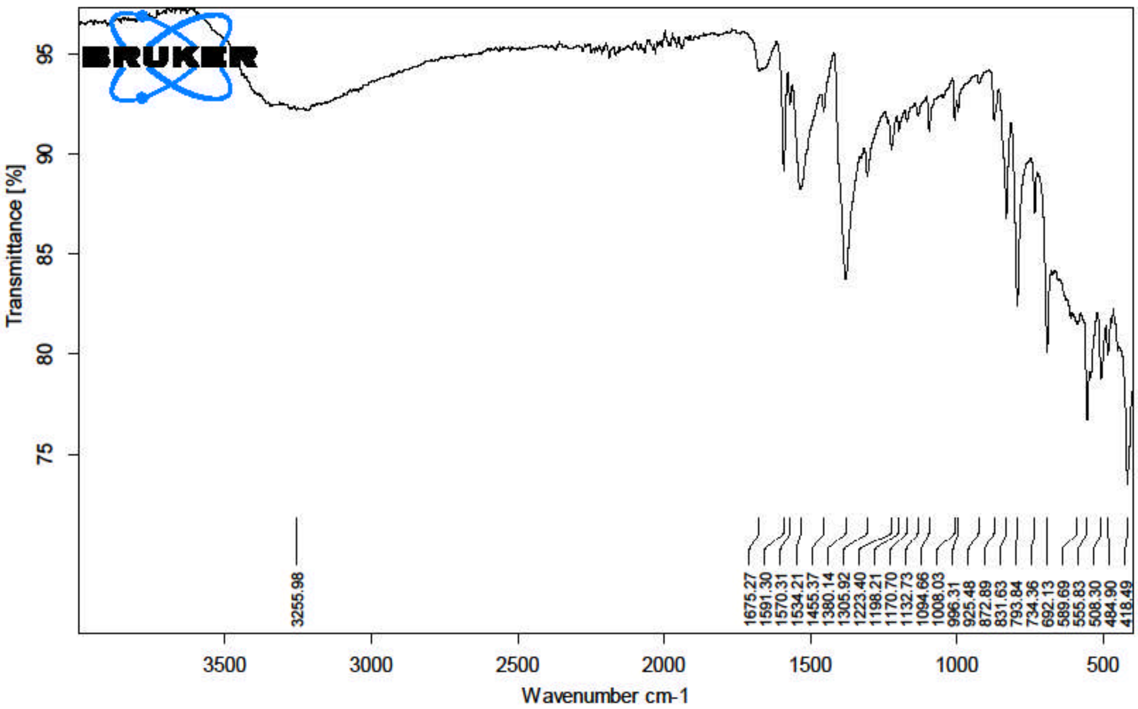

Fig. S6 Infrared spectrum of sodium 4-[2-(4-pyridyl)diazenyl]-benzoate

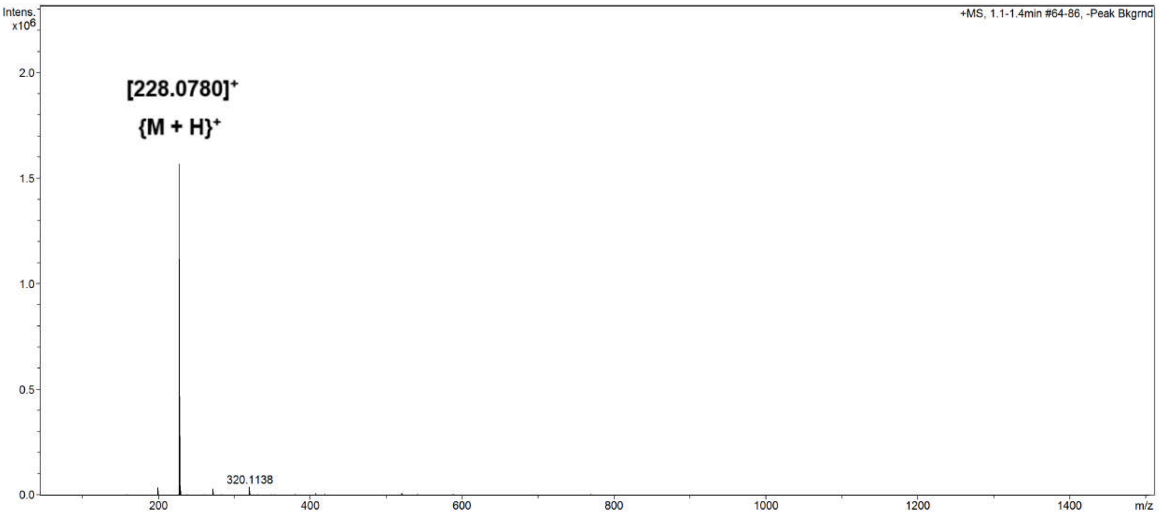

Fig. S7 Mass spectrum of sodium 4-[2-(4-pyridyl)diazenyl]-benzoate

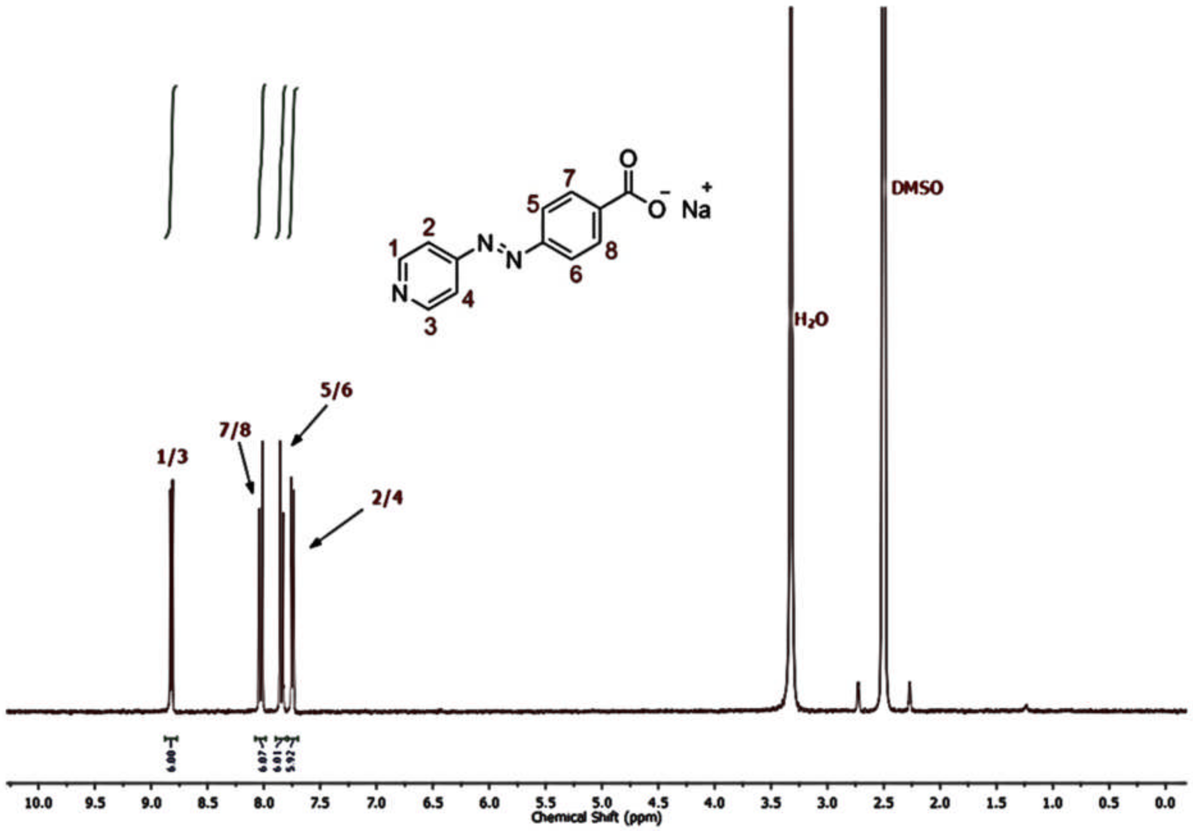

Fig. S8 ^{1}H NMR (300 MHz, DMSO-d^6) of sodium 4-[2-(4-pyridyl)diazenyl]-benzoate

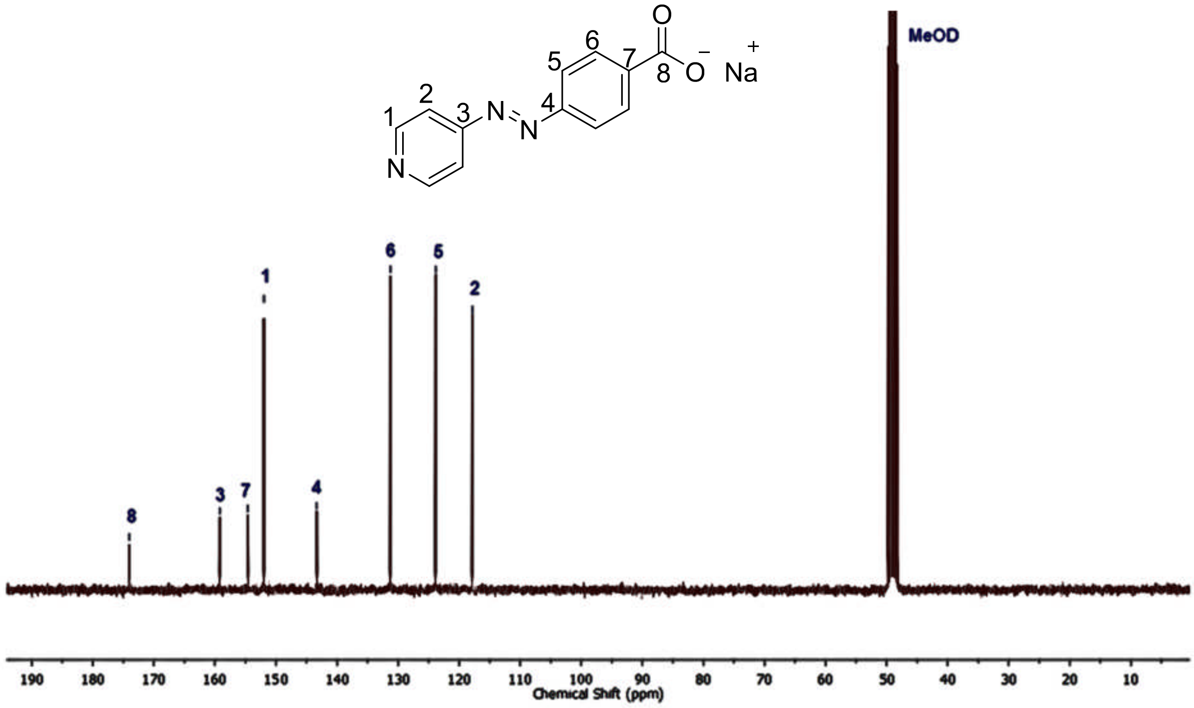

Fig. S9: ^{13}C NMR (75 MHz, MeOD-d^4) of sodium 4-[2-(4-pyridyl)diazenyl]-benzoate

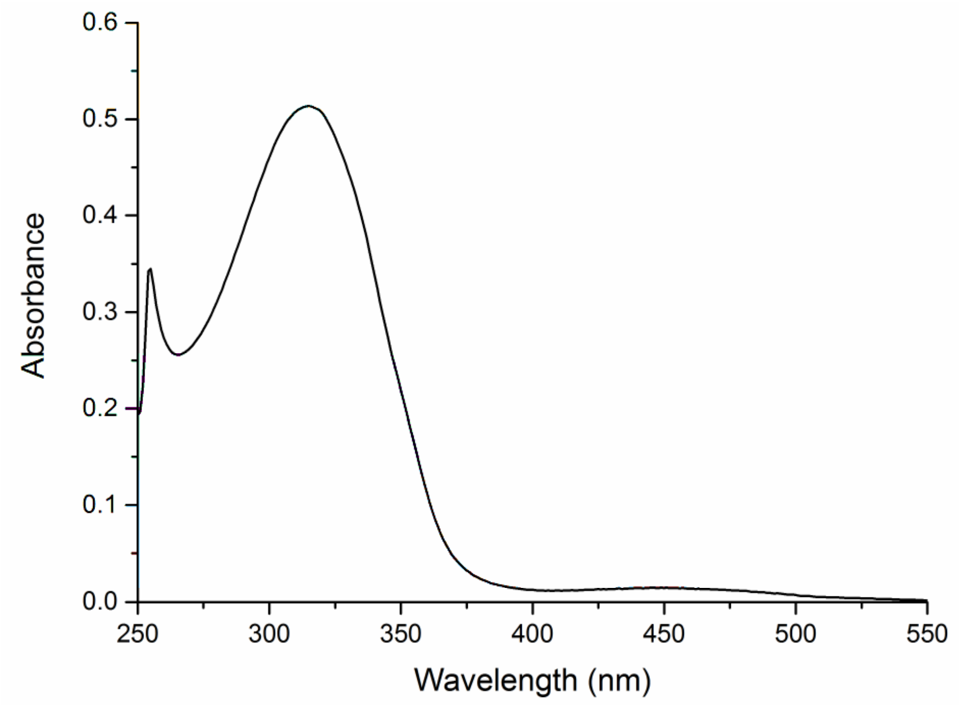

Fig. S10 UV-visible spectrum of sodium 4-[2-(4-pyridyl)diazenyl]-benzoate in DMSO

Sodium 4-[2-(3-pyridyl)diazenyl]-benzoate (2p)

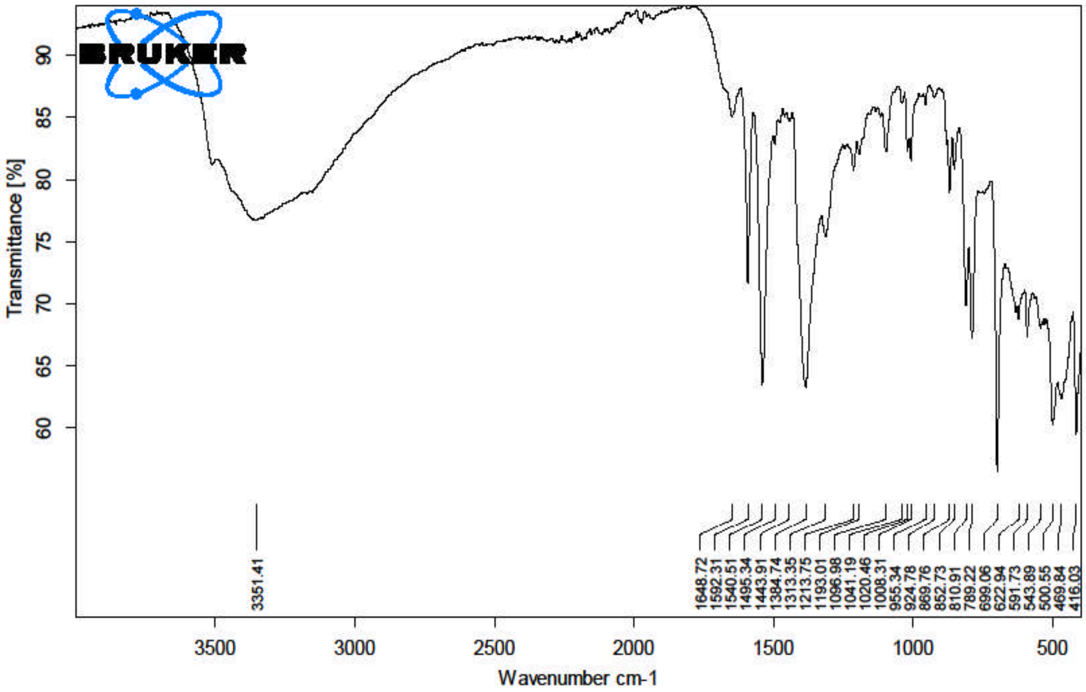

Fig. S11 Infrared spectrum of sodium 4-[2-(3-pyridyl)diazenyl]-benzoate

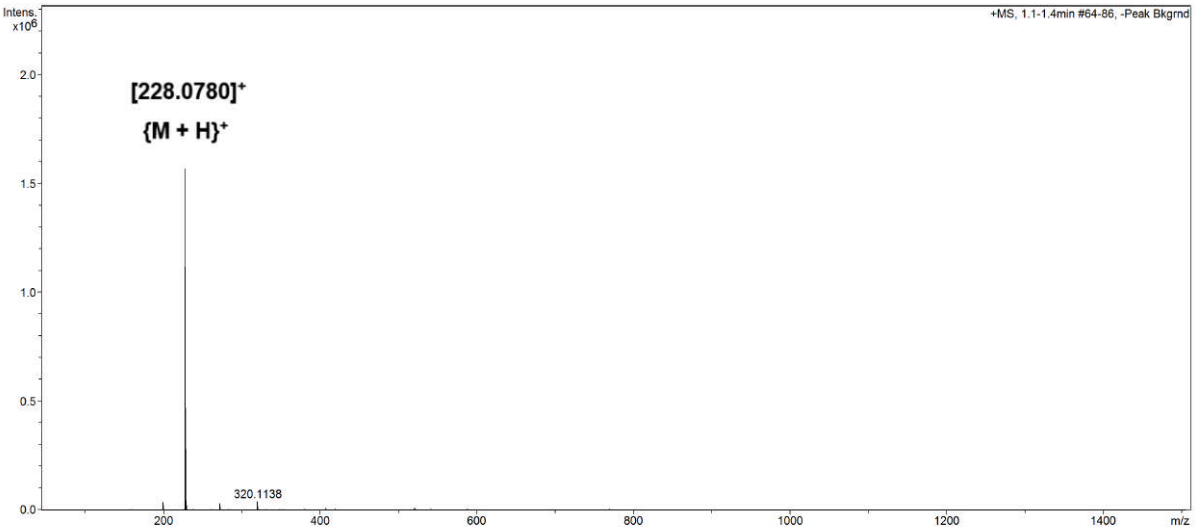

Fig. S12 Mass spectrum of sodium 4-[2-(3-pyridyl)diazenyl]-benzoate

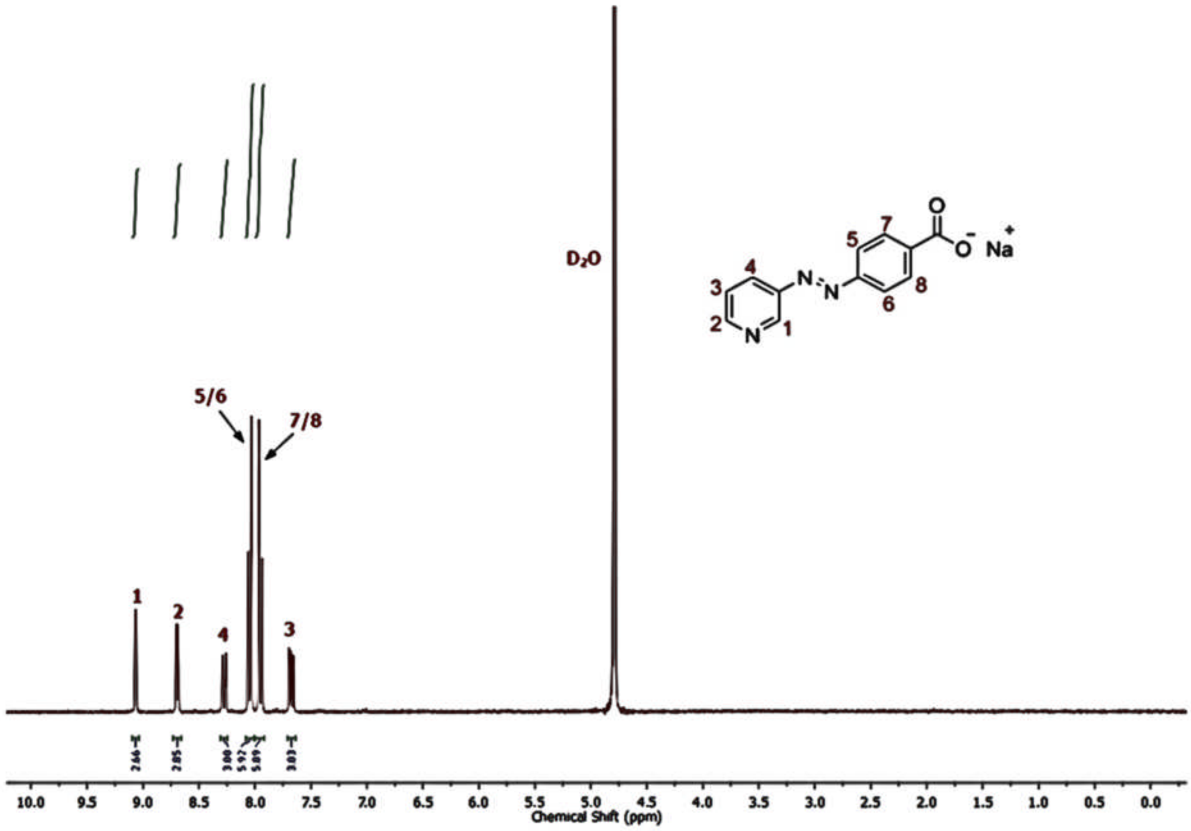

Fig. S13 ^{1}H NMR (300 MHz, D_2O) of sodium 4-[2-(3-pyridyl)diazenyl]-benzoate

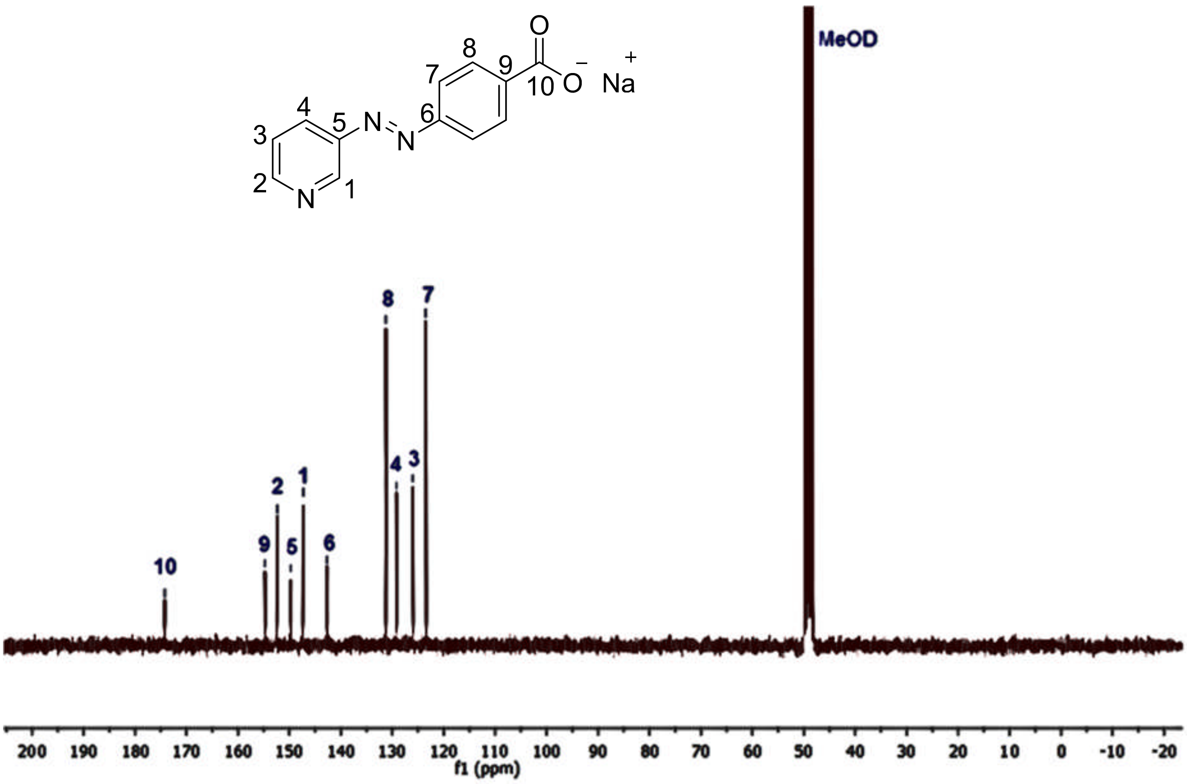

Fig. S14: ^{13}C NMR (75 MHz, MeOD-d^4) of sodium 4-[2-(3-pyridyl)diazenyl]-benzoate

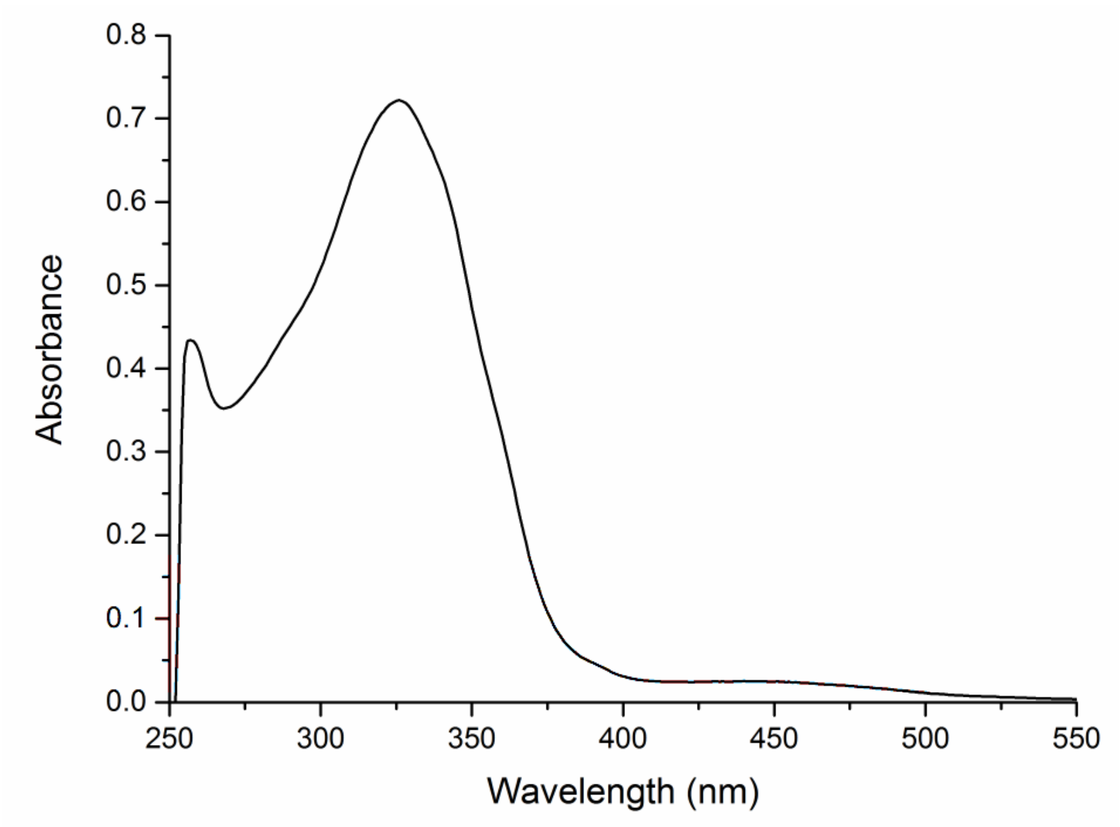

Fig. S15 UV-visible spectrum of sodium 4-[2-(3-pyridyl)diazenyl]-benzoate in DMSO

(±)-2,7,12-Trimethoxy-3,8,13-*tris*(4,4’-pyridyl-azophenylcarboxy)-10,15-dihydro-*5H*-tribenzo[*a,d,g*]cyclononene (L1)

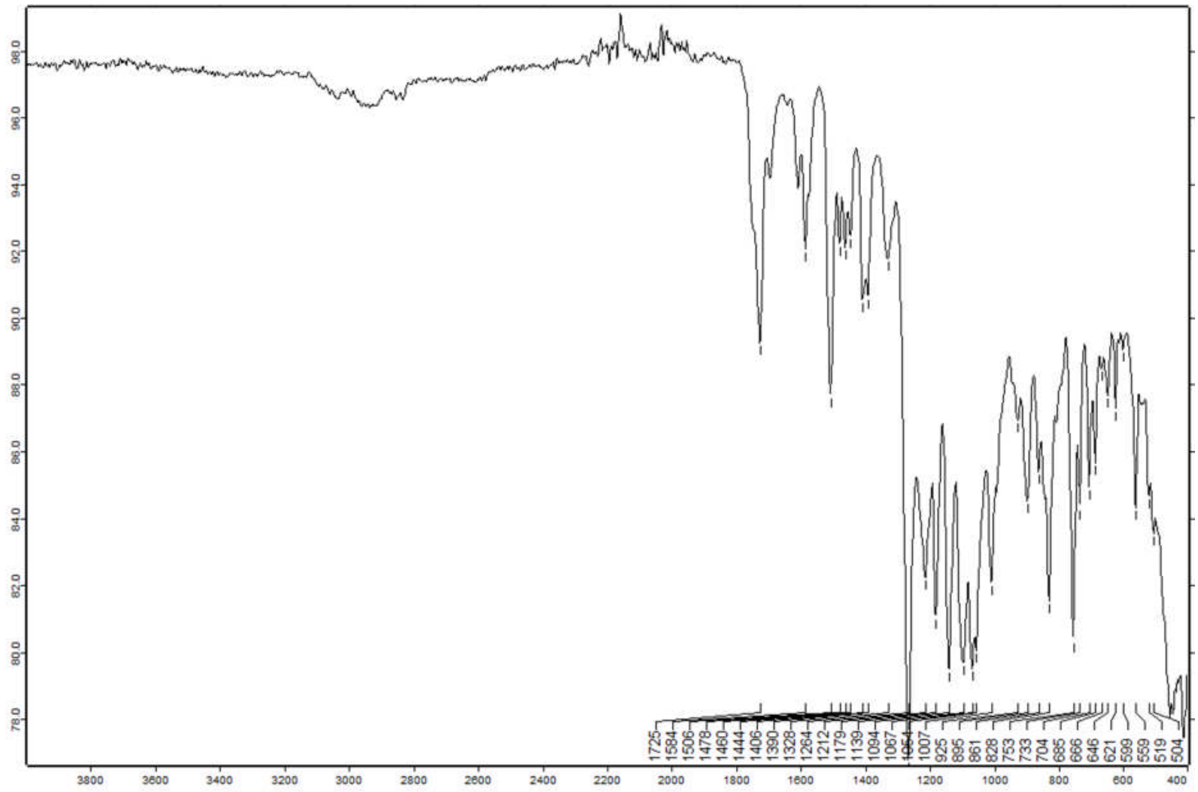

Fig. S16 Infrared spectrum of L1

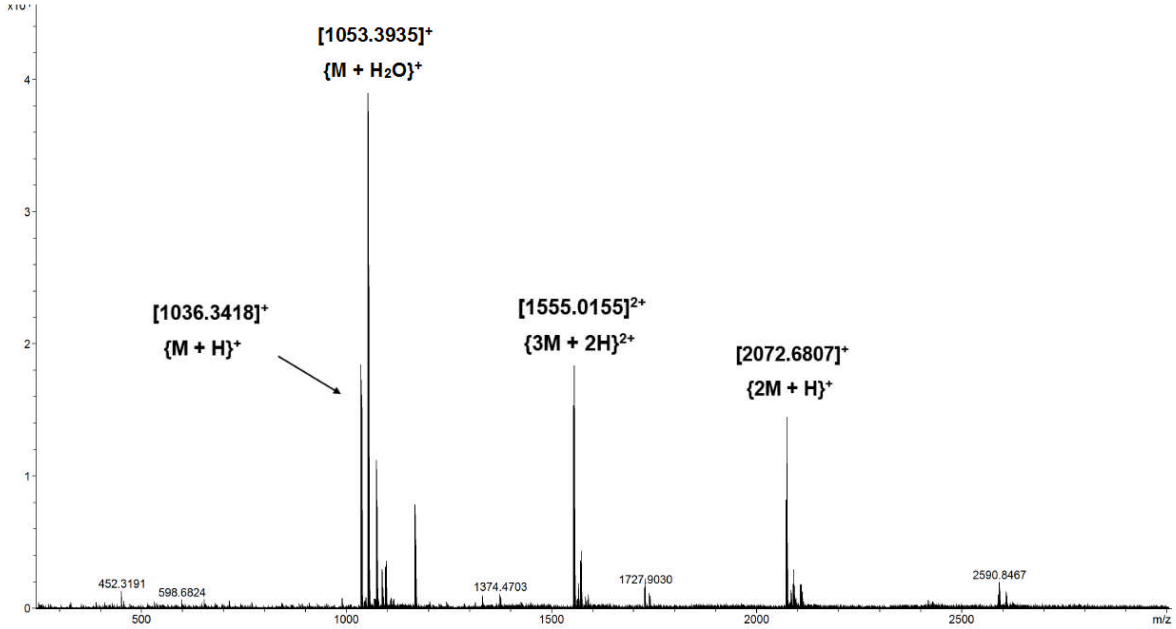

Fig. S17 Mass spectrum of **L1**.

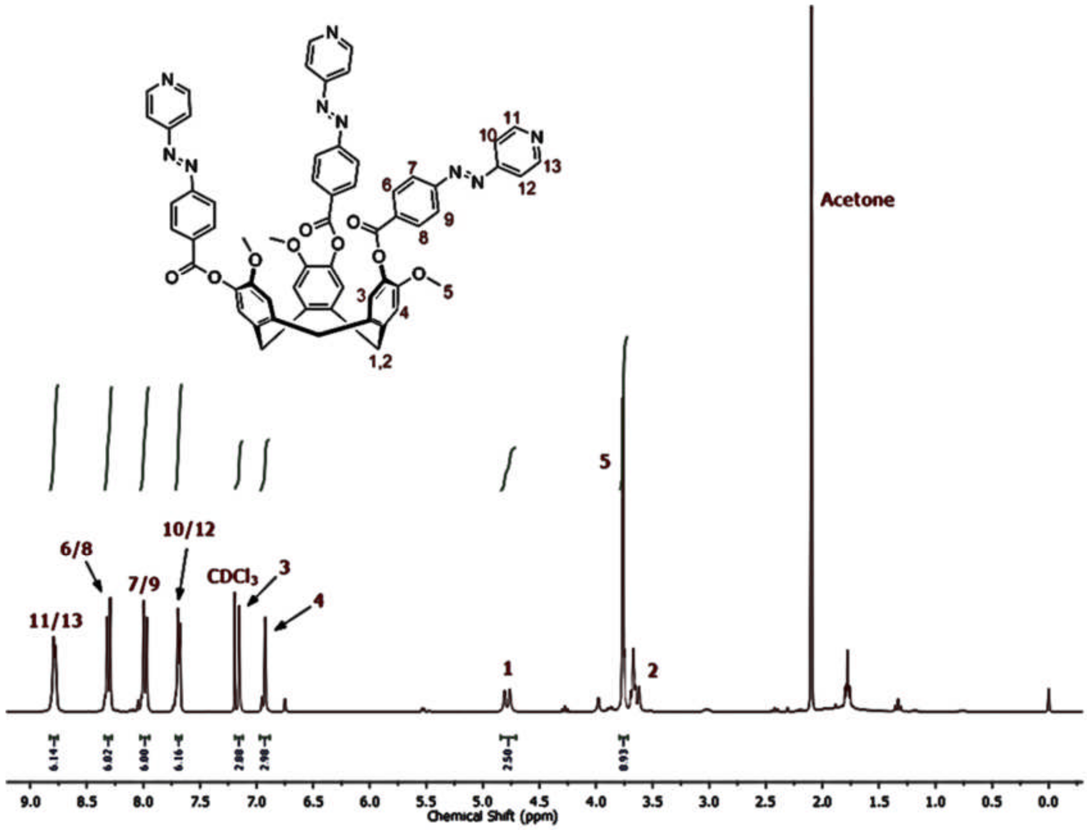

Fig. S18 ^{1}H NMR (300 MHz, DMSO-d^{6}) of **L1**

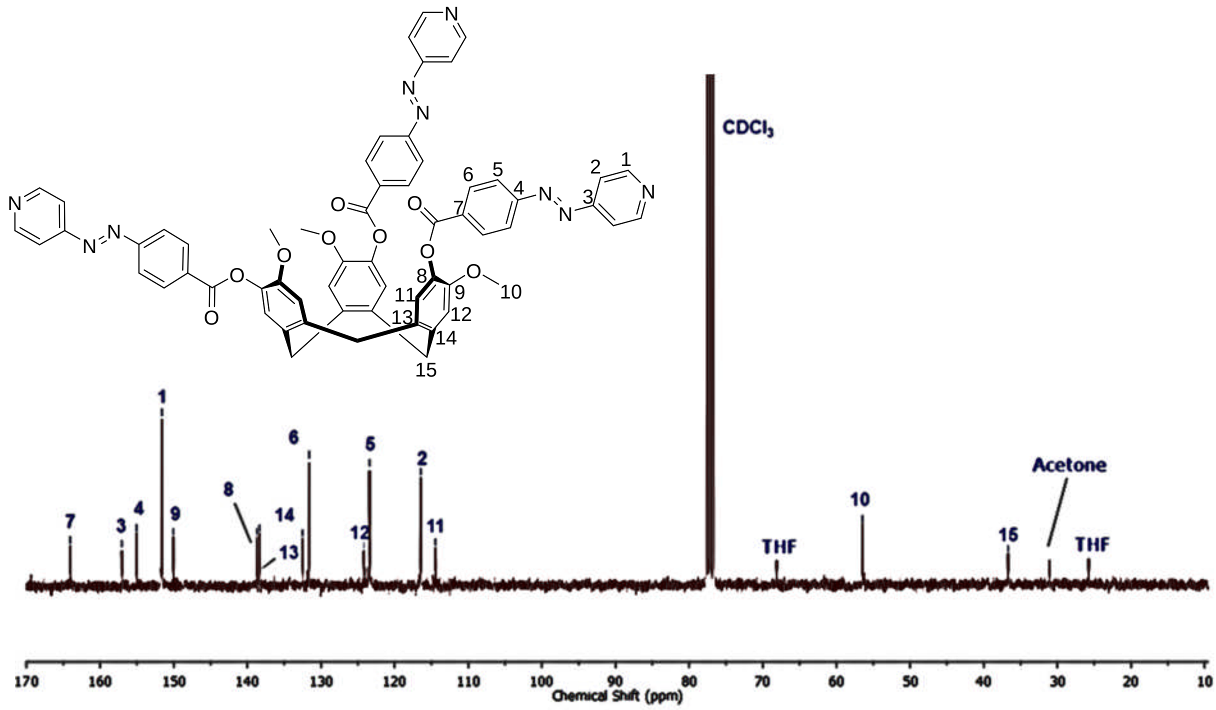

Fig. S19 C^{13} NMR (75 MHz, $CDCl_3$) of **L1**

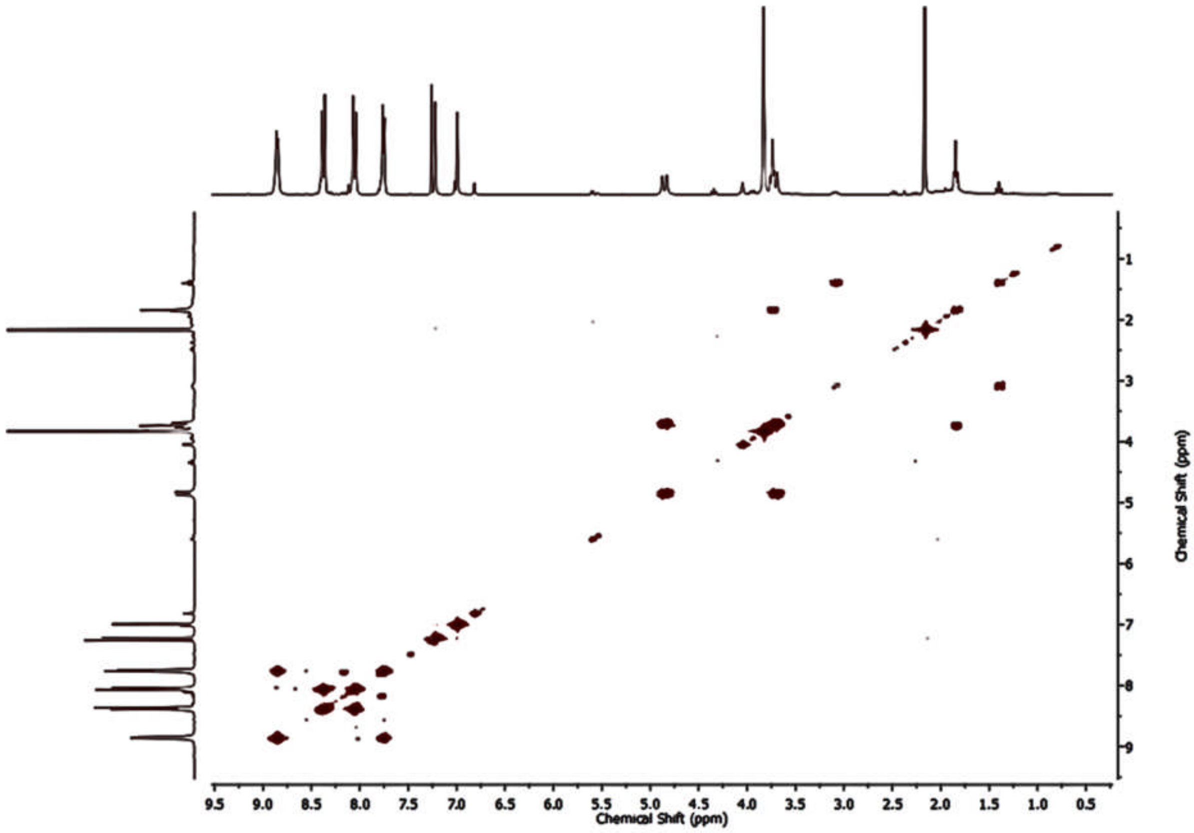

Fig. S20 ^{1}H-^{1}H COSY NMR (400 MHz, DMSO-d^{6}) of **L1**

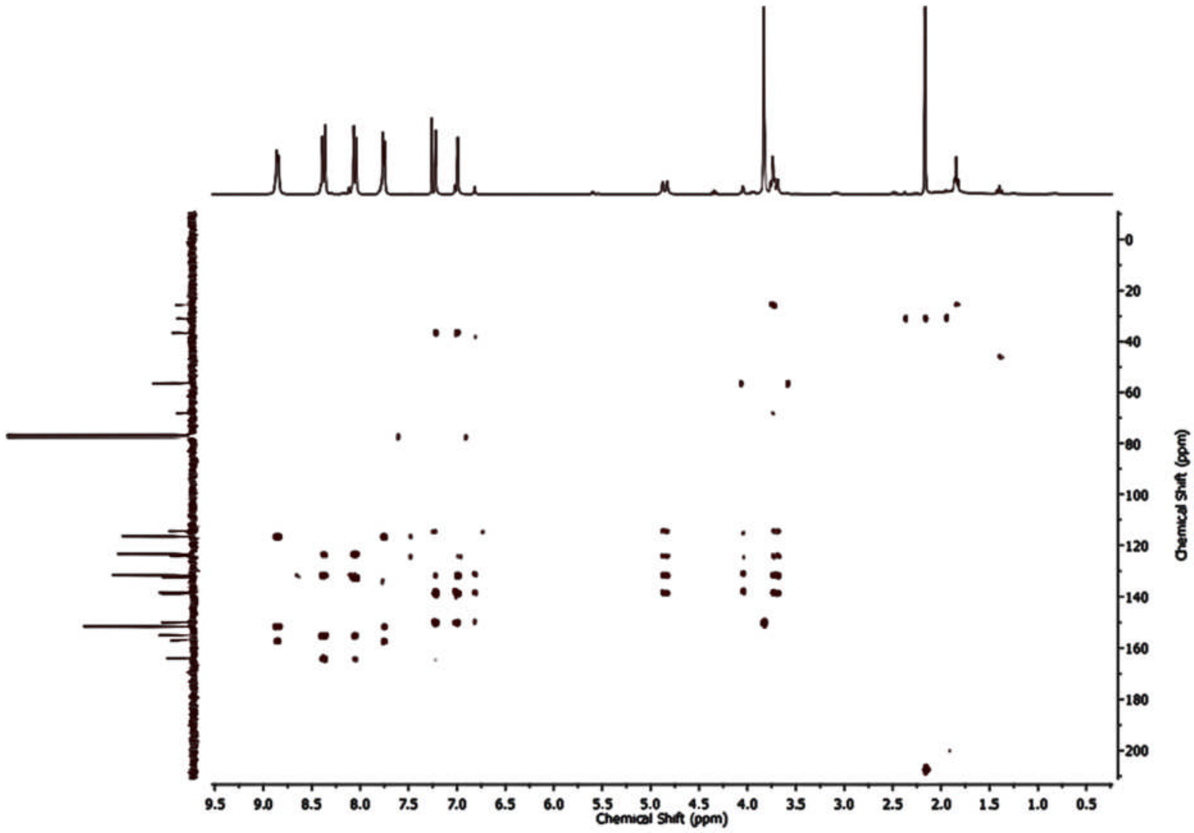

Fig. S21 ^{1}H-^{13}C HMBC NMR (400 MHz, DMSO-d^{6}) of L1

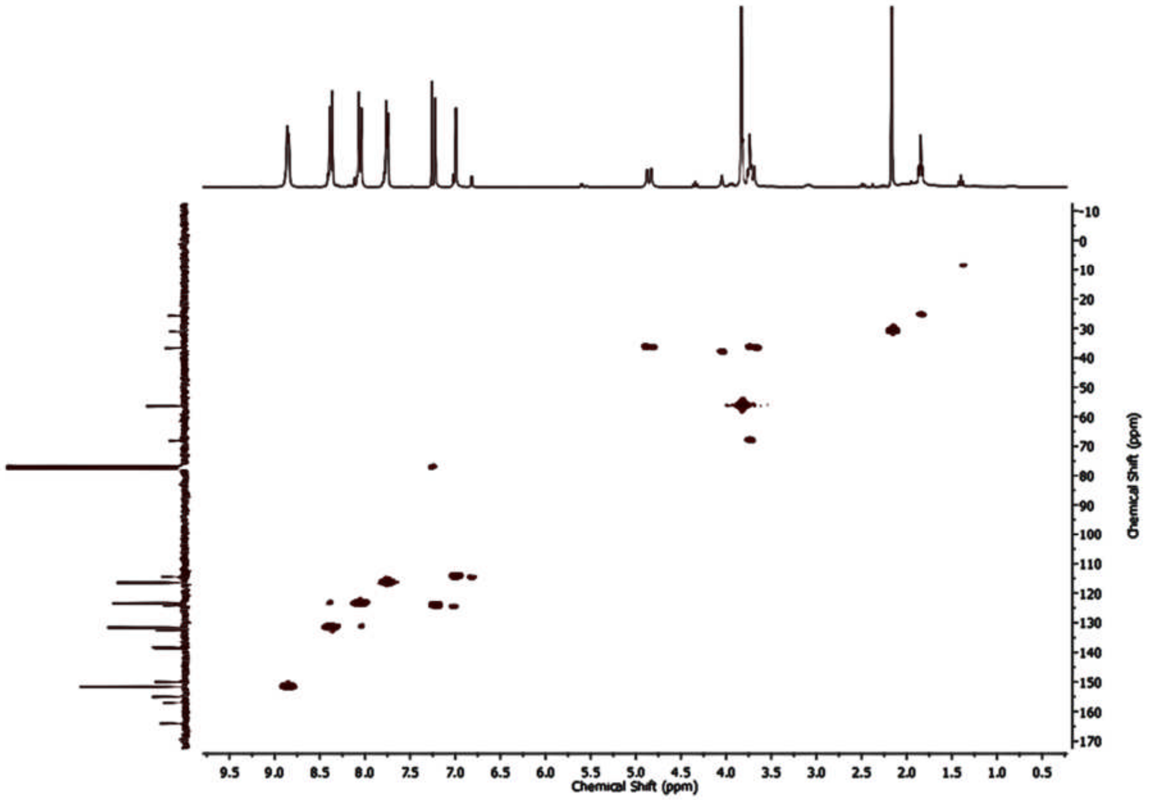

Fig. S22 ^{1}H-^{13}C HMQC NMR (400 MHz, DMSO-d^6) of **L1**

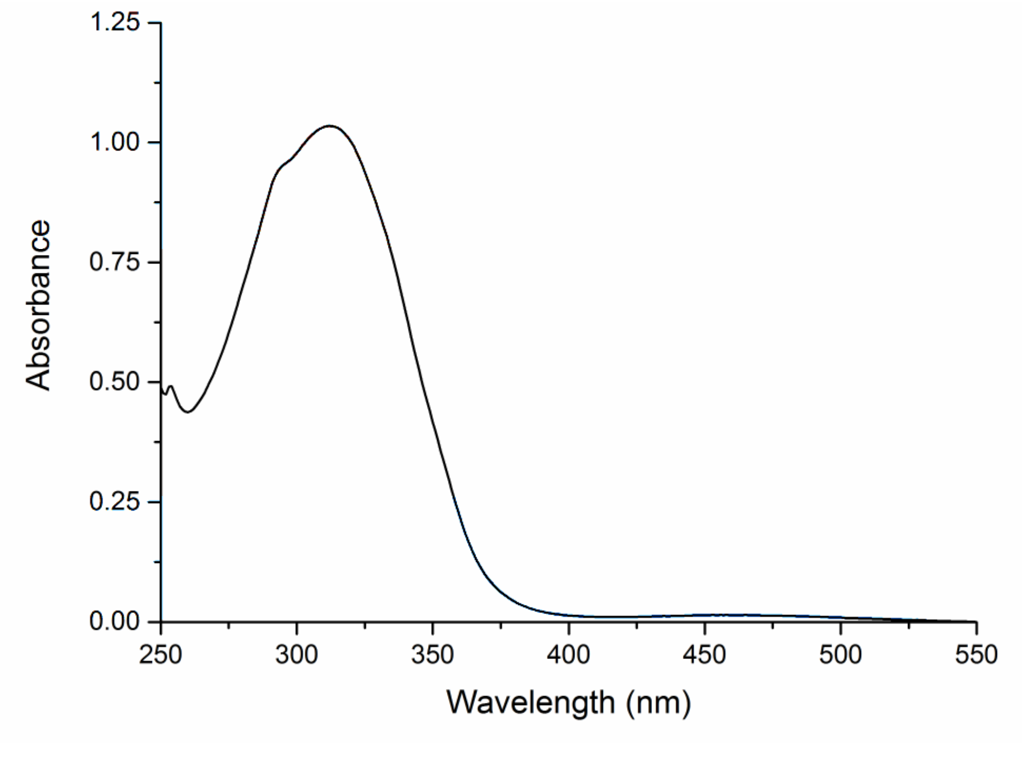

Fig. S23 UV-visible spectrum of **L1** in DMSO.

(±)-2,7,12-Trimethoxy-3,8,13-*tris*(4,3'-pyridyl-azophenylcarboxy)-10,15-dihydro-*5H*-tribenzo[*a,d,g*]cyclononene (L2)

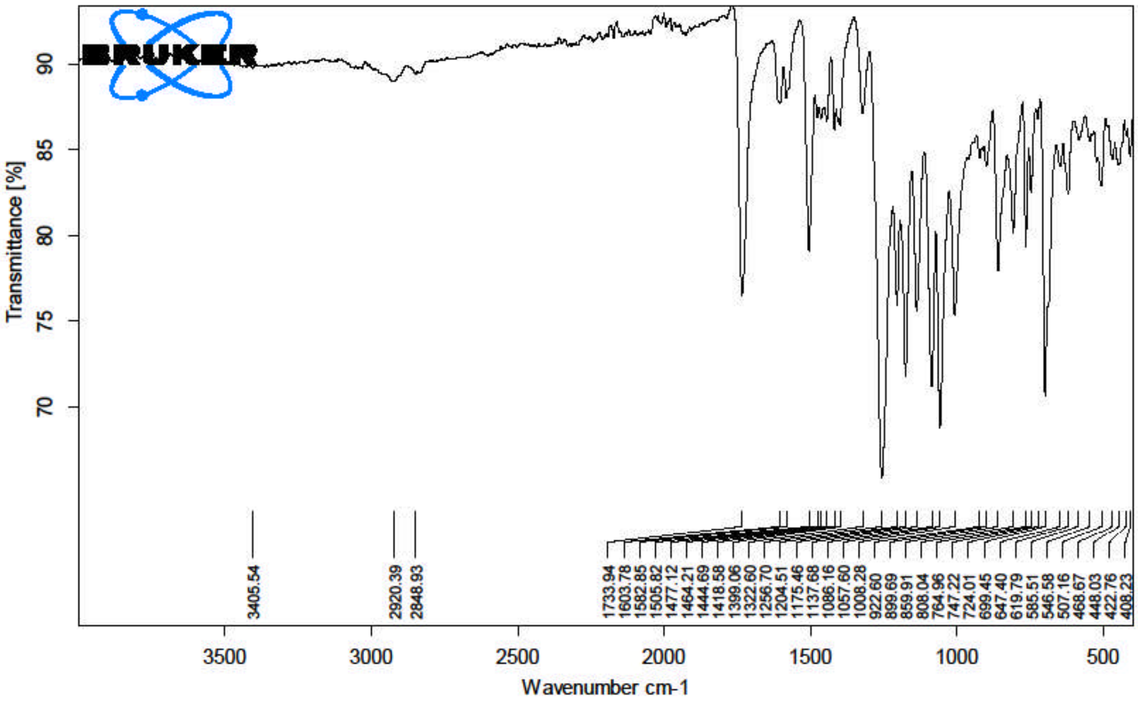

Fig. S24 Infrared spectrum of **L2**.

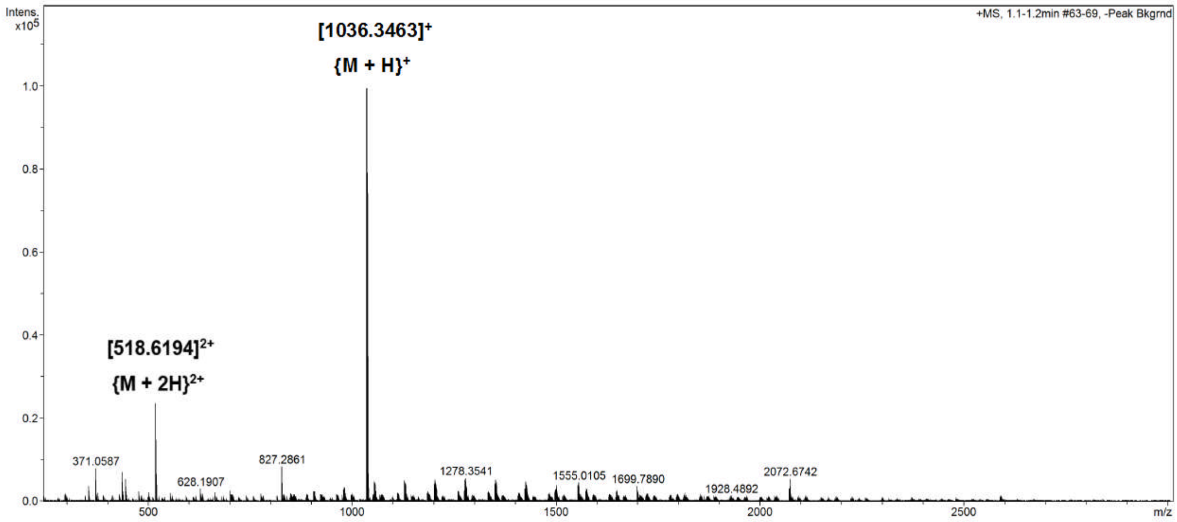

Fig. S25 Mass spectrum of **L2**.

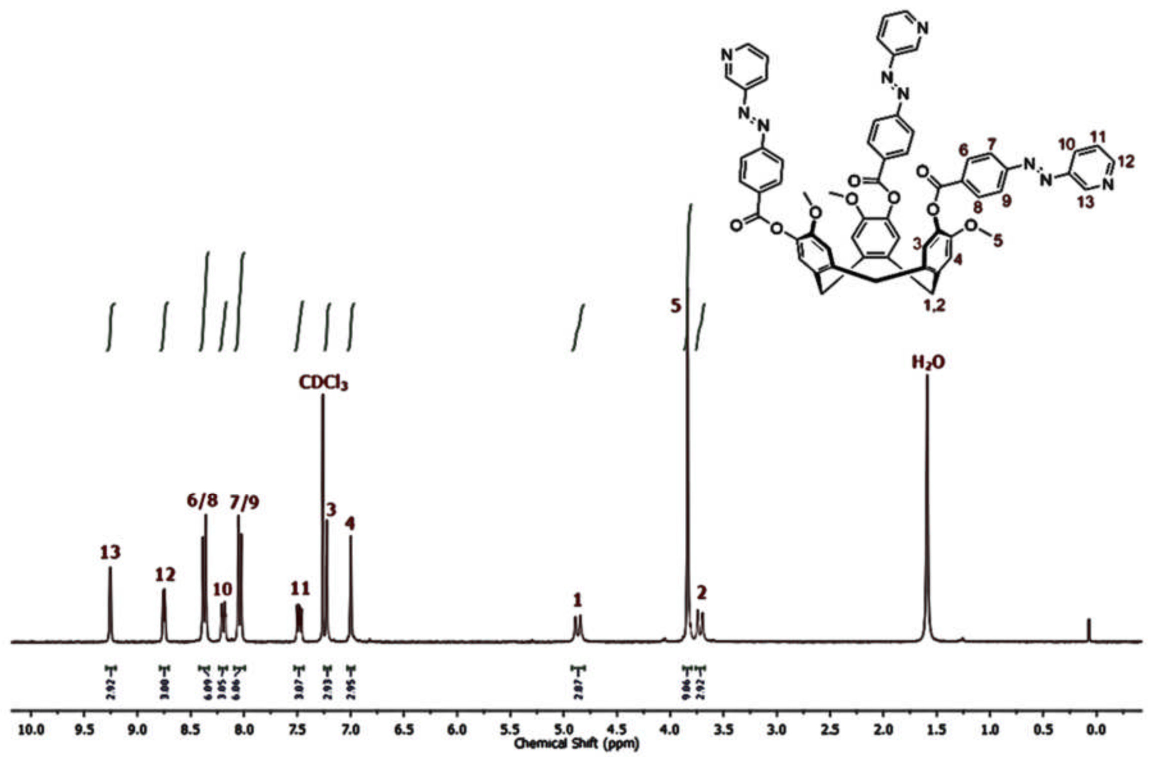

Fig. S26 ^{1}H NMR (300 MHz, $CDCl_3$) of **L2**.

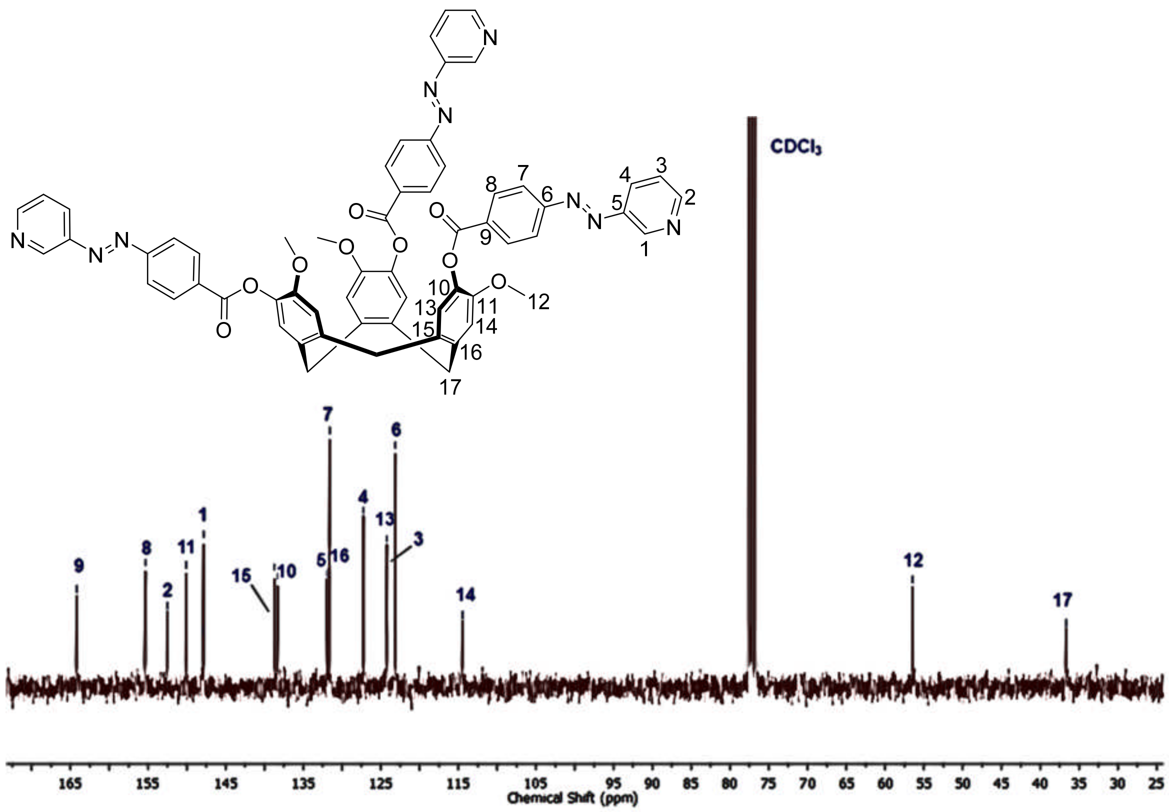

Fig. S27. ^{13}C NMR (75 MHz, $CDCl_3$) of **L2**.

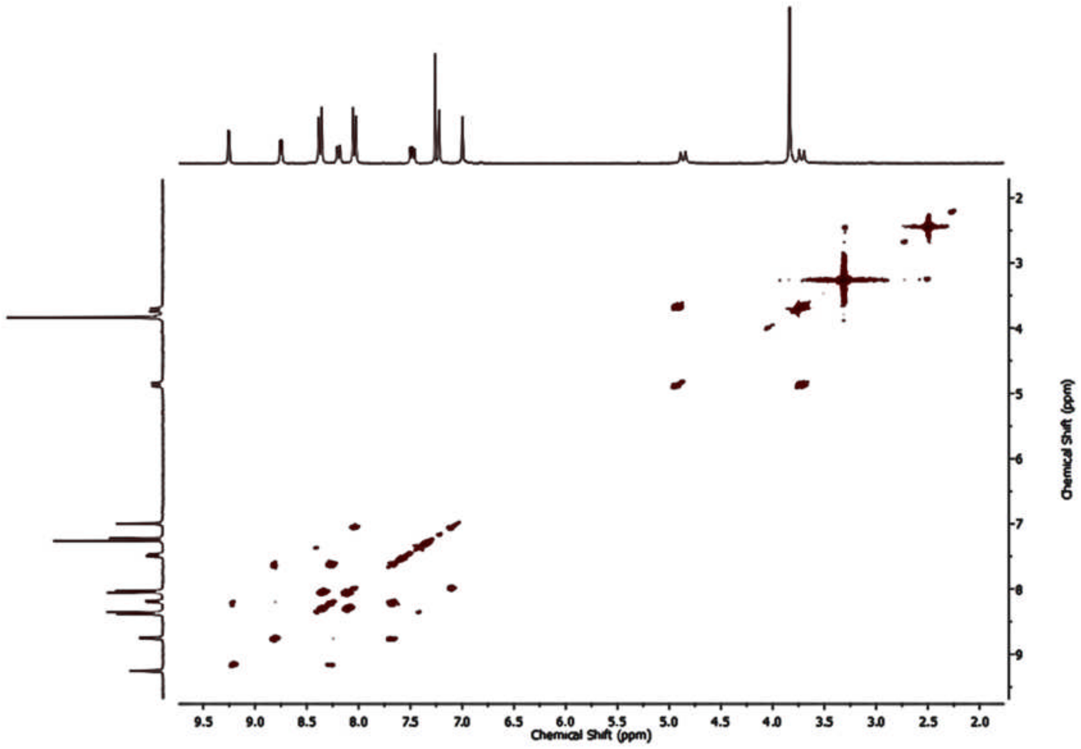

Fig. S28. ^{1}H-^{1}H COSY NMR (400 MHz, $CDCl_3$) of **L2**.

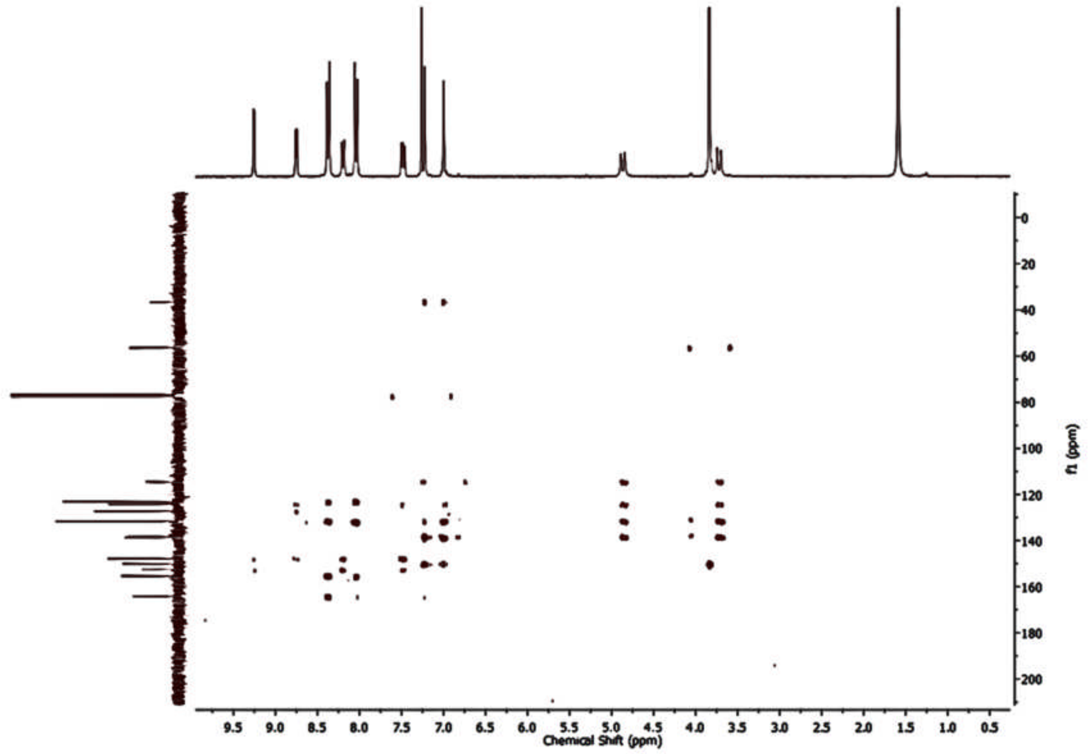

Fig. S29. ^{1}H-^{13}C HMBC NMR (400 MHz, $CDCl_3$) of **L2**.

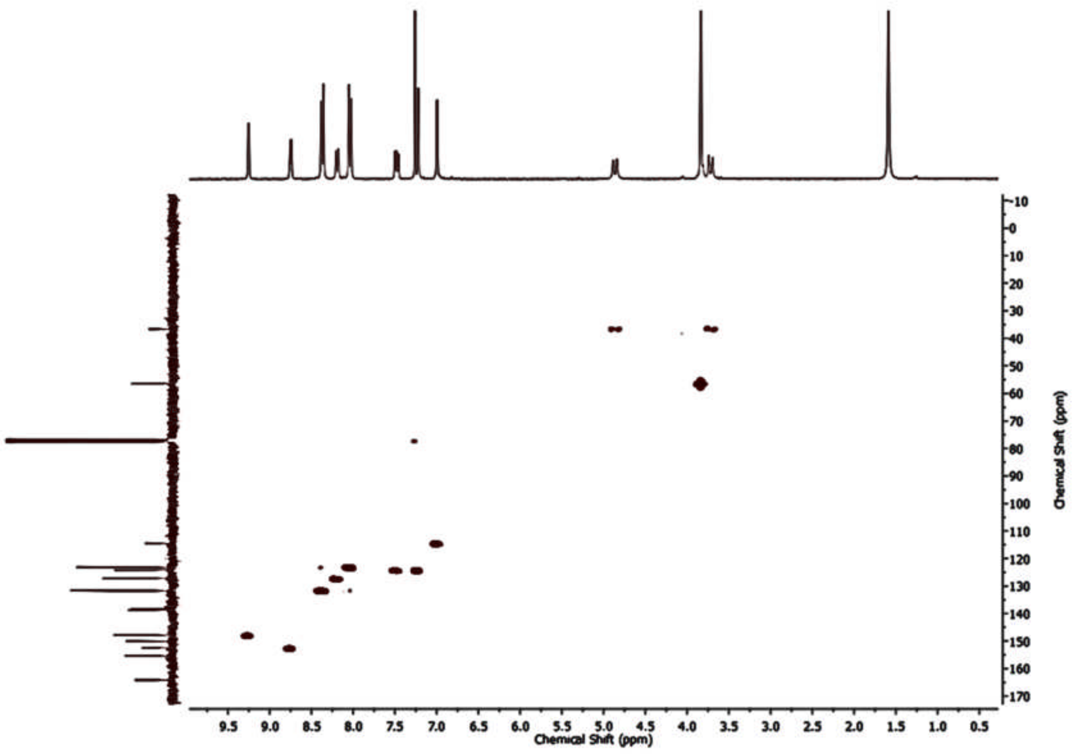

Fig. S30 ^{1}H-^{13}C HMQC NMR (400 MHz, $CDCl_3$) of **L2**.

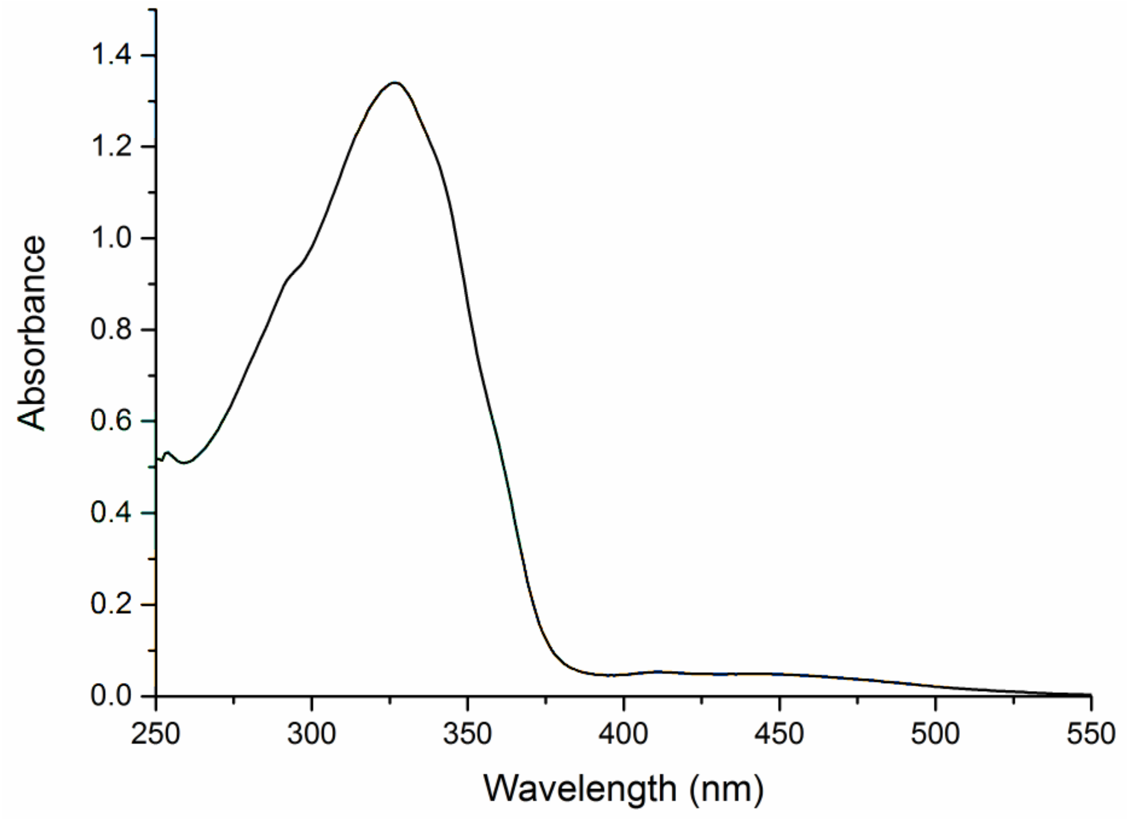

Fig. S31. UV-visible spectrum of **L2** in DMSO

2,7,12-Tris-(2-(4-pyridylazo)ethoxy)-3,8,13-trimethoxy-10,15-dihydro-2H-tribenzo[*a*,*d*,*g*]cyclononene (L3)

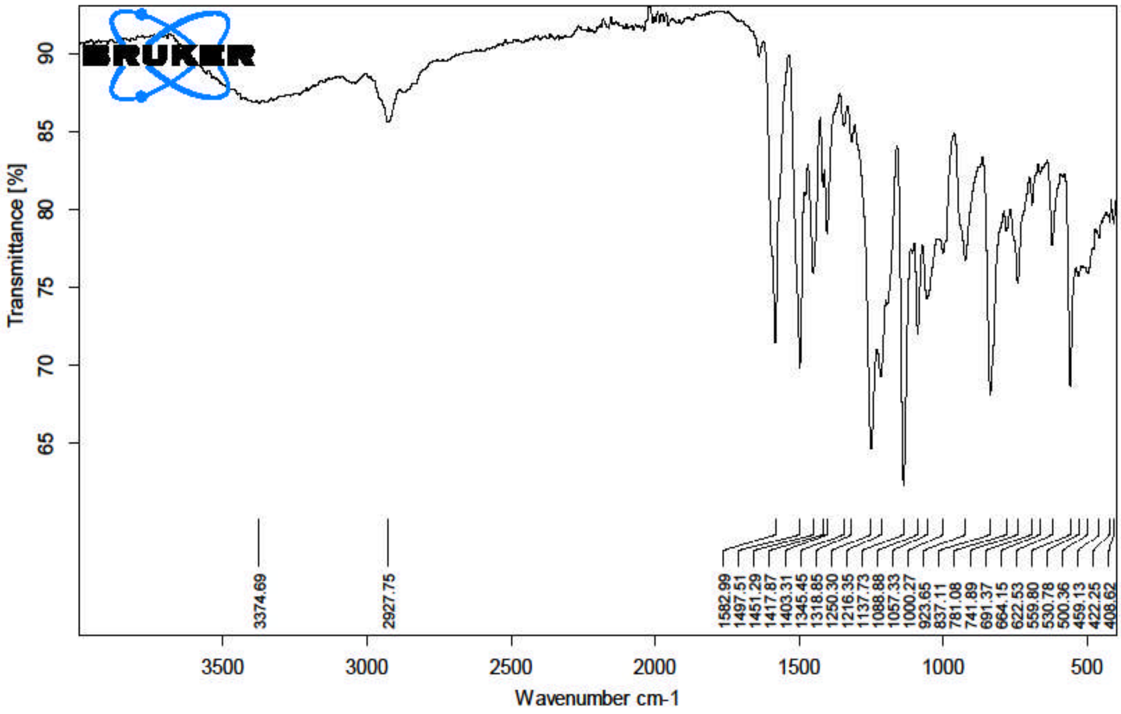

Fig. S32. Infrared spectrum of **L3**.

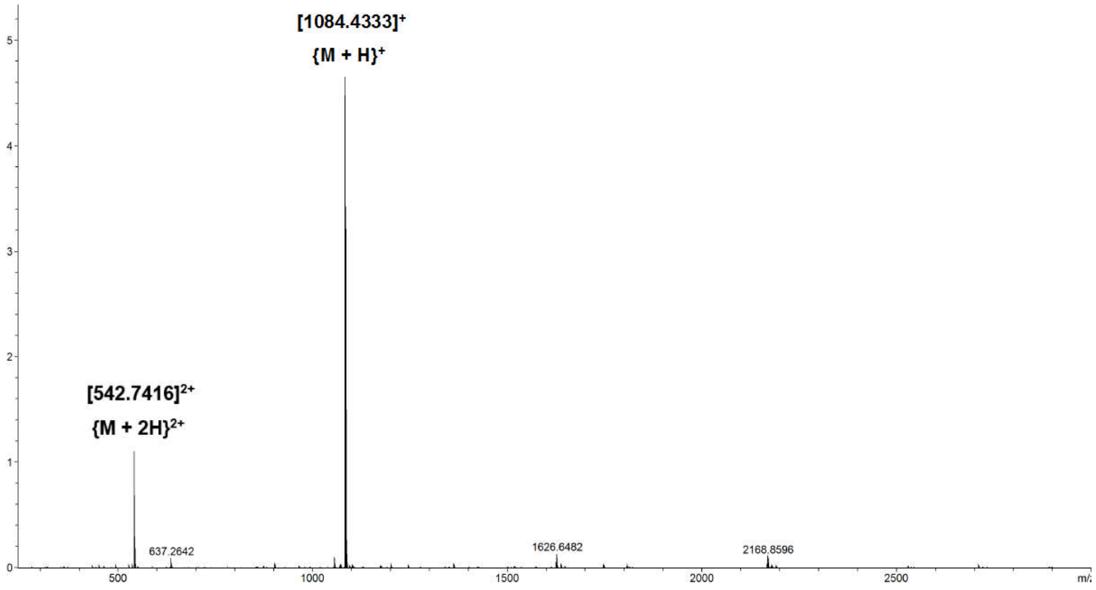

Fig. S33 Mass spectrum of **L3**.

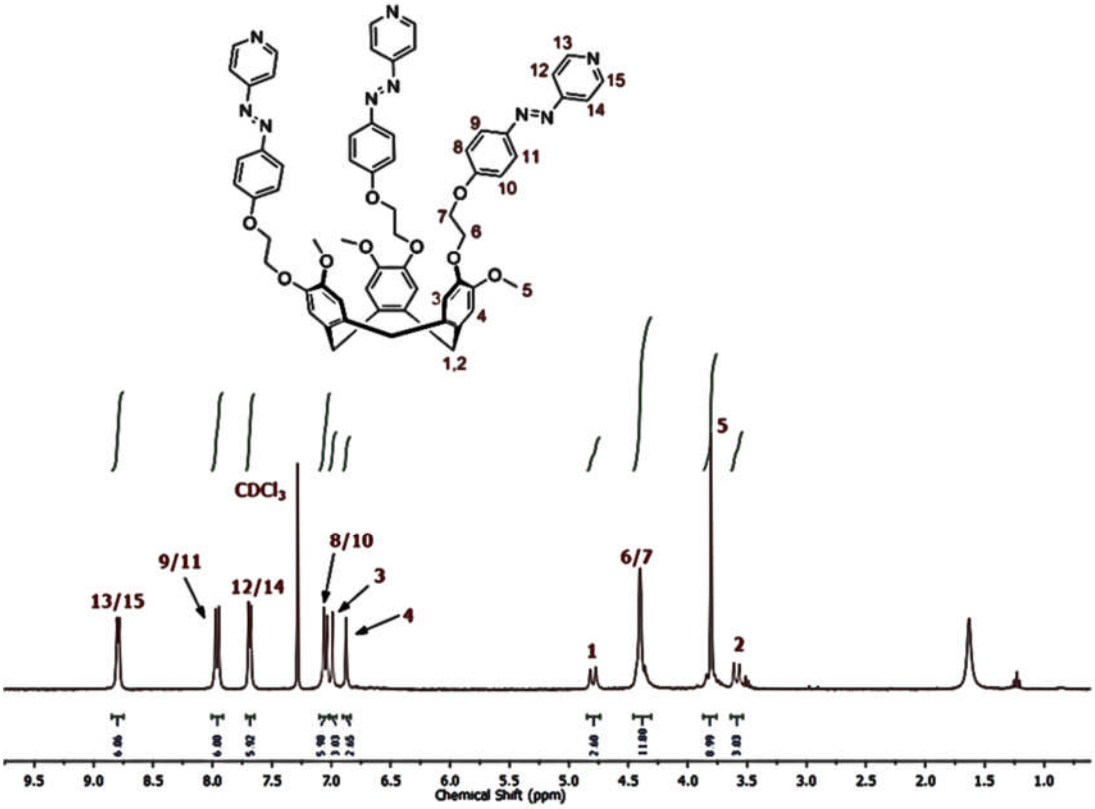

Fig. S34. ^{1}H NMR (300 MHz, $CDCl_3$) of **L3**.

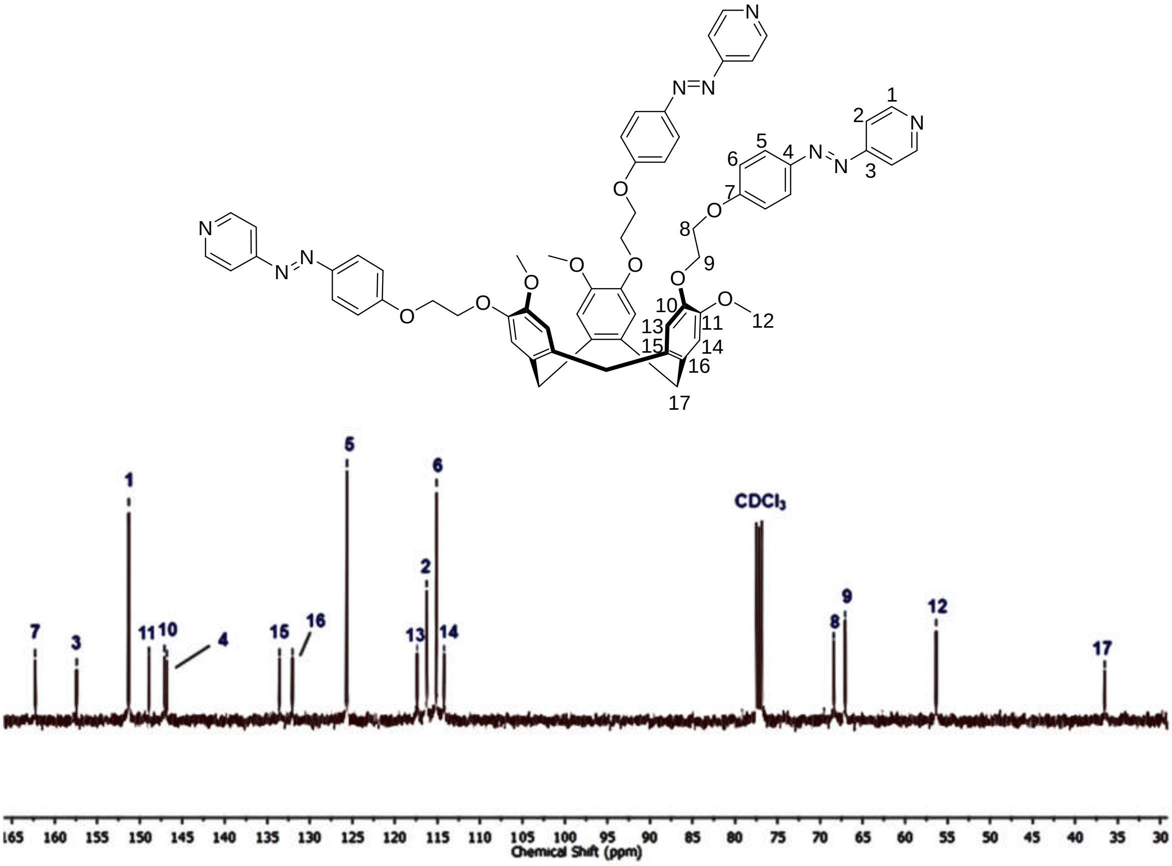

Fig. S35. ^{13}C NMR (75 MHz, $CDCl_3$) of **L3**.

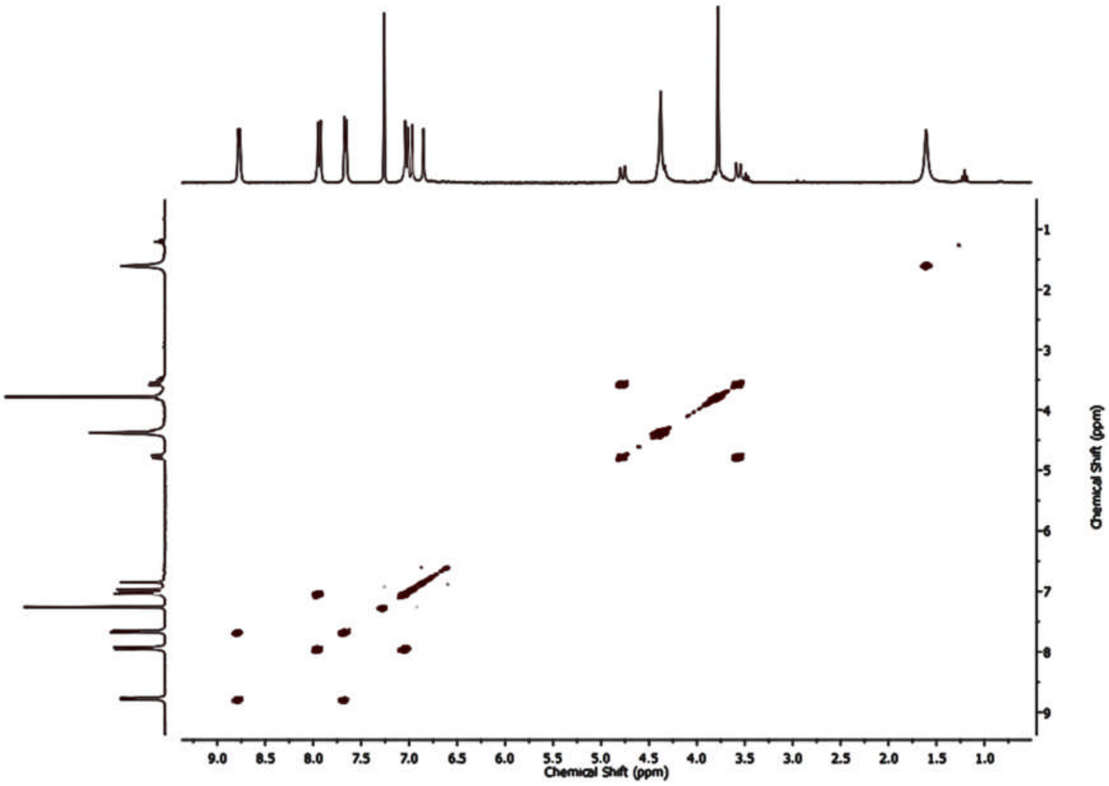

Fig. S36. ^{1}H-^{1}H COSY NMR (400 MHz, $CDCl_3$) of **L3**.

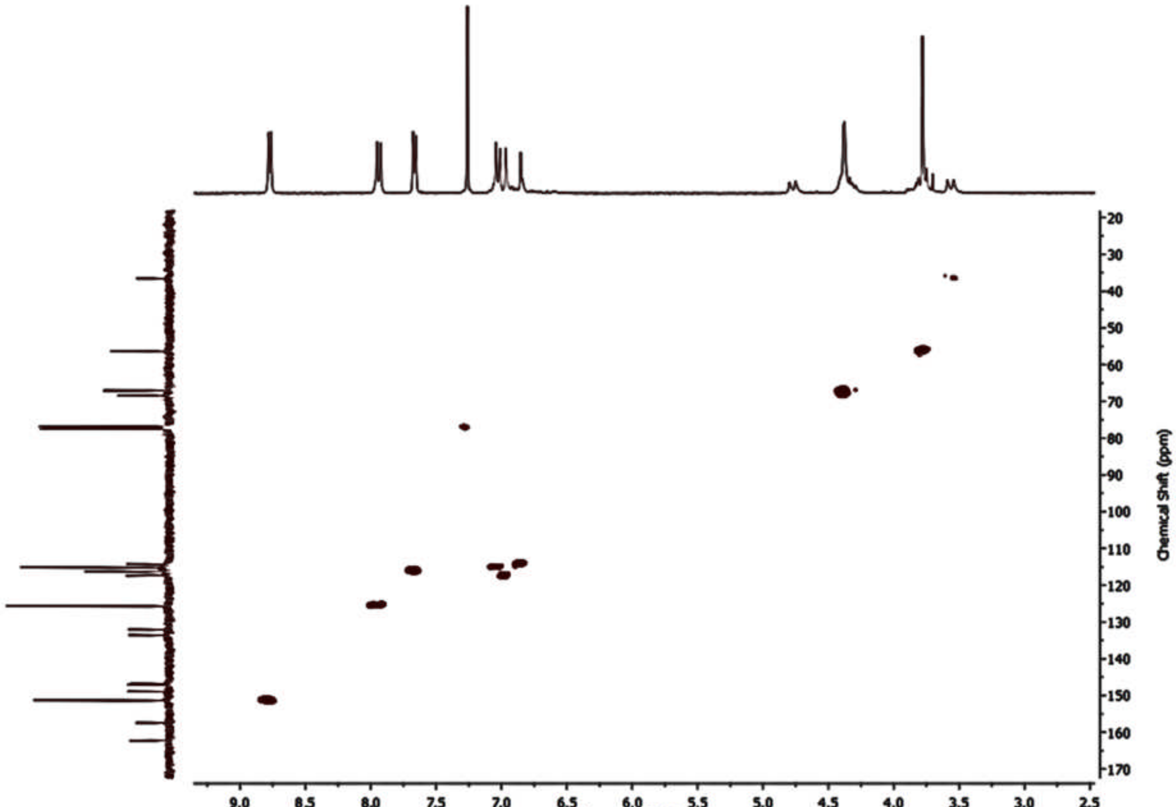

Fig. S37. ^{1}H-^{13}C HMBC NMR (400 MHz, $CDCl_3$) of **L3**.

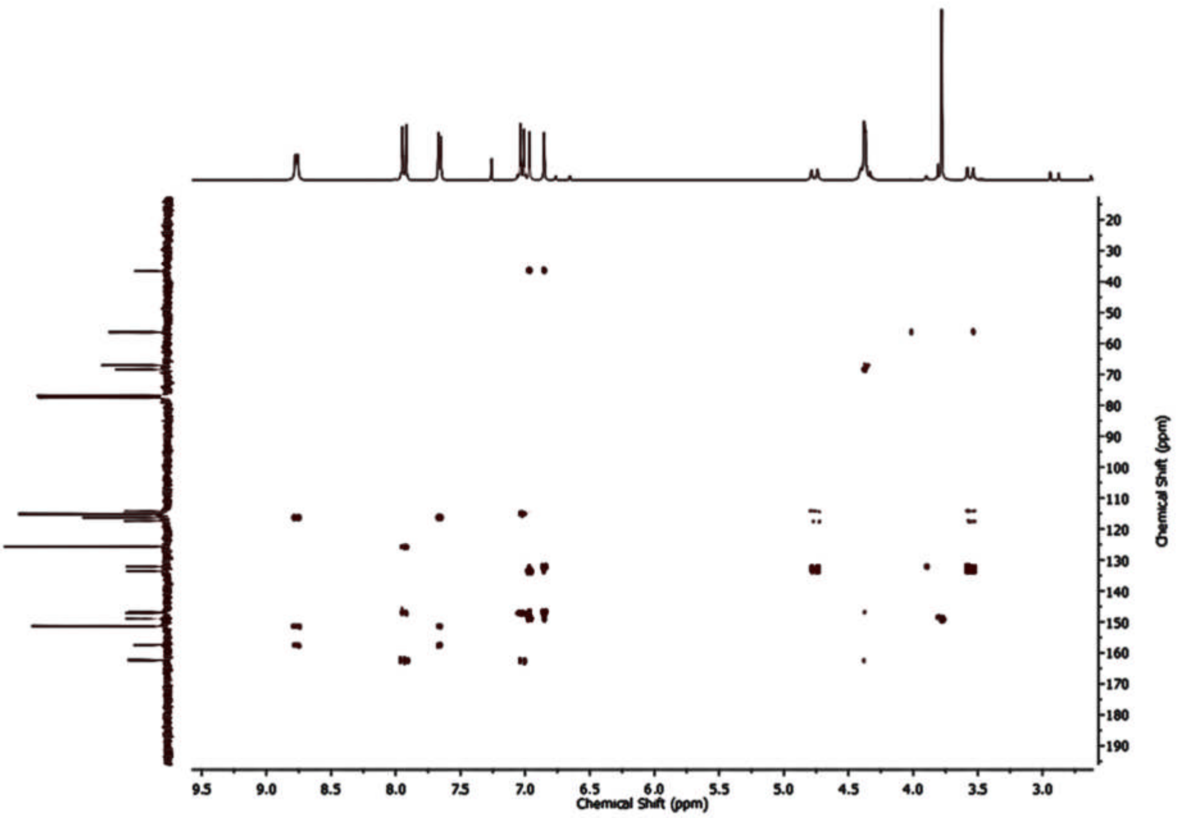

Fig. S38. ^{1}H-^{13}C HMQC NMR (400 MHz, $CDCl_3$) of **L3**.

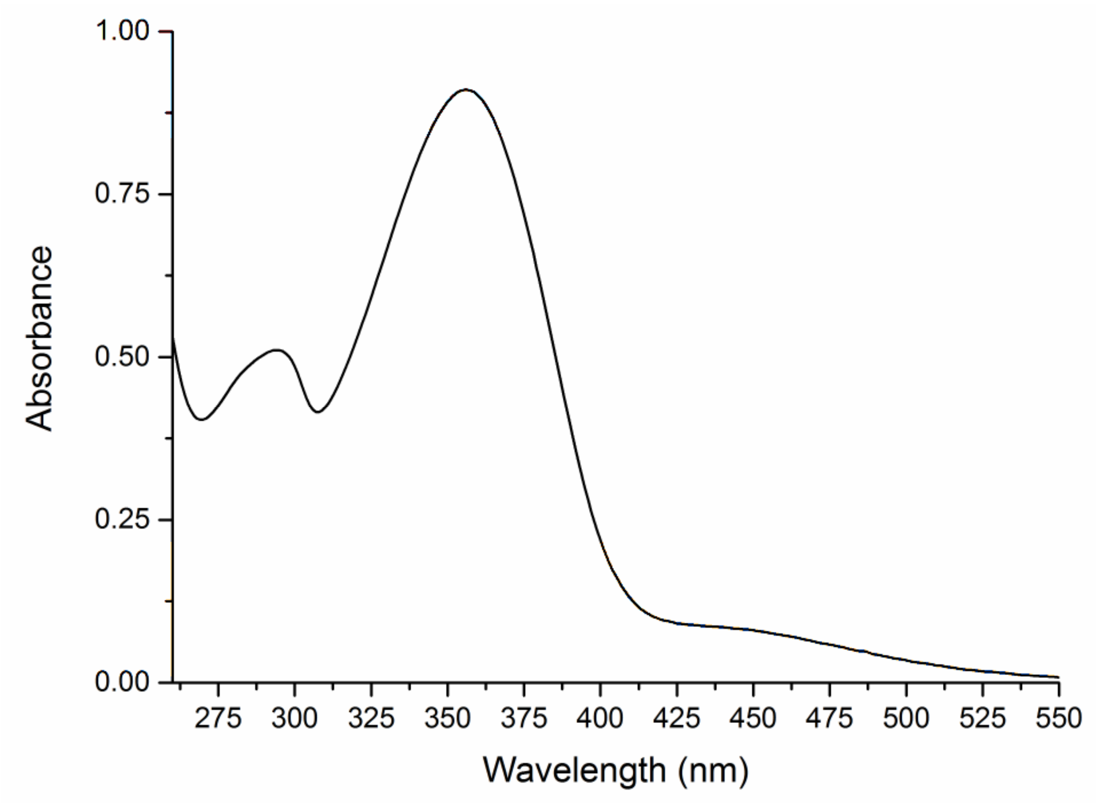

Fig. S39. UV-visible spectrum of **L3** in DMSO

2,7,12-Tris-(2-(3-pyridylazo)ethoxy)-3,8,13-trimethoxy-10,15-dihydro-2H-tribenzo[*a,d,g*]cyclononene (L4)

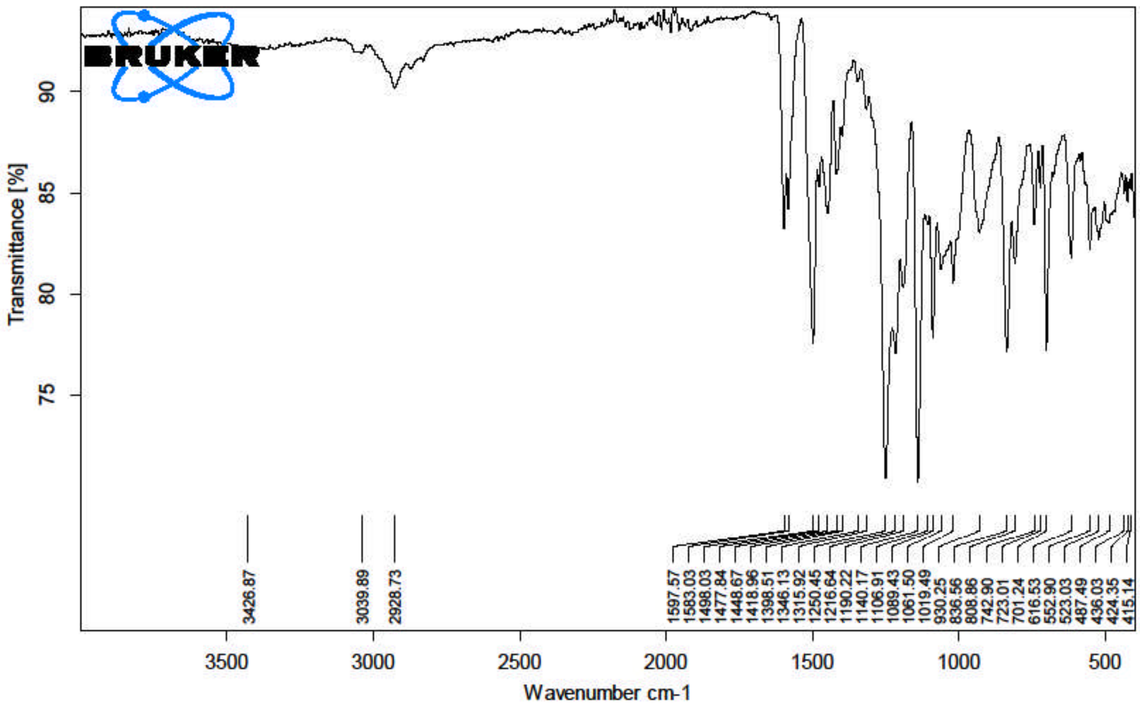

Fig. S40. Infrared spectrum of **L4**.

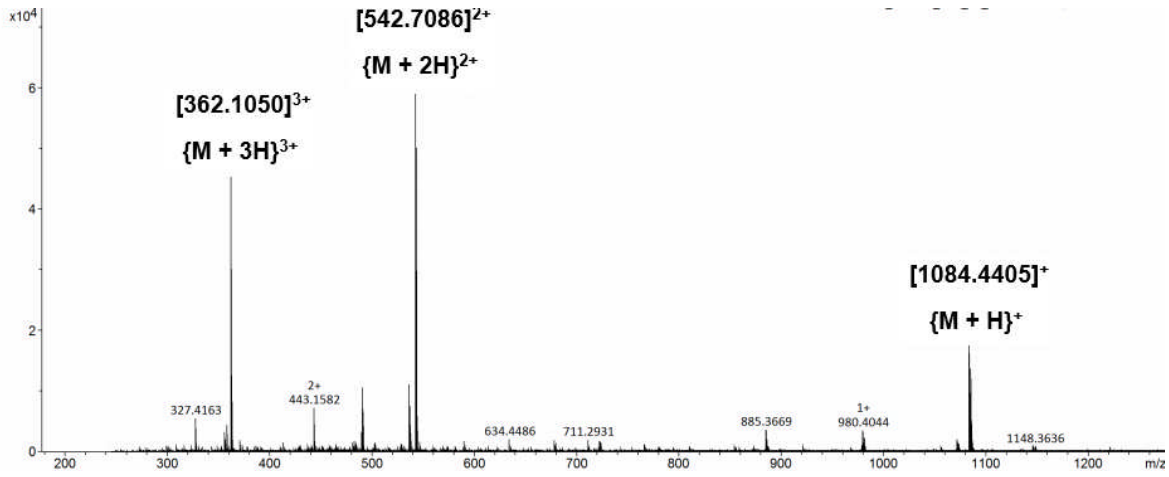

Fig. S41 Mass spectrum of **L4**.

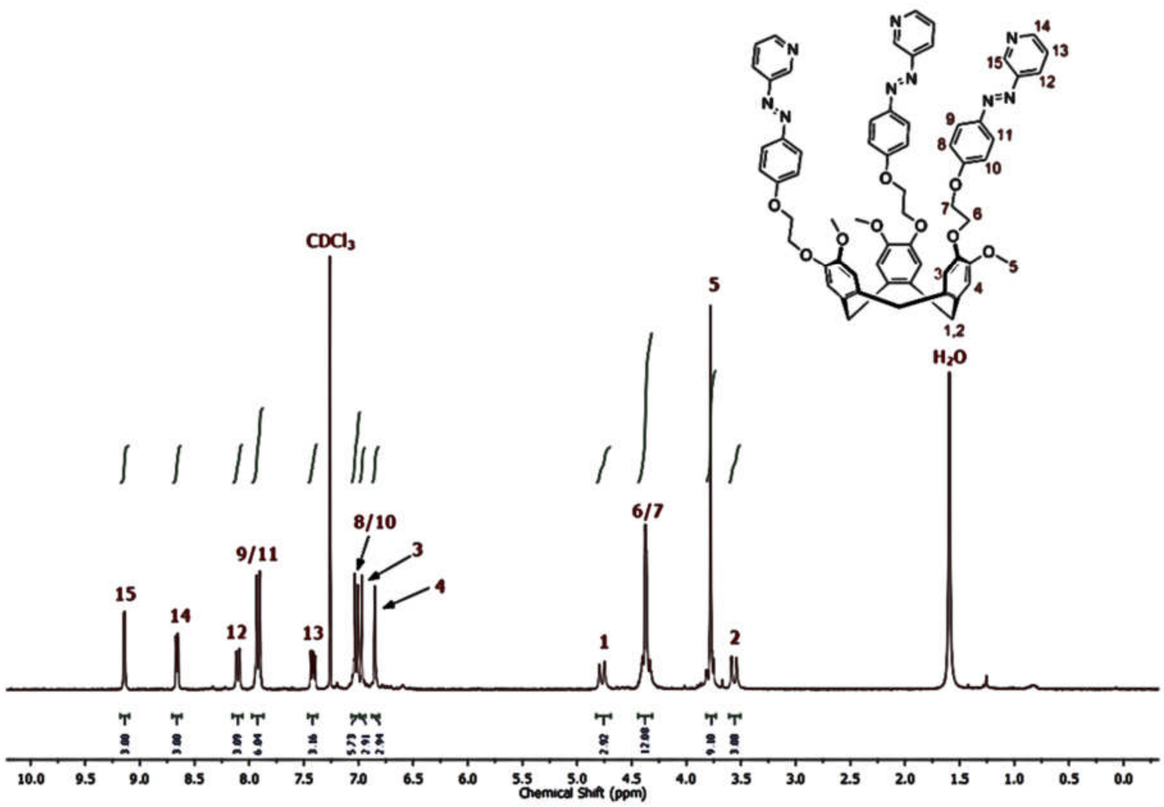

Fig. S42. ^{1}H NMR (400 MHz, $CDCl_3$) of **L4**.

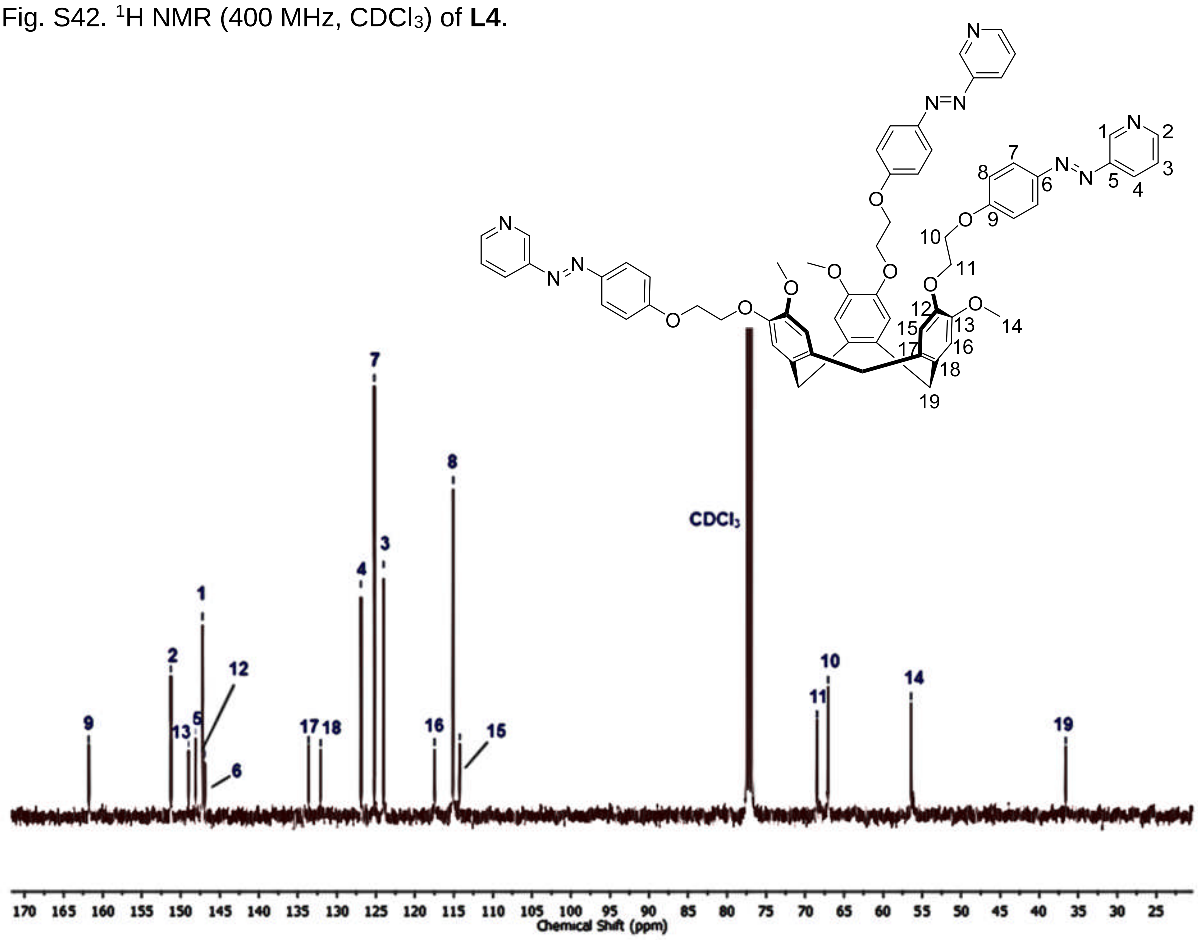

Fig. S43. ^{13}C NMR (101 MHz, $CDCl_3$) of **L4**.

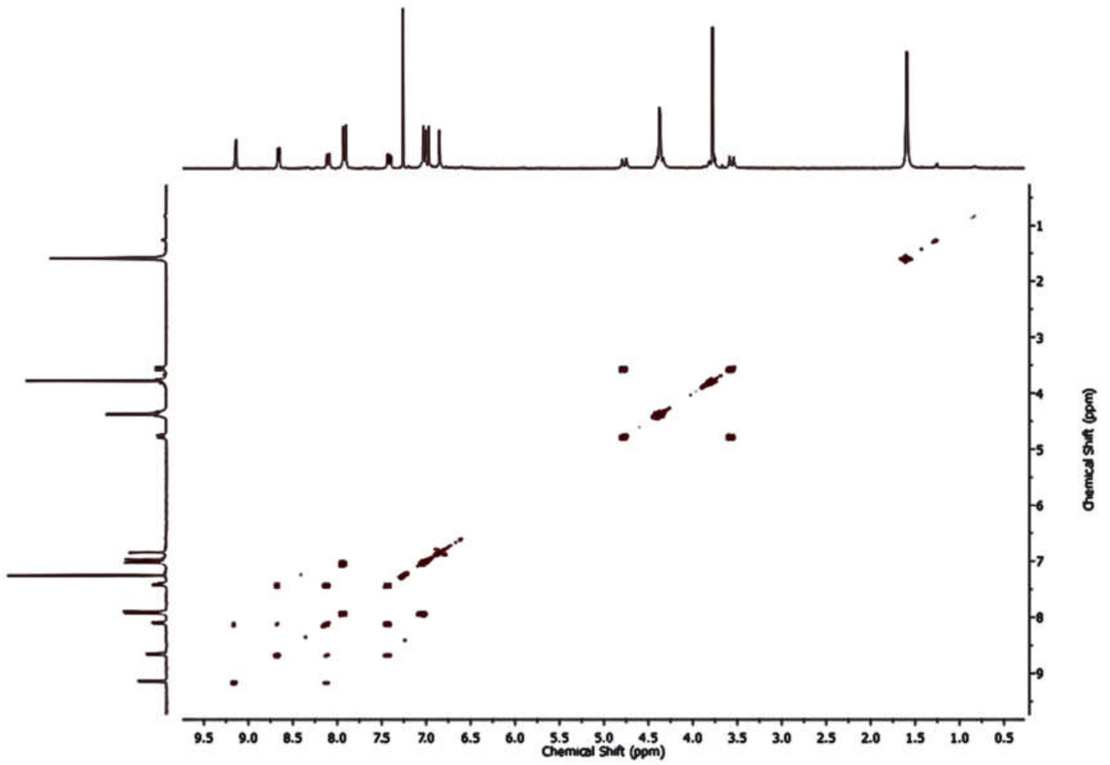

Fig. S44. ^{1}H-^{1}H COSY NMR (400 MHz, $CDCl_3$) of L4.

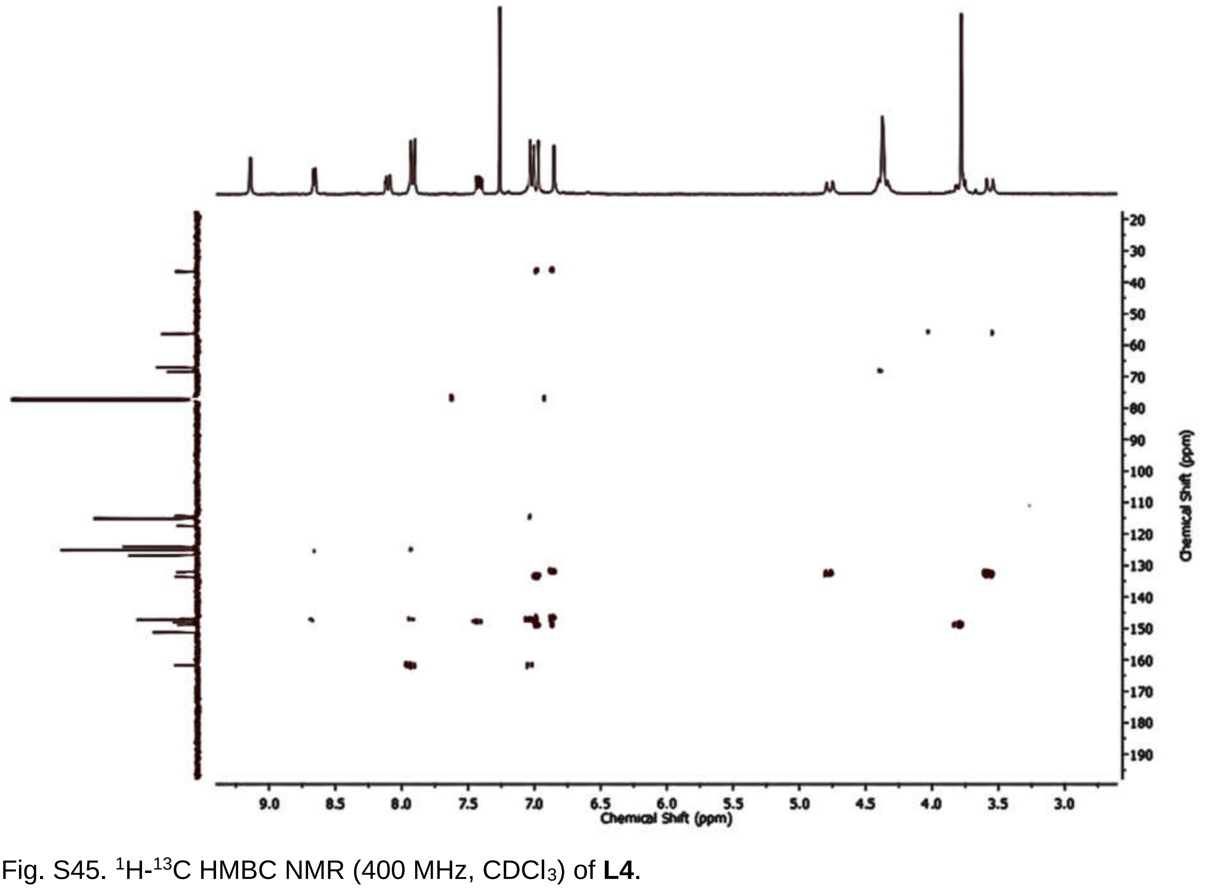

Fig. S45. ^{1}H-^{13}C HMBC NMR (400 MHz, $CDCl_3$) of **L4**.

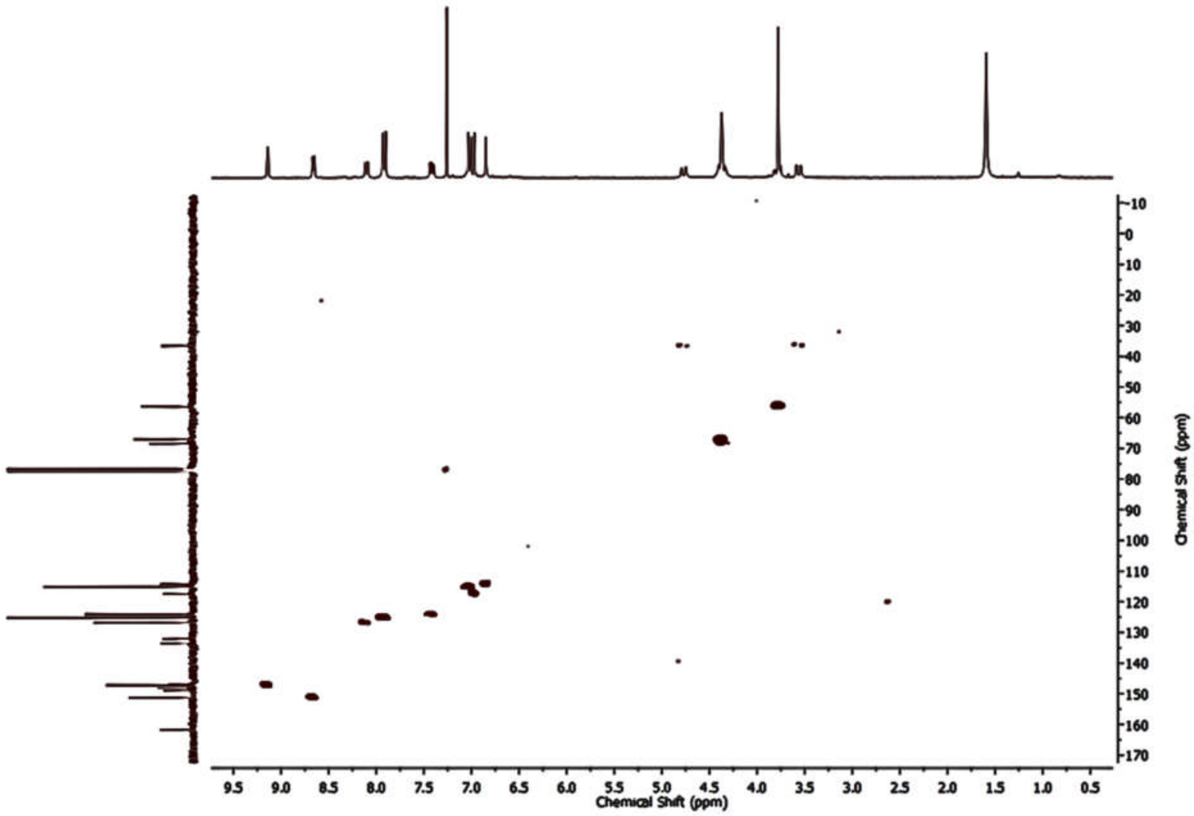

Fig. S46. ^{1}H-^{13}C HMQC NMR (400 MHz, $CDCl_3$) of **L4**.

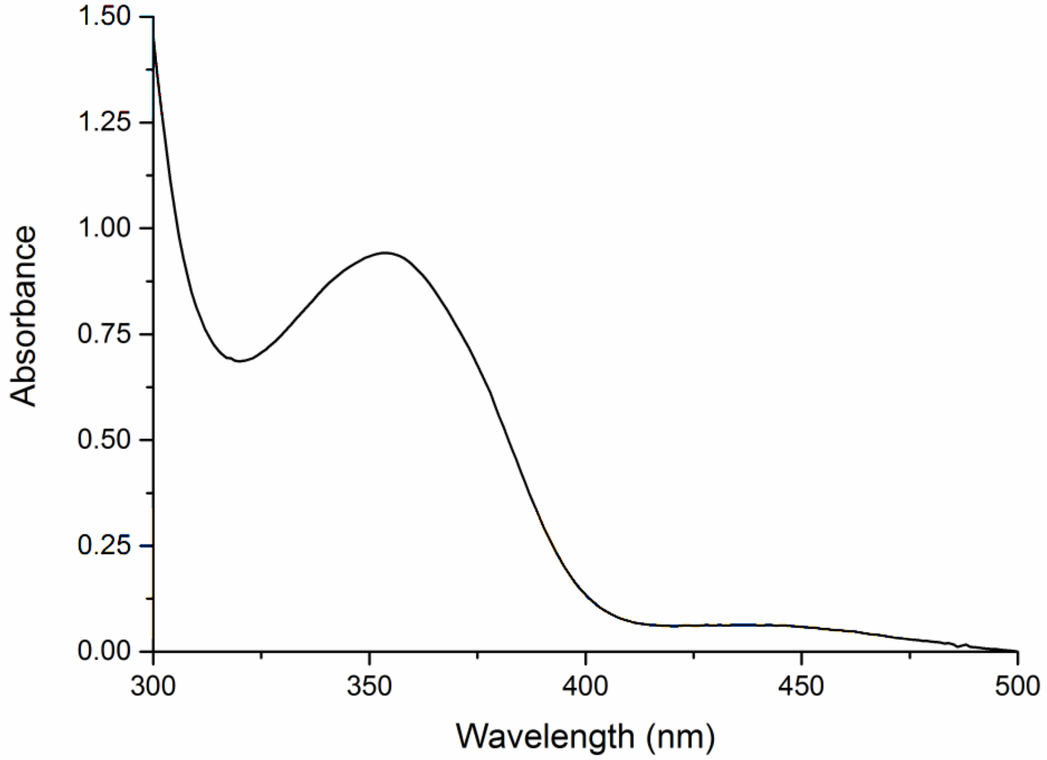

Fig. S47. UV-visible spectrum of **L4** in DMSO

[{Ir(ppy)$_2$}$_3$ (L2)$_2$]$^{3+}$·3PF$_6^-$ coordination cage (C1)

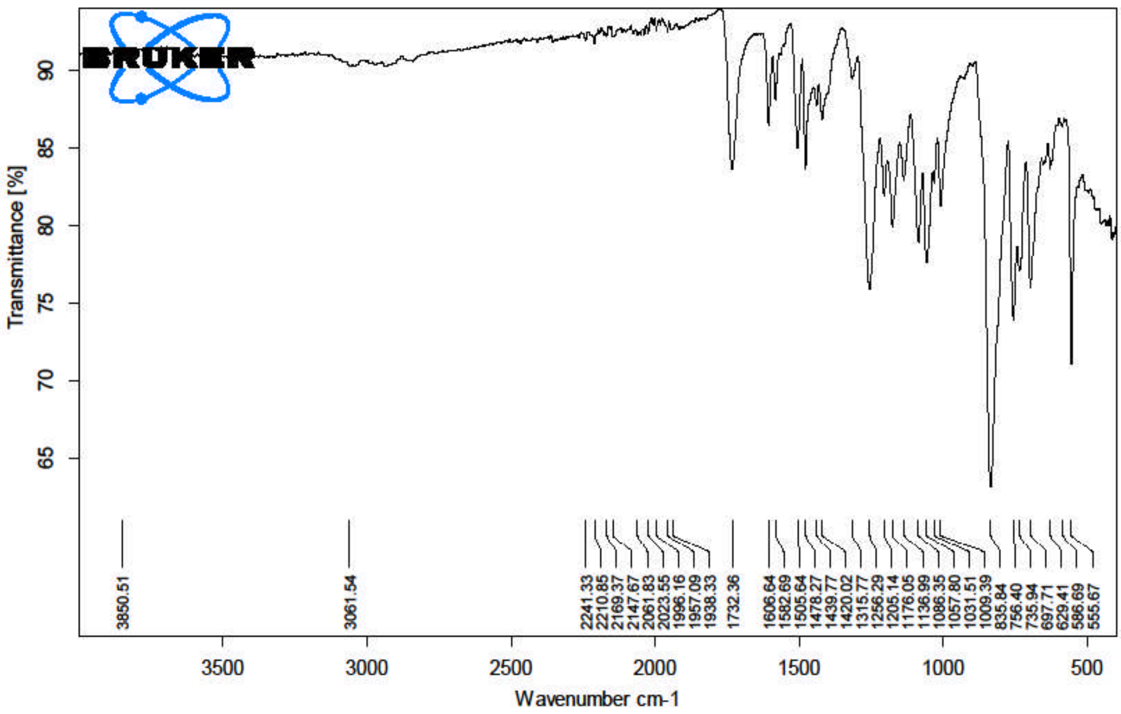

Fig. S48. Infrared spectrum of **C1**.

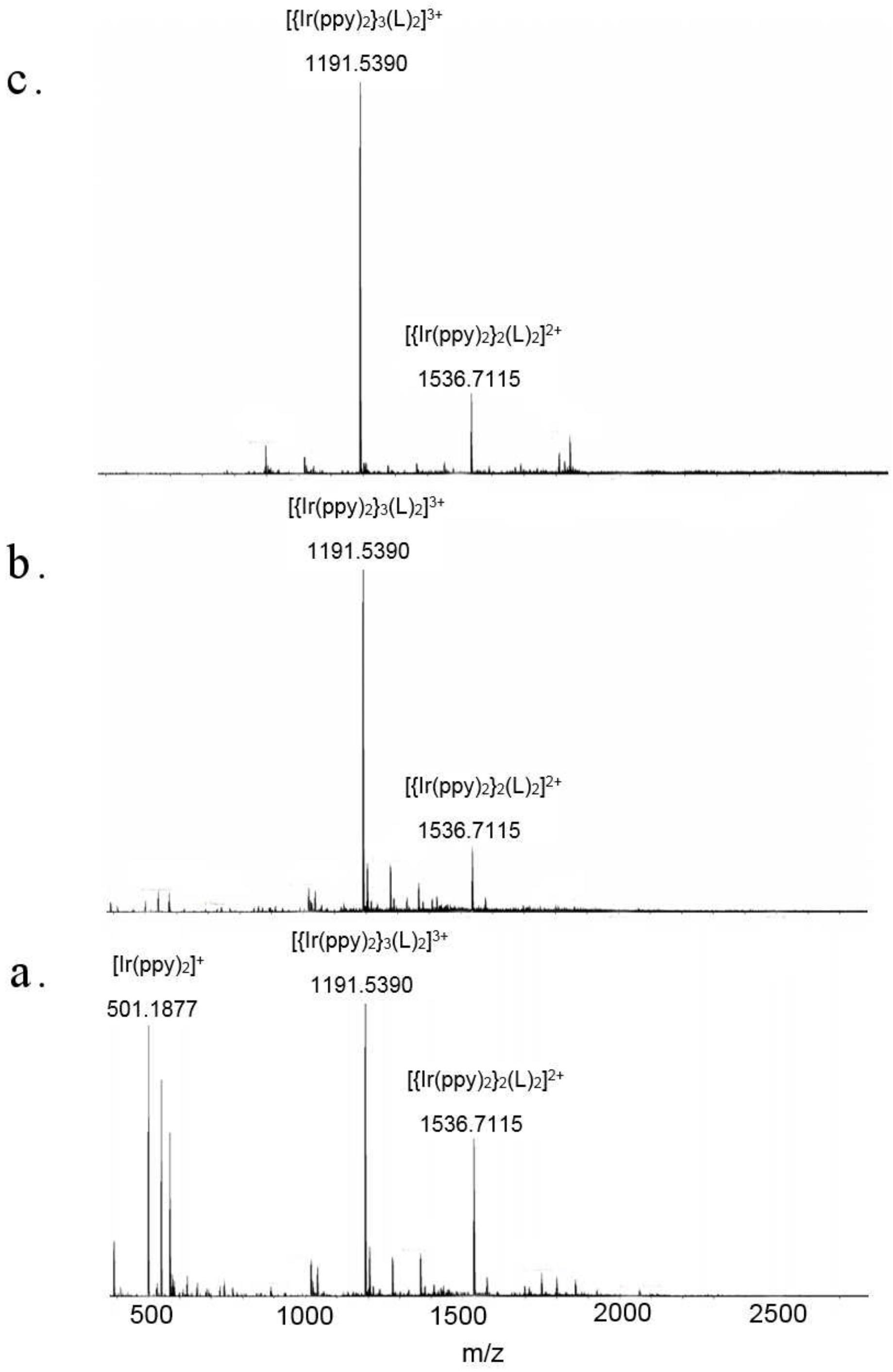

Fig. S49. Mass spectrum of **C1** in $MeNO_2$ after (a) initial mixing; (b) 1 hr equilibration; (c) 24 hrs equilibration.

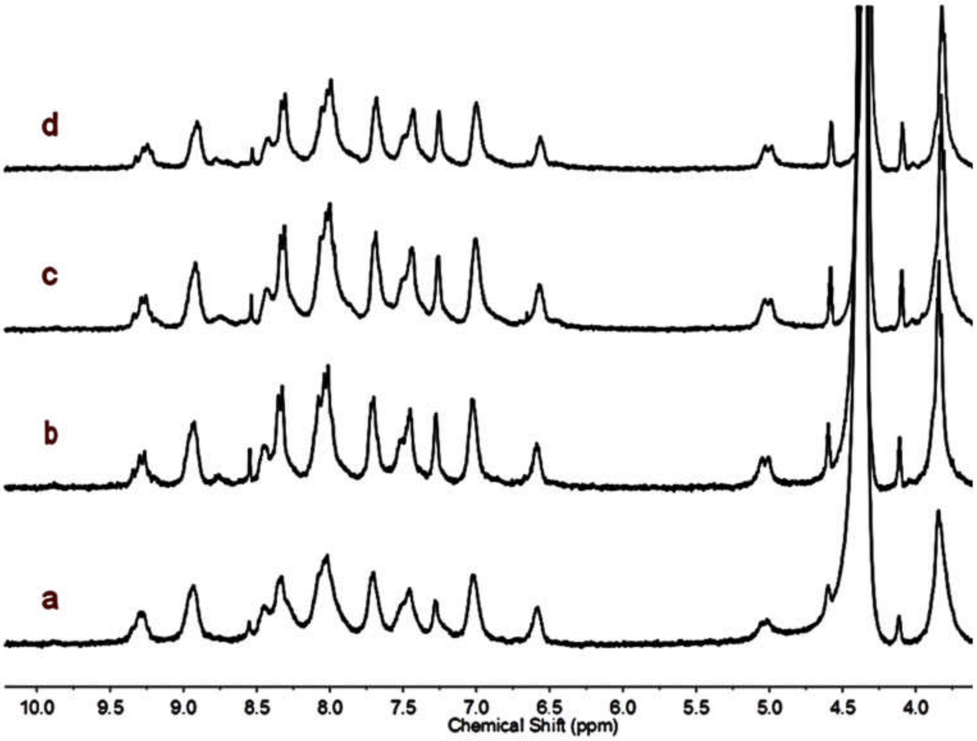

Fig. S50. Section of the ^{1}H NMR (300 MHz, $MeNO_2$-d^3) spectrum of **C1** showing cage is stable in solution for long periods. (a) after 24 hrs equilibration; (b) after 1 month in solution; (c) after 4 months in solution; (d) after 9 months in solution.

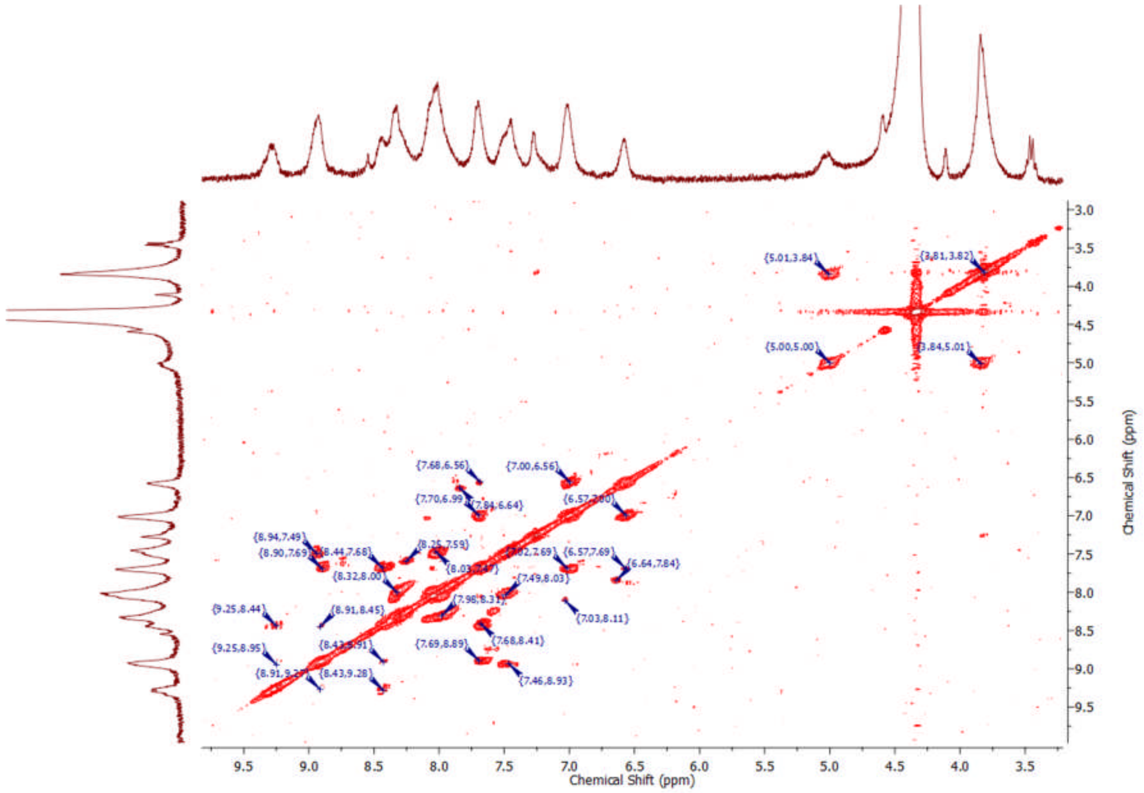

Fig. S51. ^{1}H-^{1}H COSY NMR (400 MHz, $MeNO_2$-d^3) spectrum of **C1**.

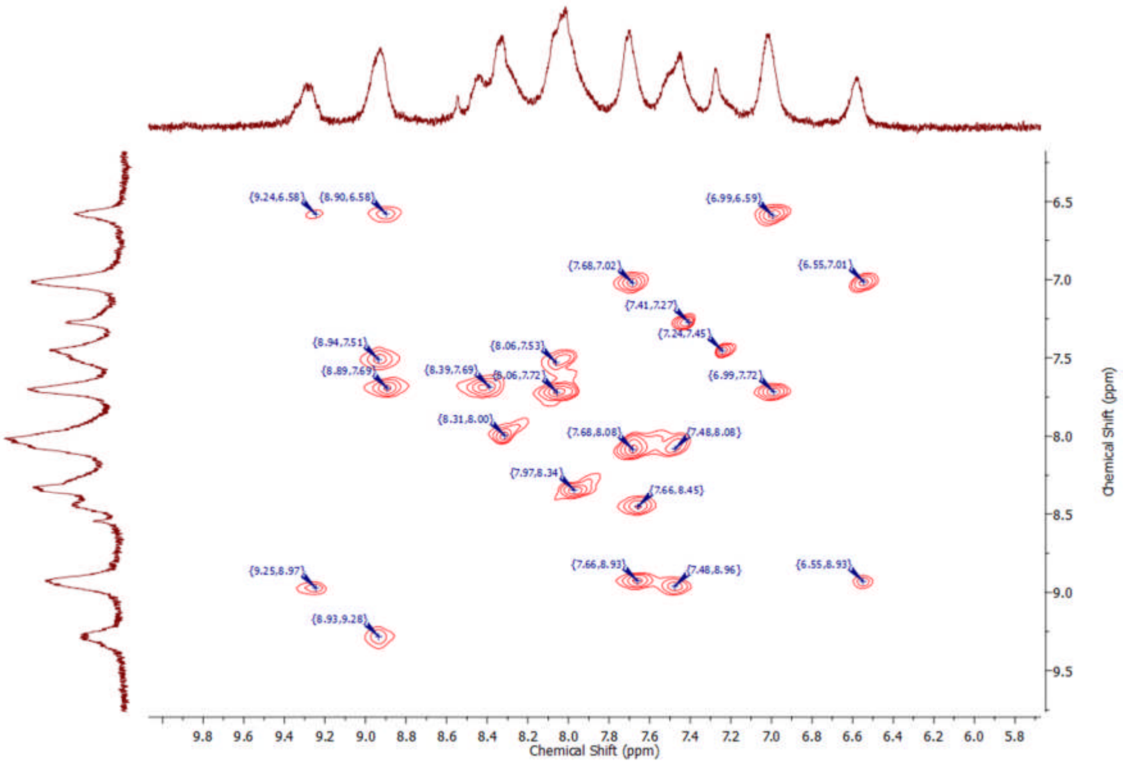

Fig. S52. ^{1}H-^{1}H ROESY NMR (500 MHz, $MeNO_2$-d^3) spectrum of **C1**.

Table S2. Assignment of through space coupling interactions observed in the ROESY NMR spectrum of cage C1 and corresponding distances found in molecular model.

ROE interaction	Assignment (NMR)	Distance (Å)[a]	Origin[b]
6.546---7.018	H_8---H_6 + H_7	2.487/4.311	Ppy---Ppy
6.548---8.931[c]	H_8---H_b + H_1	2.652/3.503	L2---Ppy
6.989---7.719	H_6 + H_7---H_c[d] + H_5	2.472/4.304	Ppy---Ppy
7.242---7.465	H_h---H_i	1.706	L2---L2
7.678---8.084	H_c[d] + H_5---H_e[d] + H_3	4.657	Ppy---Ppy
7.657---8.453	H_c + H_5[d]---H_d + H_f[d]	2.450	L2---L2
7.657---8.926	H_c + H_5[d]--- H_b + H_1[d]	2.513	L2---L2
8.034---7.509	H_e[d] + H_3--- H_h[d]+ H_2	2.541	Ppy---Ppy
8.929---7.509	H_b + H_1--- H_h[d]+ H_2	4.504/2.586	Ppy---Ppy
8.939---9.279[c]	H_b + H_1---H_a	4.174/2.411	L2---Ppy
9.233---6.584[c]	H_a---H_8	5.143	L2---Ppy

[a] Distances calculated from model; [b] Likely origin selected from closest interaction where overlapping peaks occur. [c] Key coupling - interaction likely to originate from couplings between protons on phenylpyridine unit and ligand L2; [d] Proton likely too distant to make a significant contribution to the observed ROE.

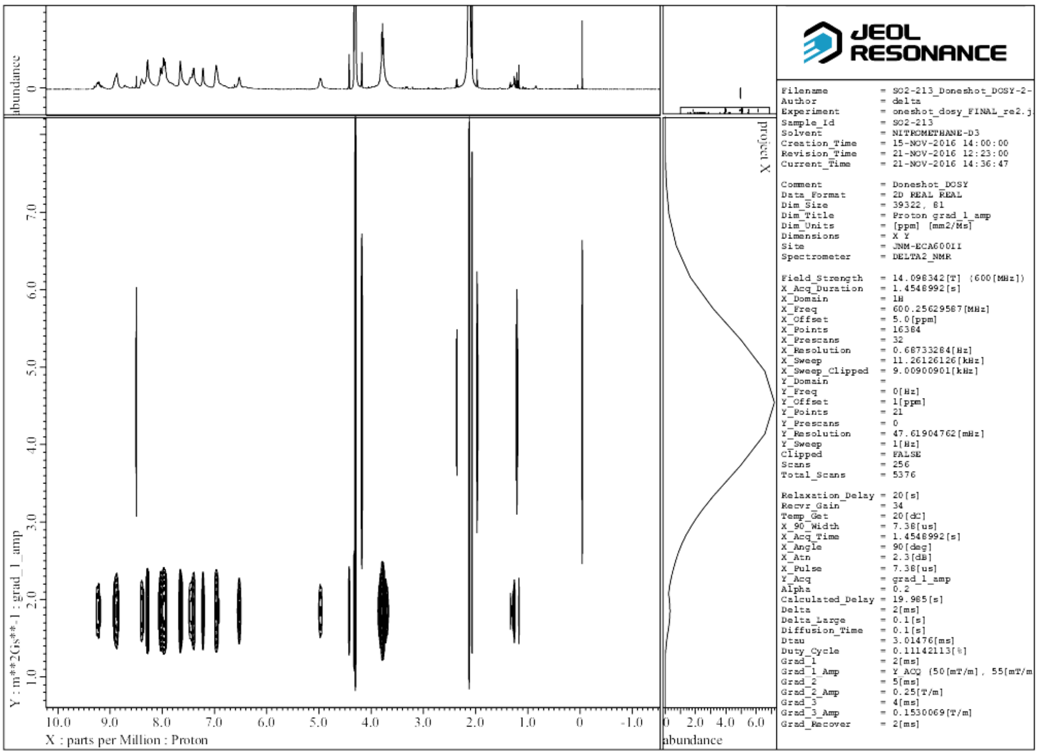

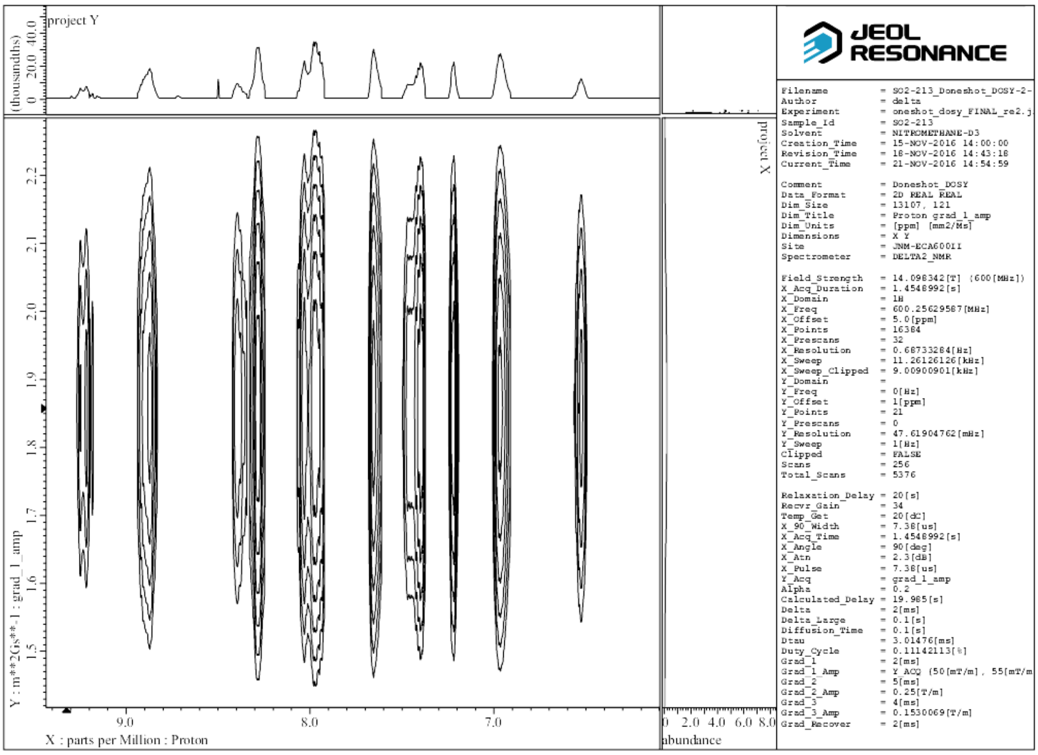

Fig. S53. ^{1}H DOSY NMR (600 MHz, $MeNO_2$-d[3]) spectrum of **C1**.

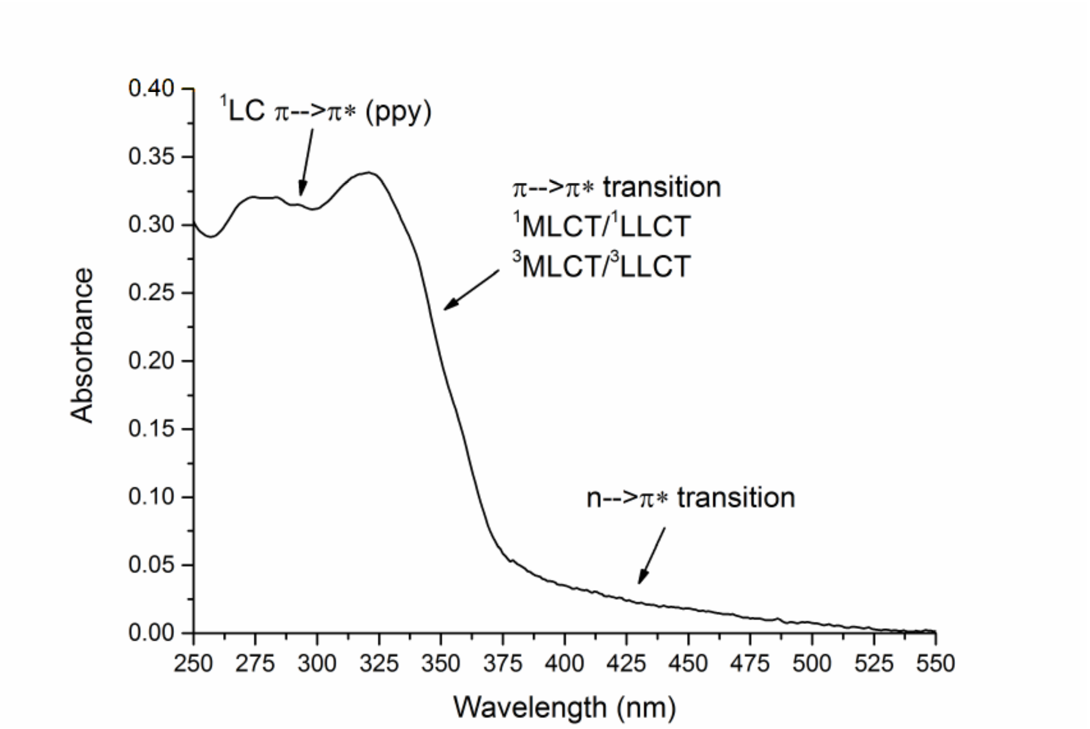

Fig. S54. UV-visible spectrum of cage **C1** in CH_2Cl_2.

[{Ir(Meppy)$_2$}$_3$ (L2)$_2$]$^{3+}\cdot$3PF$_6^-$ coordination cage (C1-Me)

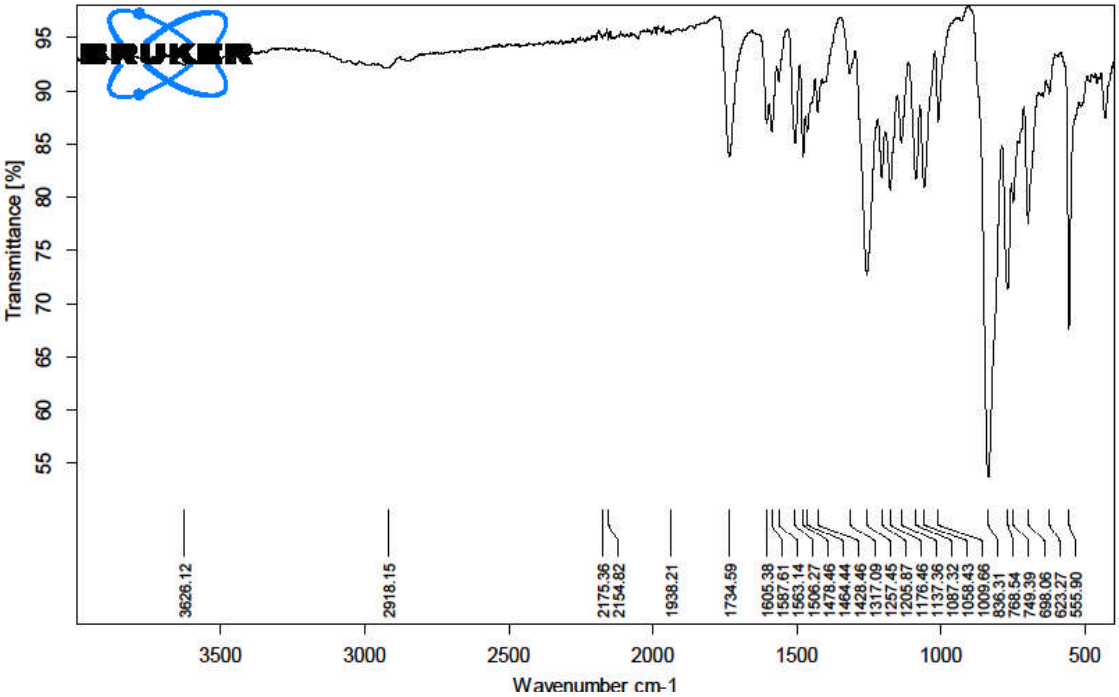

Fig. S55. Infrared spectrum of **C1-Me**.

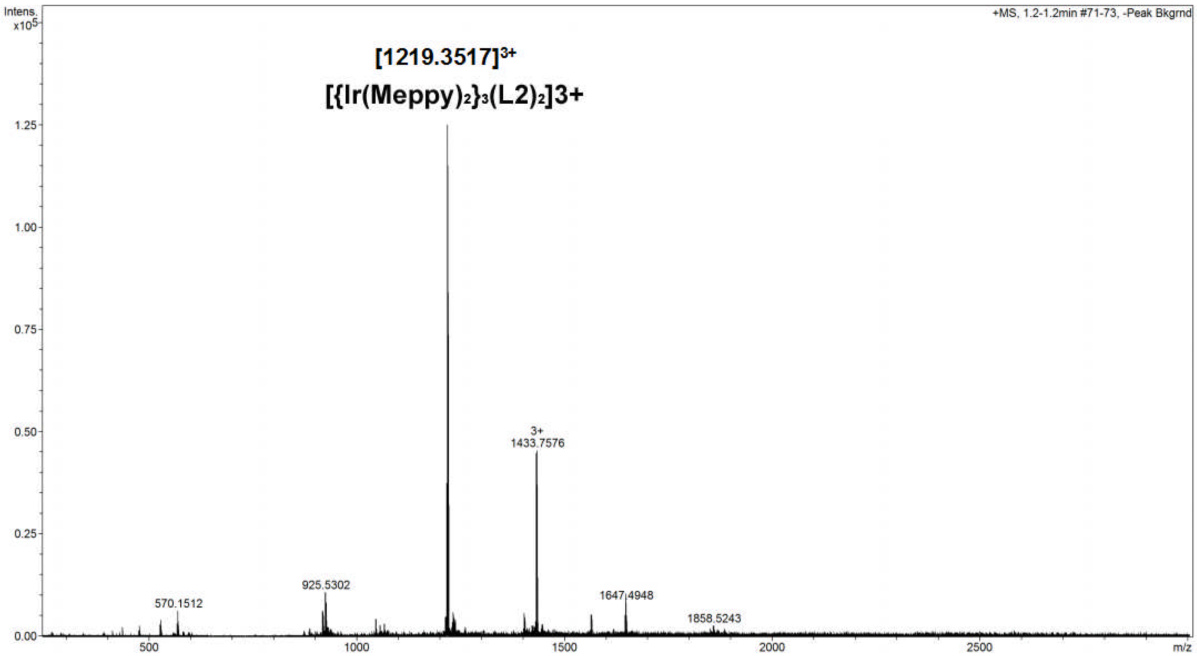

Fig. S56. Mass spectrum of **C1-Me** in $MeNO_2$ after 24hrs equilibration.

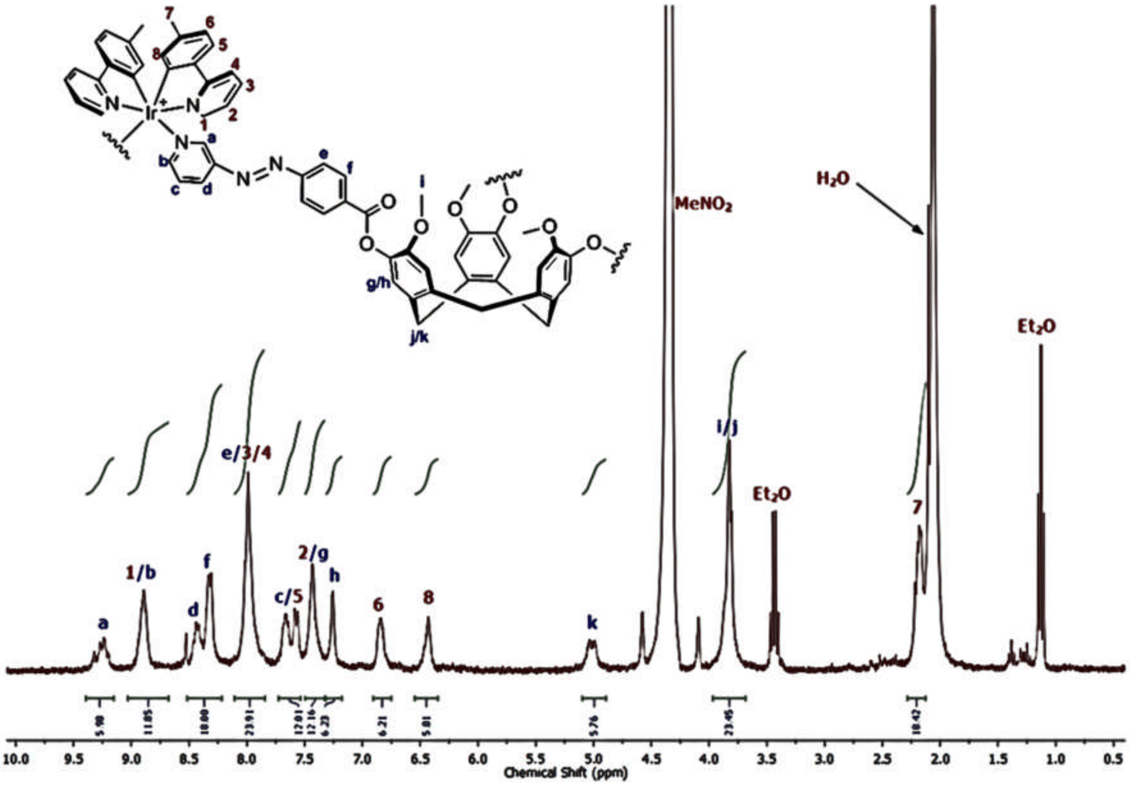

Fig. S57. ^{1}H NMR (300 MHz, $MeNO_2$-d^3) spectrum of **C1-Me**.

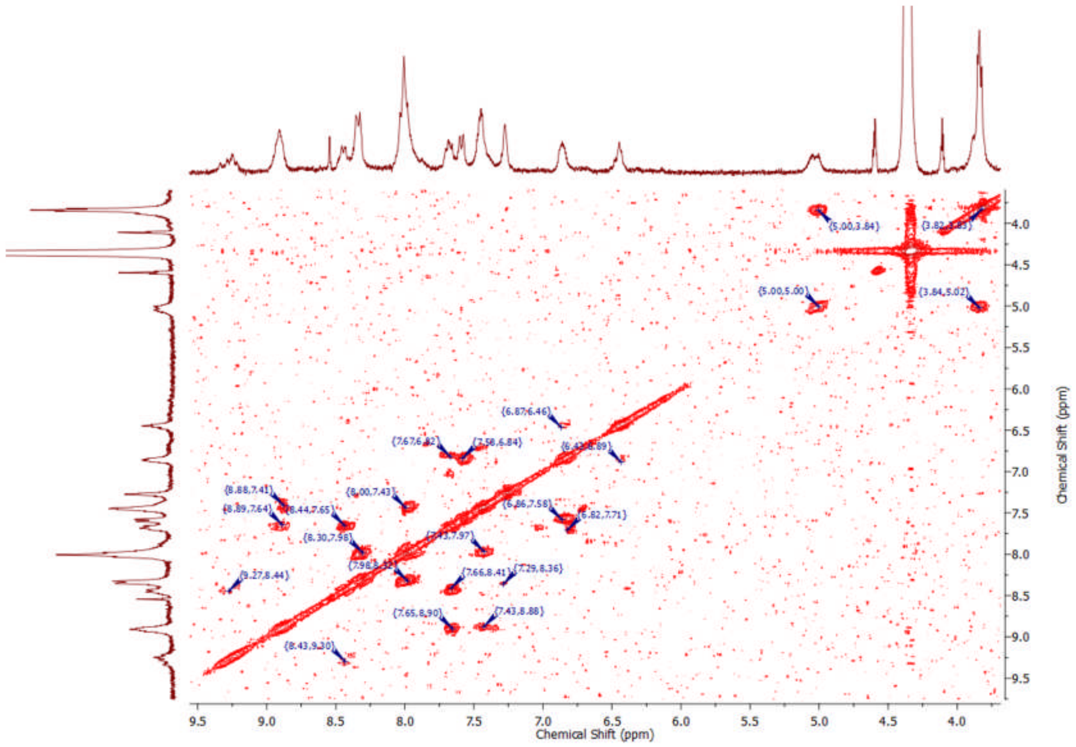

Fig. S58. ^{1}H-^{1}H COSY NMR (400 MHz, $MeNO_2$-d^3) of **C1-Me**.

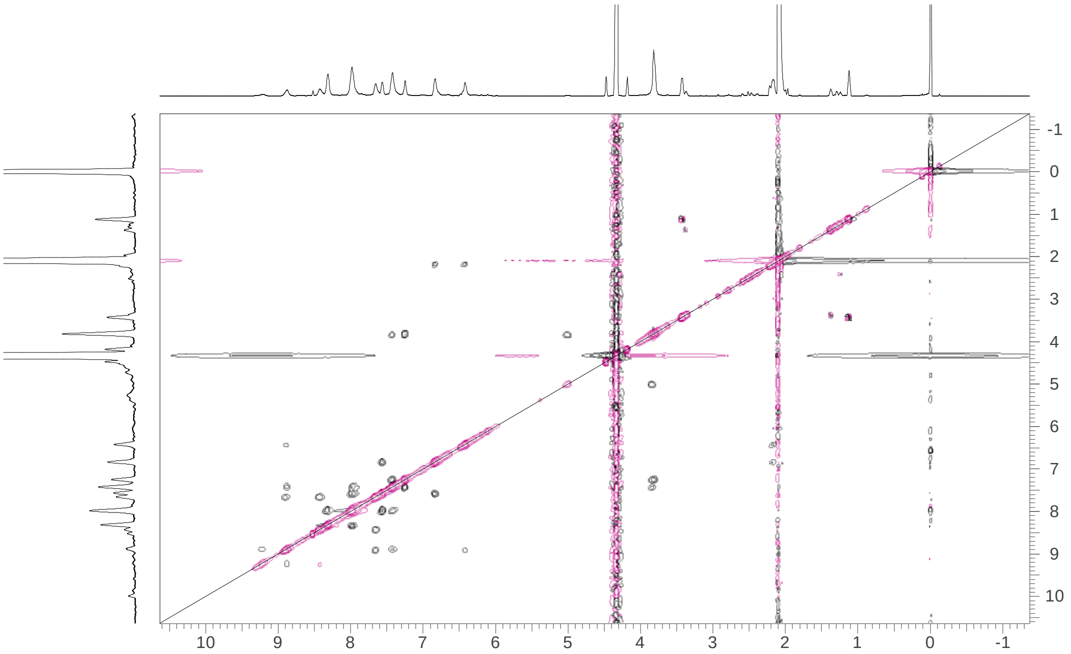

Fig. S59. ^{1}H ROESY NMR (500 MHz, $MeNO_2$-d^3) spectrum of **C1-Me**.

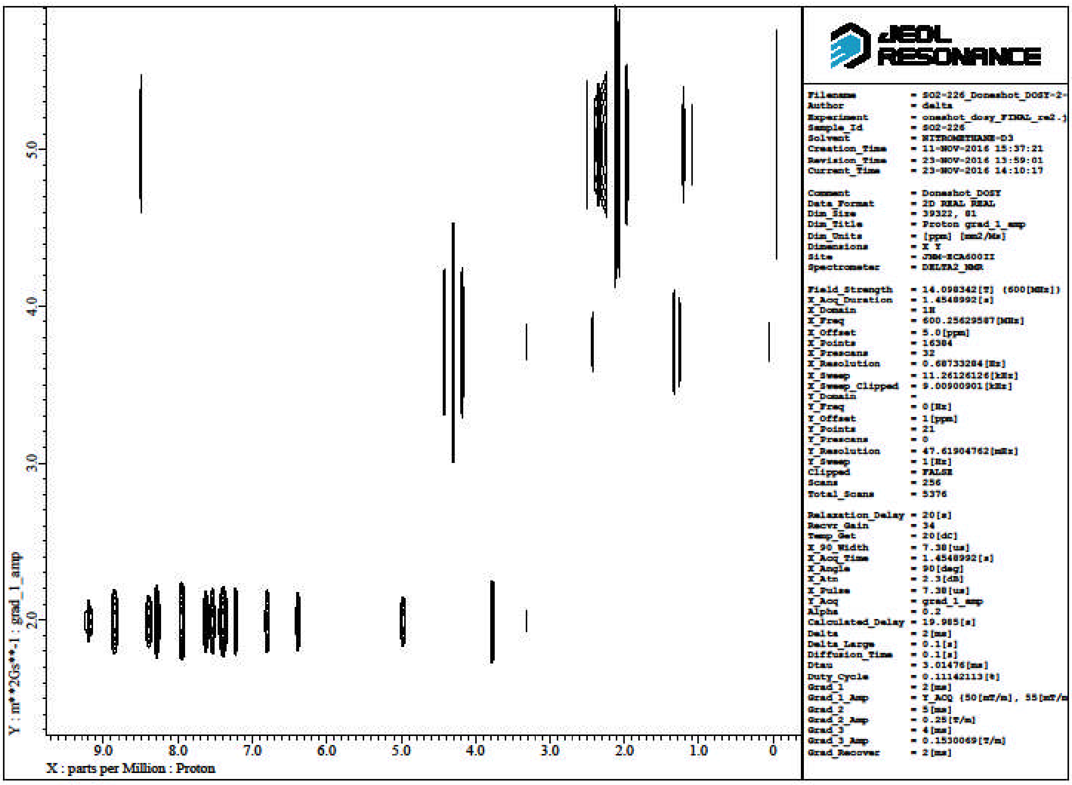

Fig. S60. ^{1}H DOSY NMR (600 MHz, $MeNO_2$-d^3) spectrum of **C1-Me**.

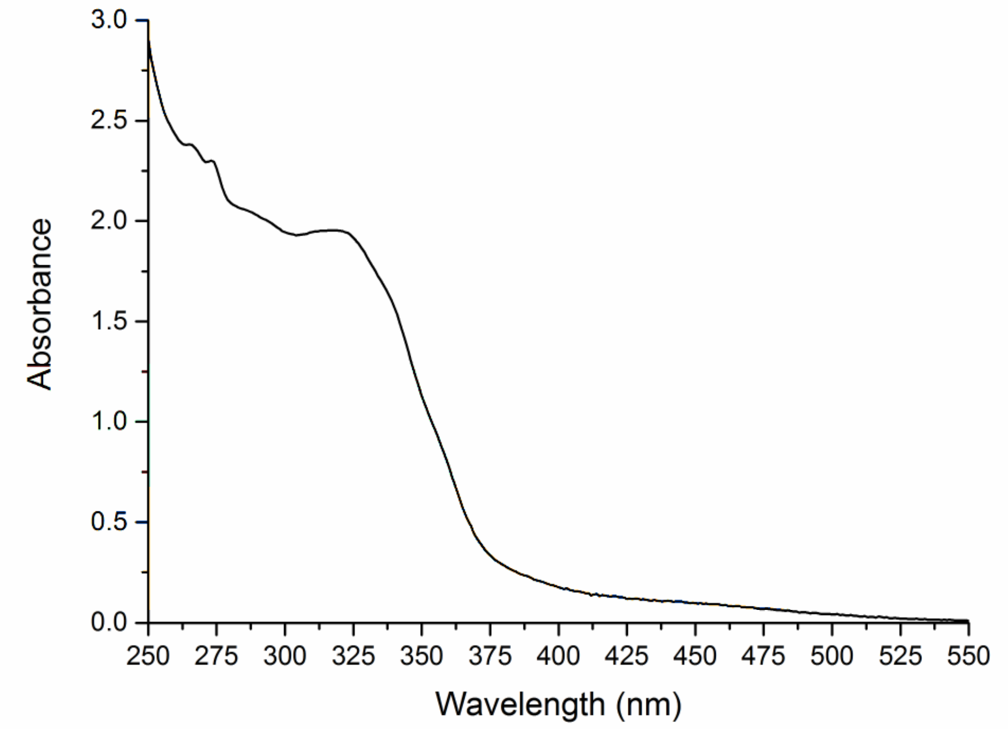

Fig. S61. UV-visible spectrum of cage **C1-Me** in CH_2Cl_2.

[{Ir(4,5,6-tFppy)$_2$}$_3$ (L2)$_2$]$^{3+}$·3PF$_6^-$ coordination cage (C1-F)

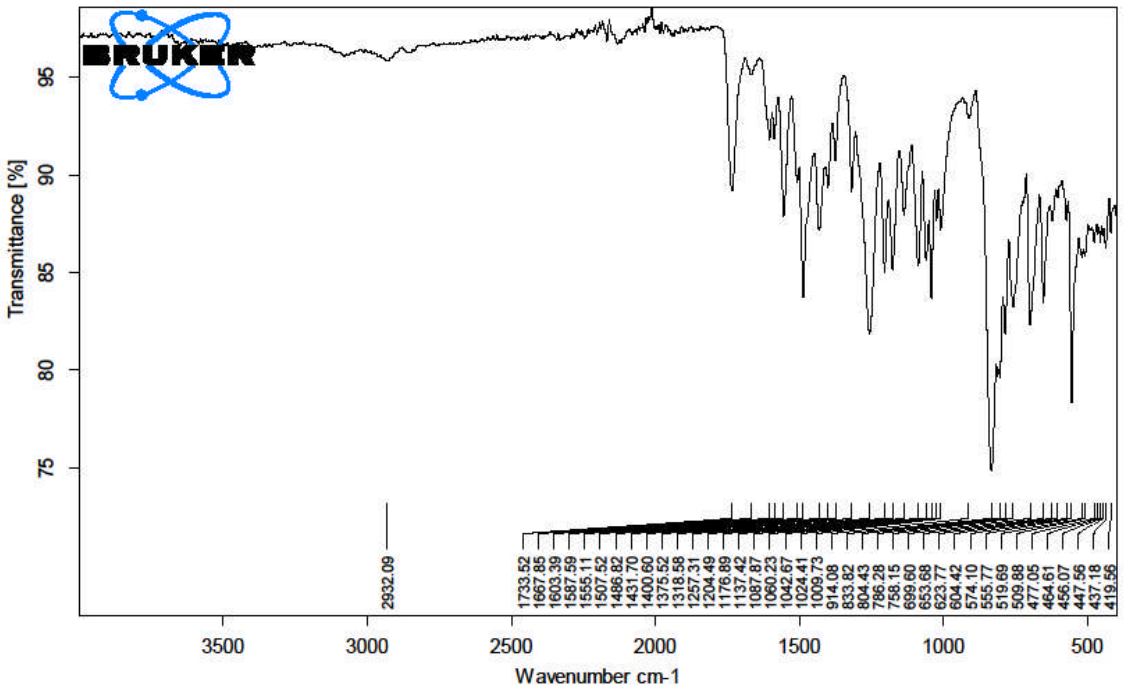

Fig. S62. Infrared spectrum of **C1-F**.

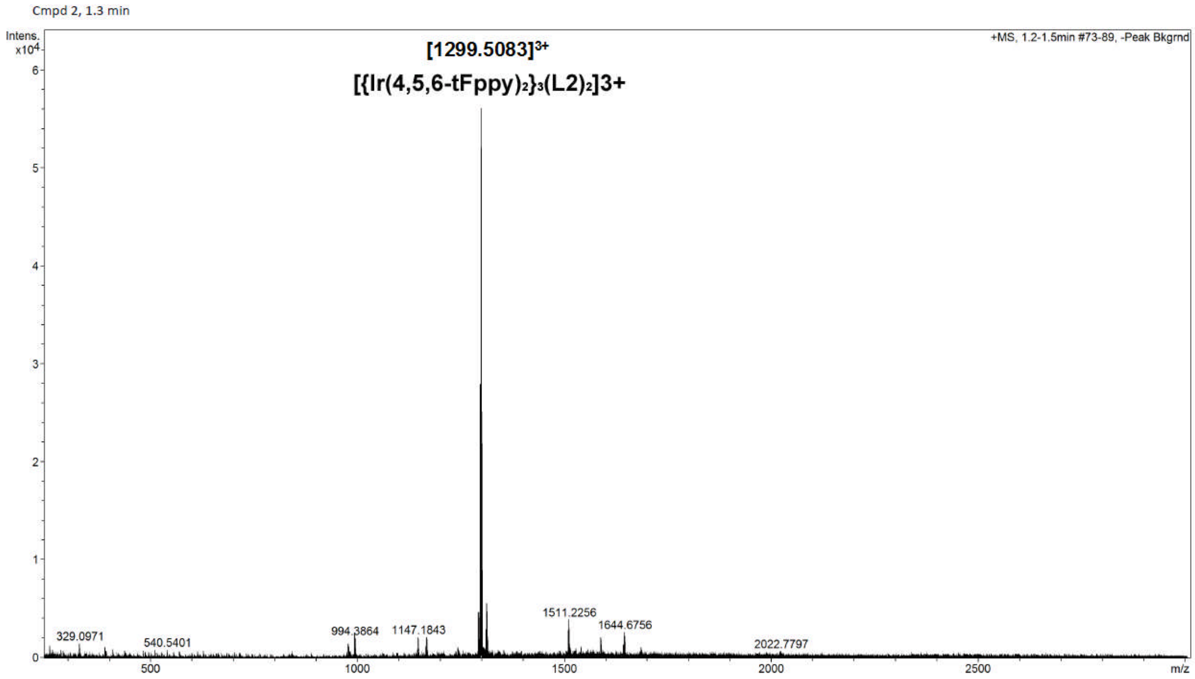

Fig. S63. Mass spectrum of **C1-F** in MeNO$_2$ after 24hrs equilibration.

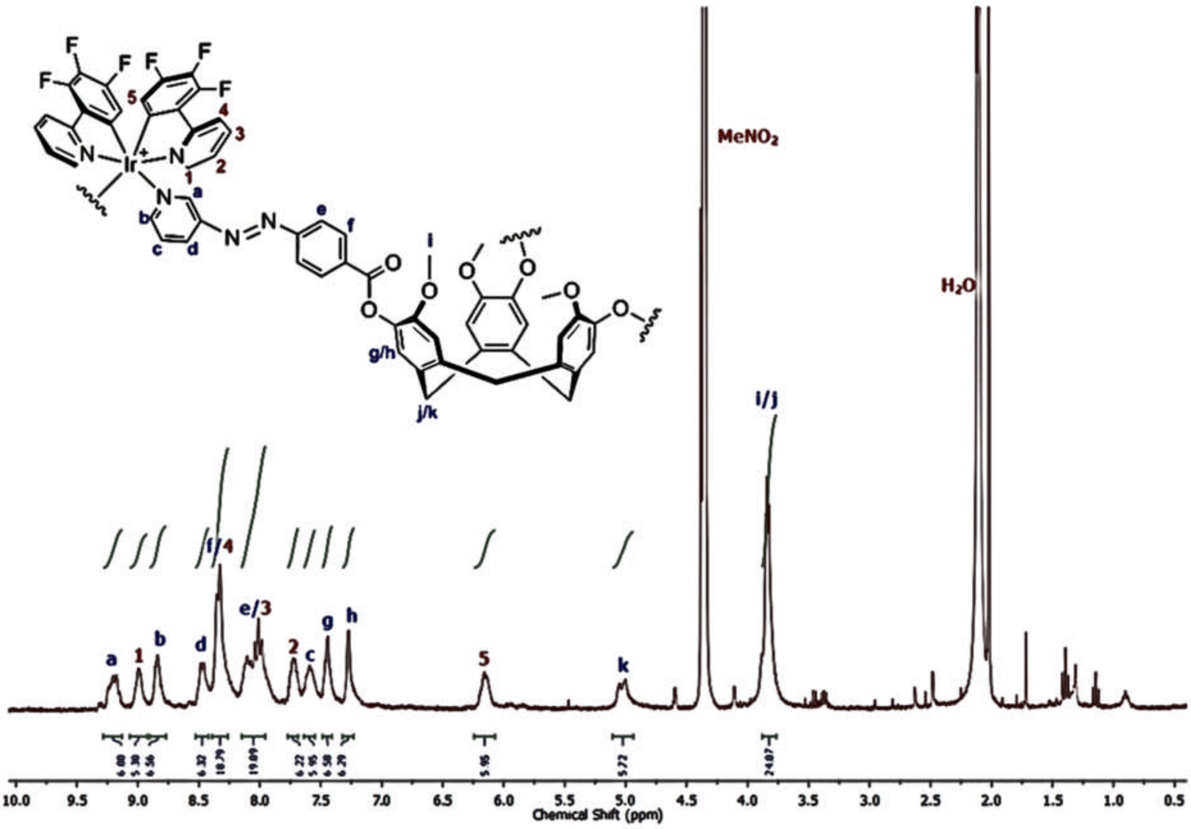

Fig. S64. ^{1}H NMR (300 MHz, $MeNO_2$-d^3) spectrum of **C1-F**.

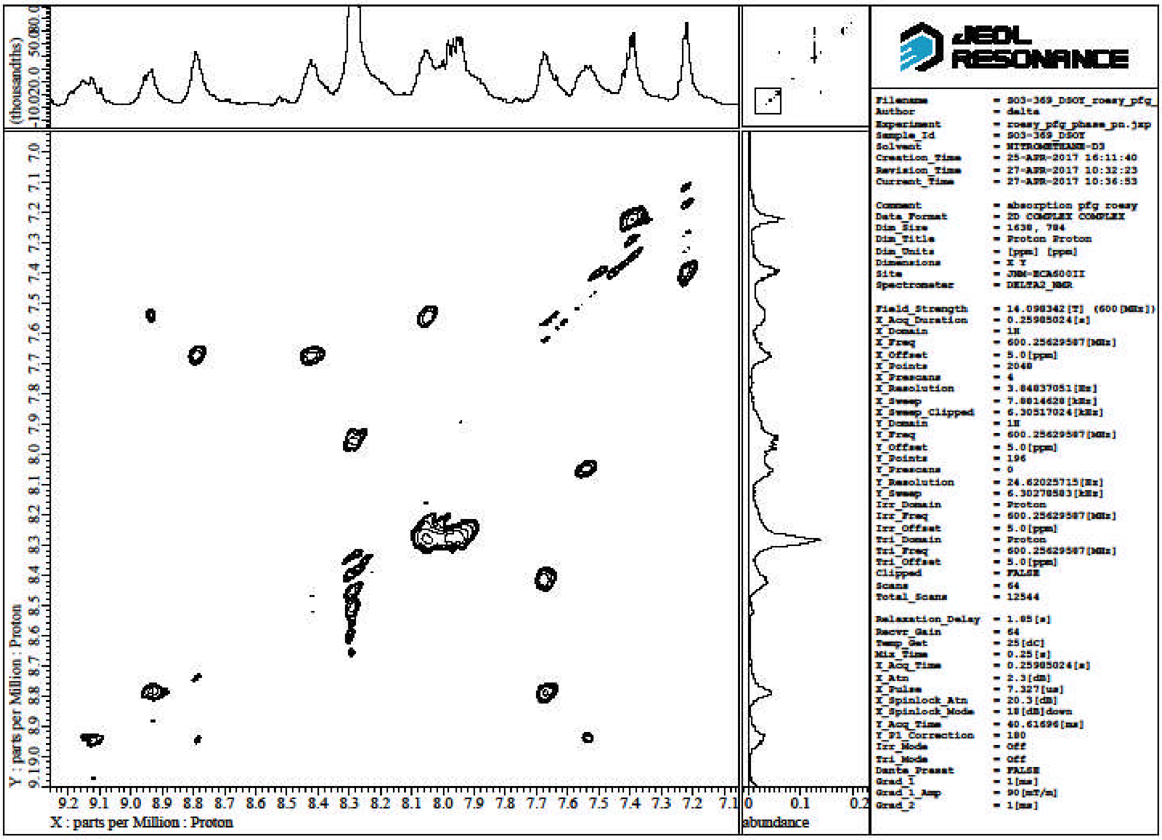

Fig. S65. ^{1}H ROESY NMR (600 MHz, $MeNO_2$-d^3) spectrum of **C1-F**.

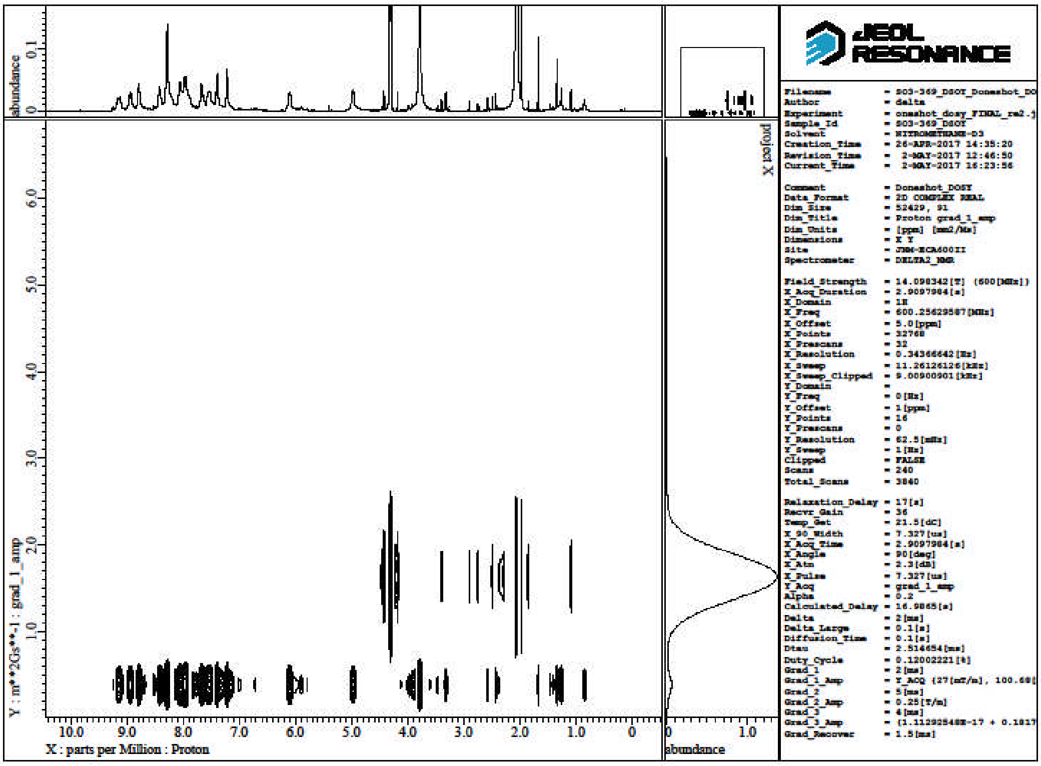

Fig. S66. ^{1}H DOSY NMR (600 MHz, $MeNO_2$-d^3) spectrum of **C1-F**.

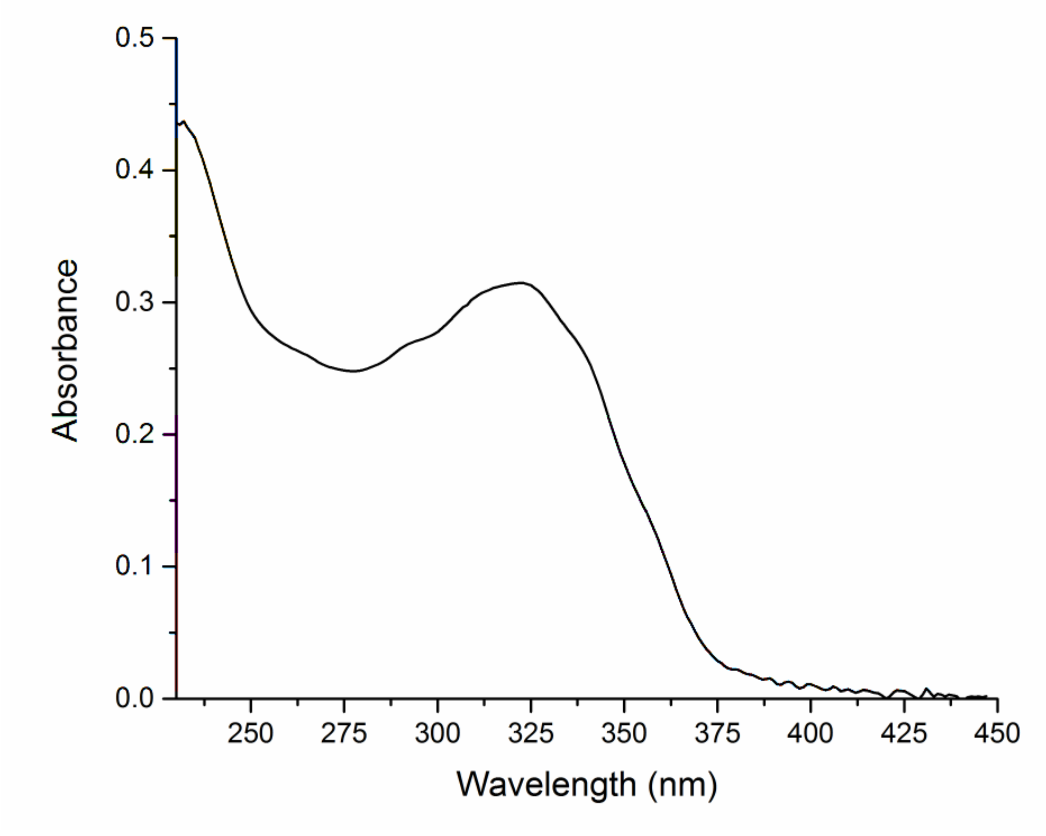

Fig. S67. UV-visible spectrum of cage **C1-F** in CH_2Cl_2.

[{Ir(ppy)$_2$}$_3$ (L3)$_2$]$^{3+}\cdot$3PF$_6^-$ coordination cage (C2)

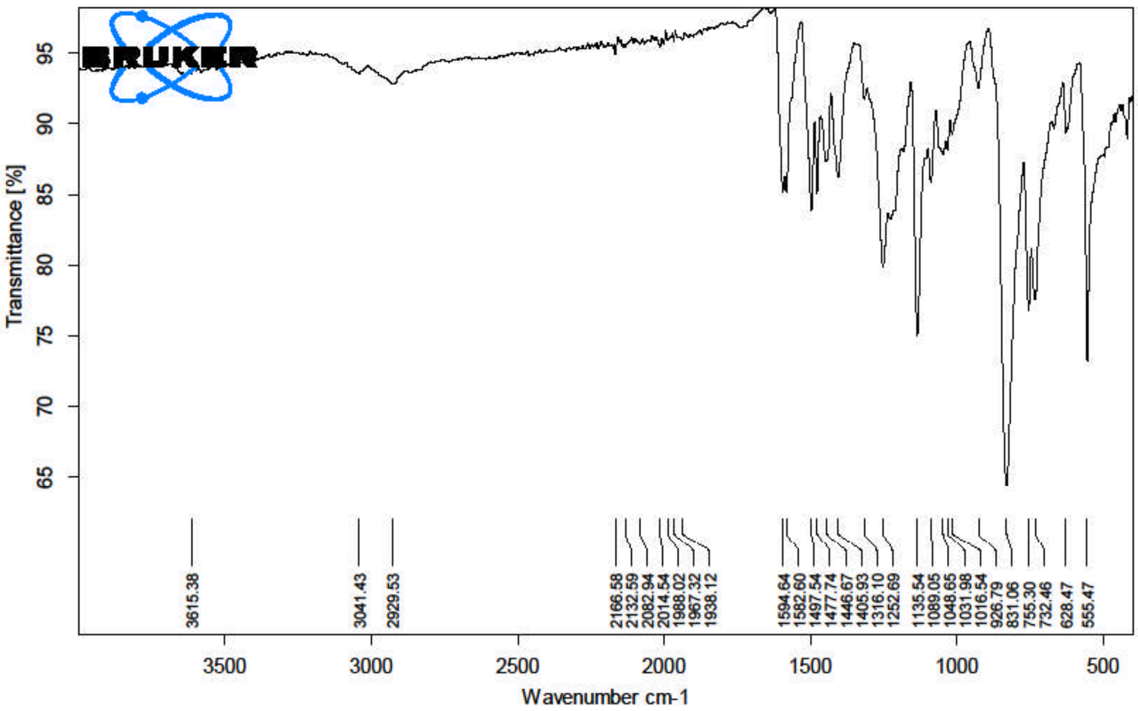

Fig. S68. Infrared spectrum of **C2**.

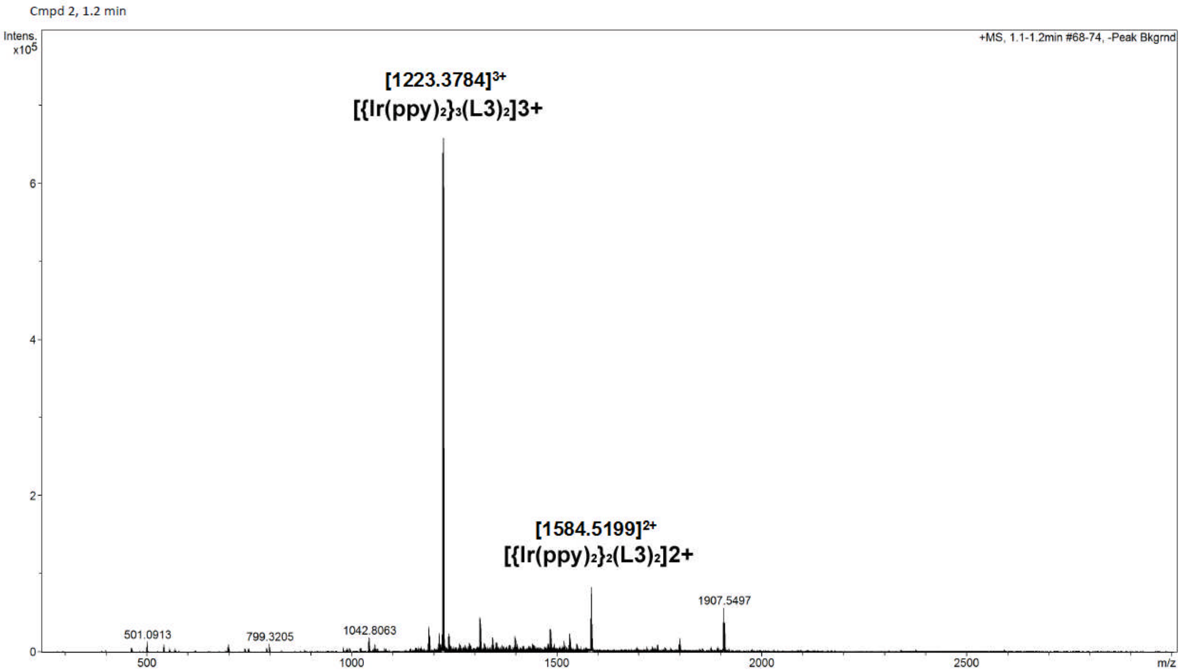

Fig. S69. Mass spectrum of **C2** in MeNO$_2$ after 24hrs equilibration.

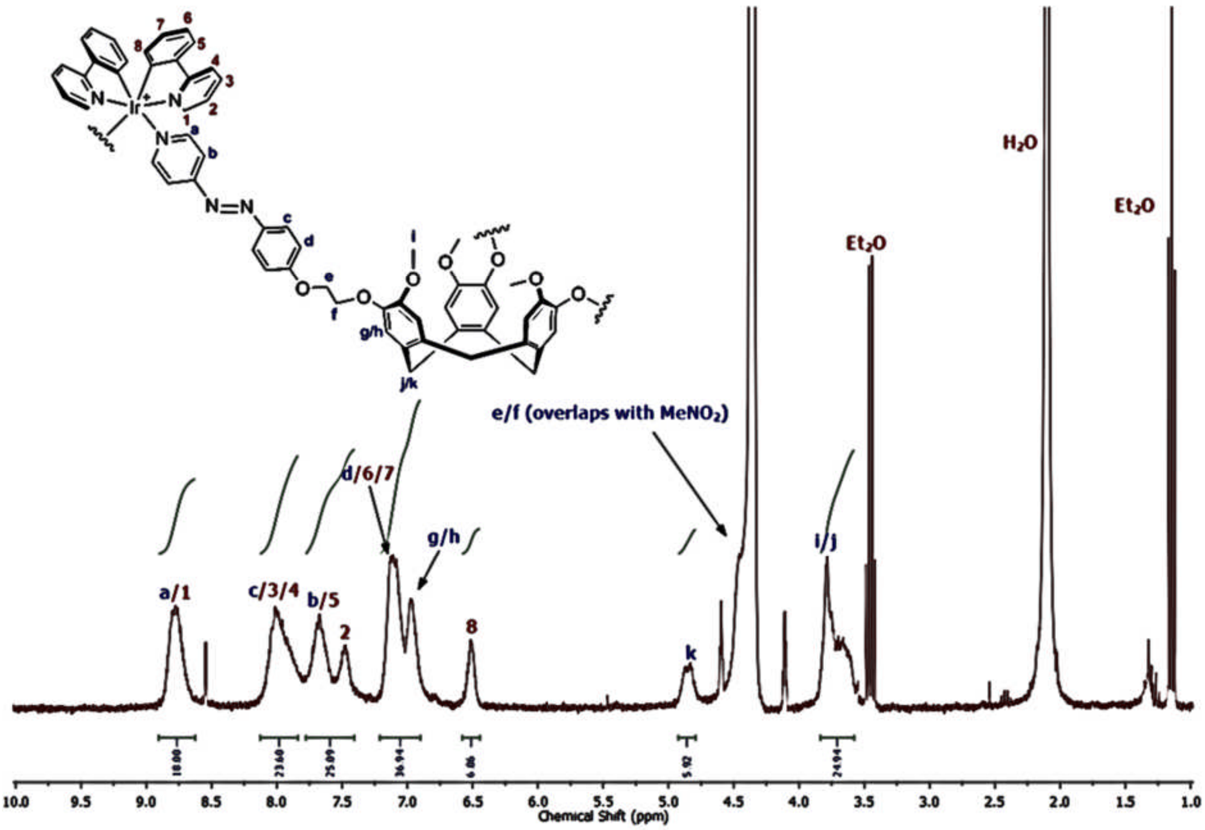

Fig. S70. ^{1}H NMR (300 MHz, MeNO$_2$-d^3) spectrum of **C2**.

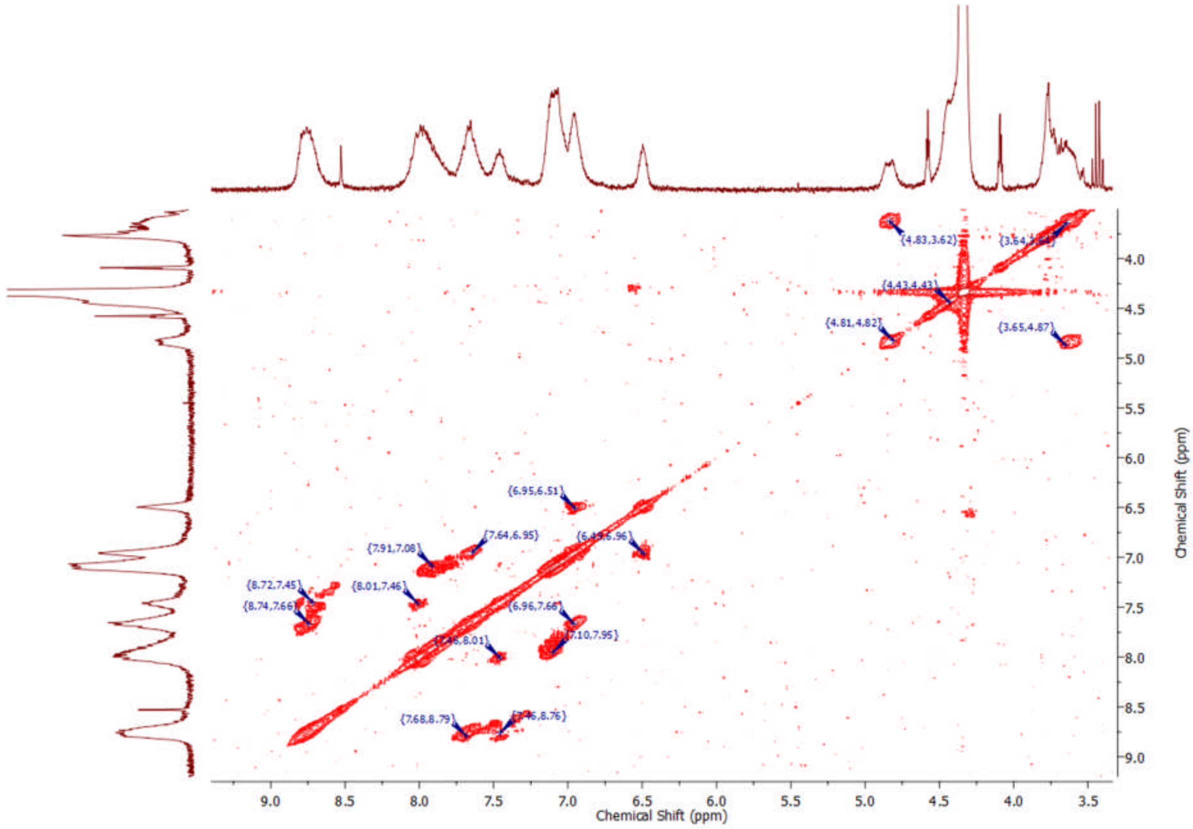

Fig. S71. ^{1}H-^{1}H COSY NMR (400 MHz, MeNO$_2$-d^3) of **C2**.

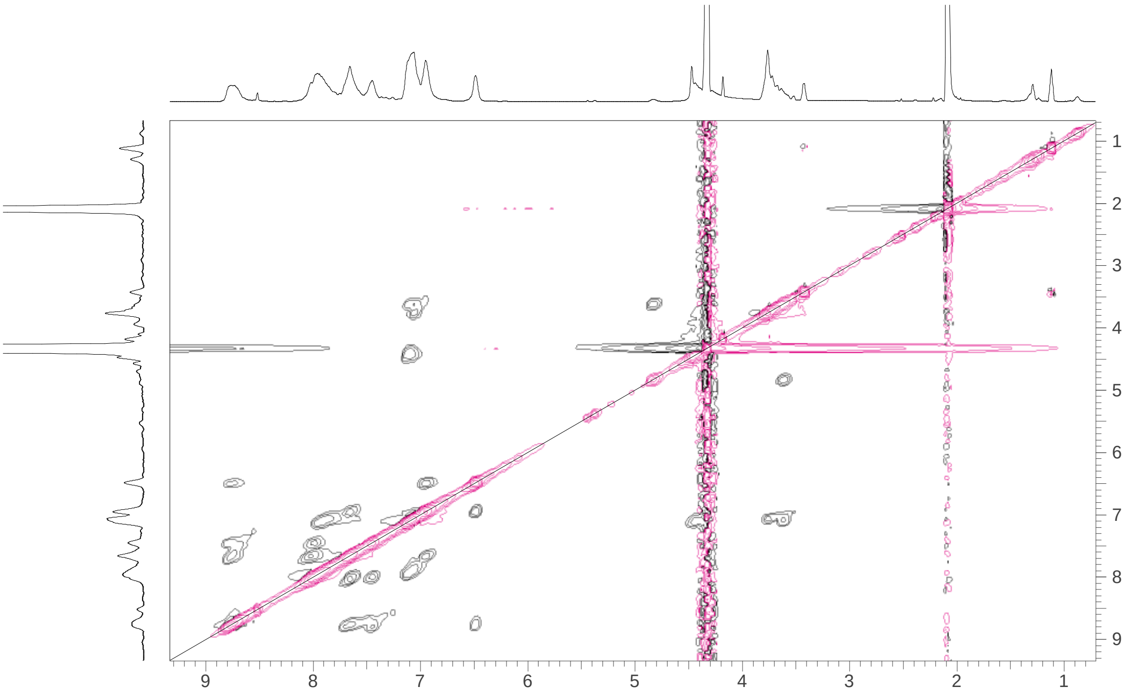

Fig. S72. ^{1}H ROESY NMR (500 MHz, $MeNO_2$-d^3) spectrum of **C2**.

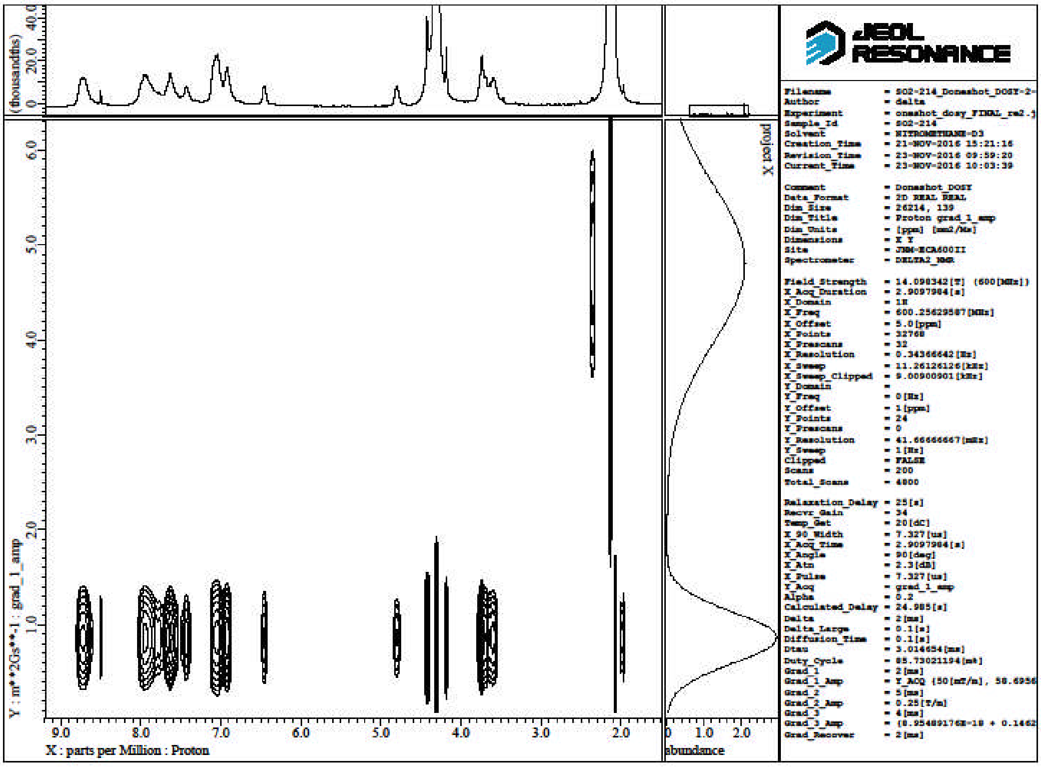

Fig. S73. ^{1}H DOSY NMR (600 MHz, $MeNO_2$-d^3) spectrum of **C2**.

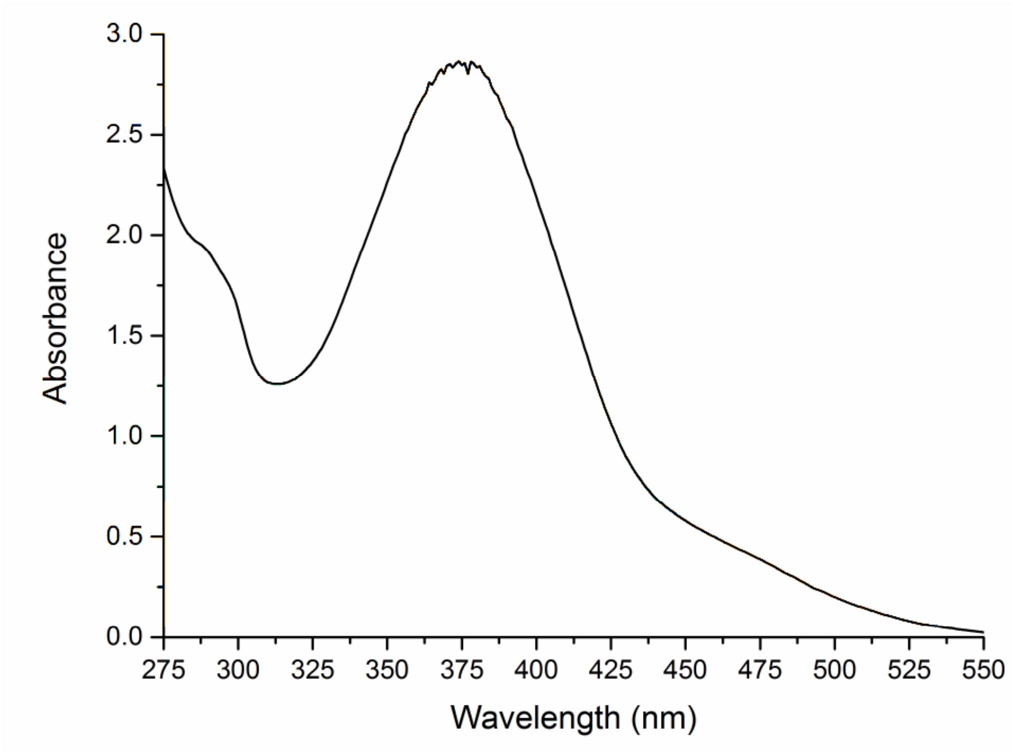

Fig. S74. UV-visible spectrum of cage **C2** in CH_2Cl_2.

[{Ir(ppy)$_2$}$_3$ (L4)$_2$]$^{3+}\cdot$3PF$_6^-$ coordination cage (C3)

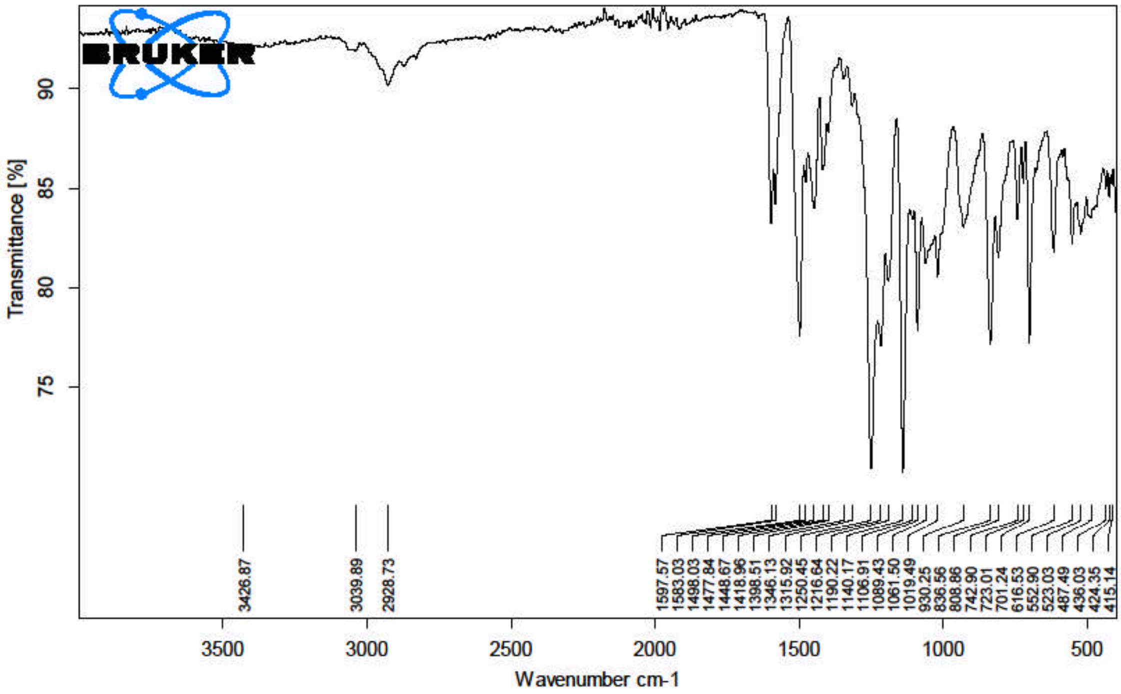

Fig. S75. Infrared spectrum of **C3**.

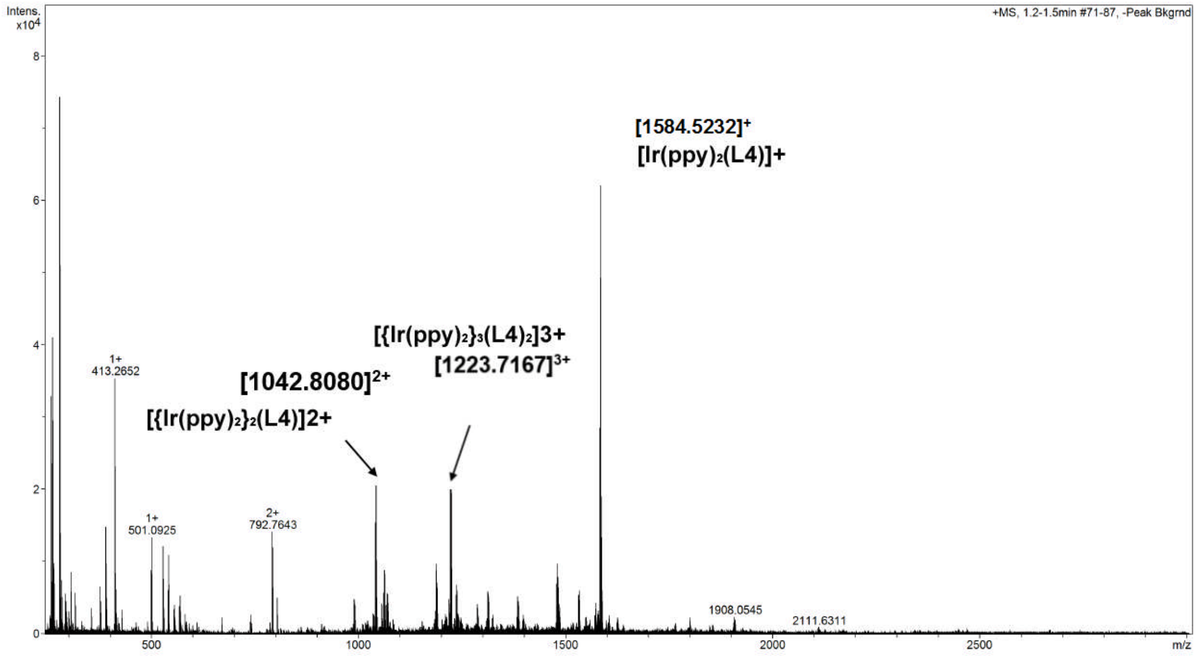

Fig. S76. Mass spectrum of **C3** in $MeNO_2$ after 24hrs equilibration. Complex **C3** shows notably more fragmentation in the MS than does other cages but DOSY NMR shows a single species in solution supporting the cage structure (DOSY is also very similar to that of **C2**).

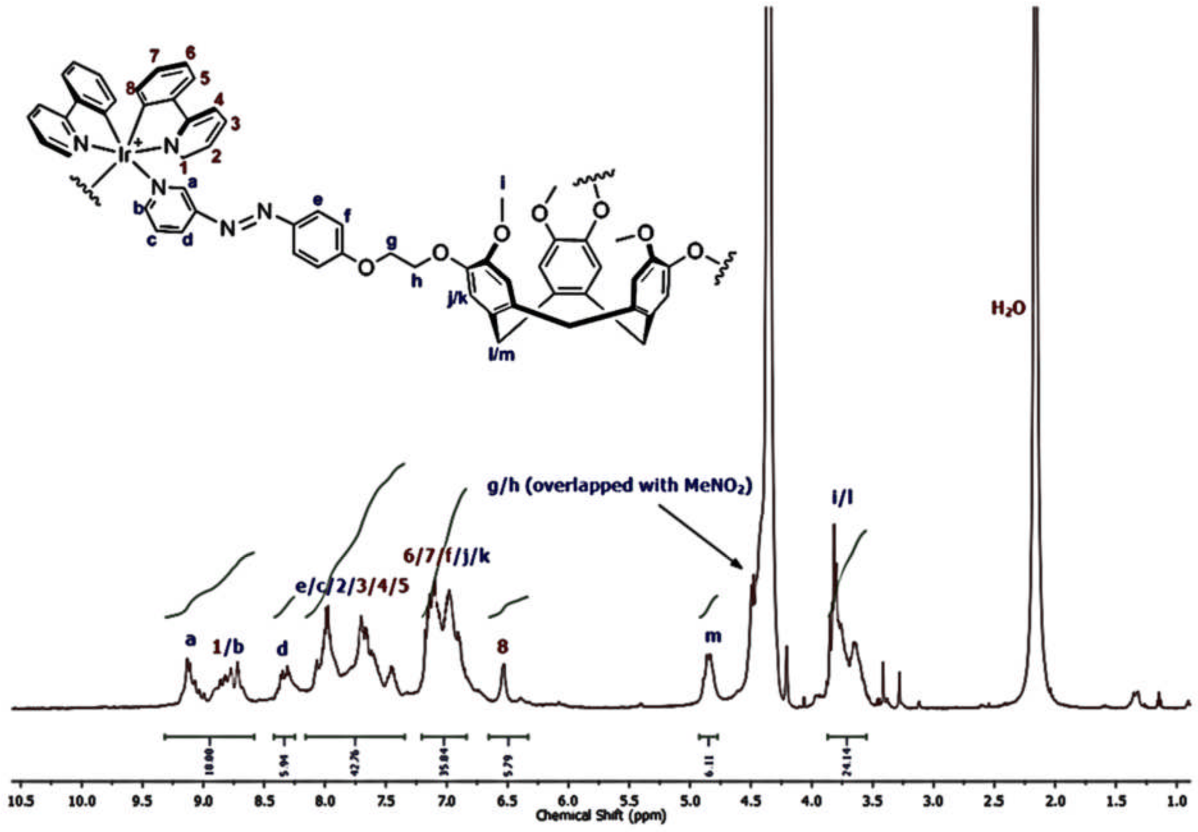

Fig. S77. ^{1}H NMR (300 MHz, $MeNO_2$-d^3) spectrum of **C3.**

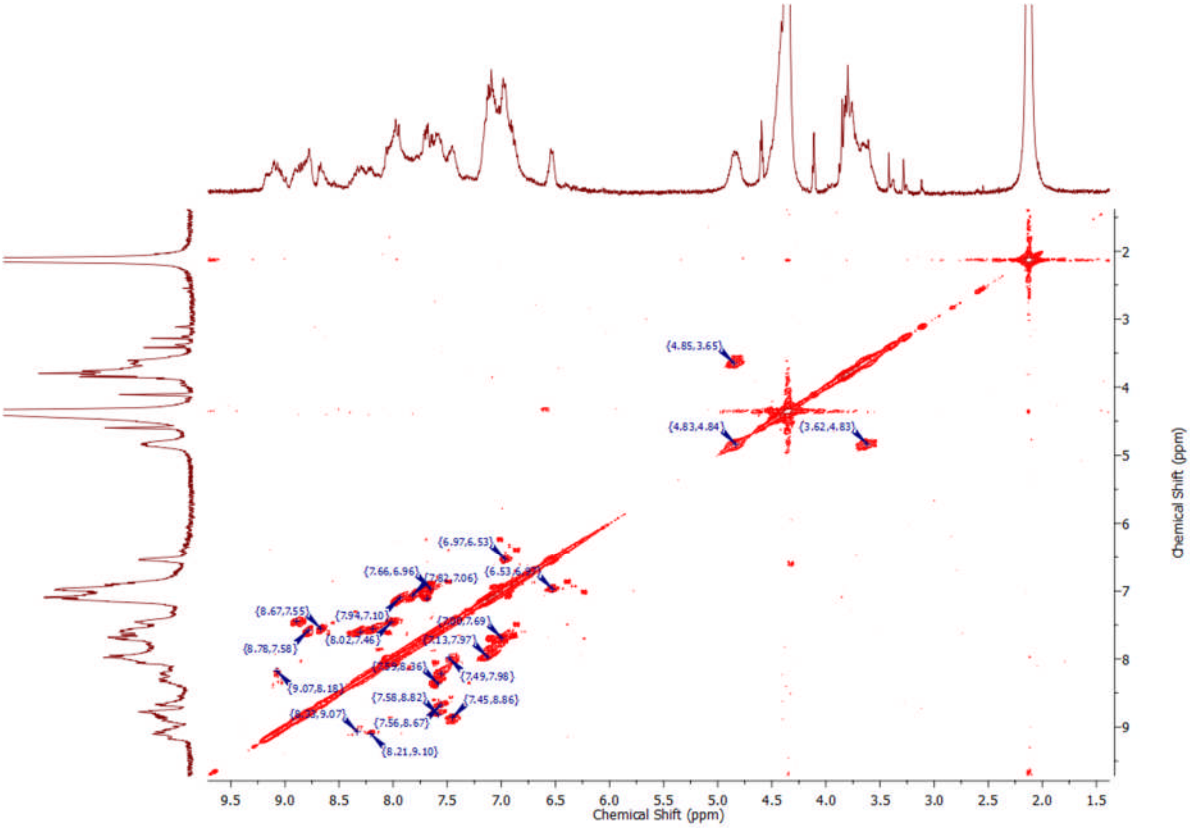

Fig. S78. ^{1}H-^{1}H COSY NMR (400 MHz, $MeNO_2$-d^3) of **C3**.

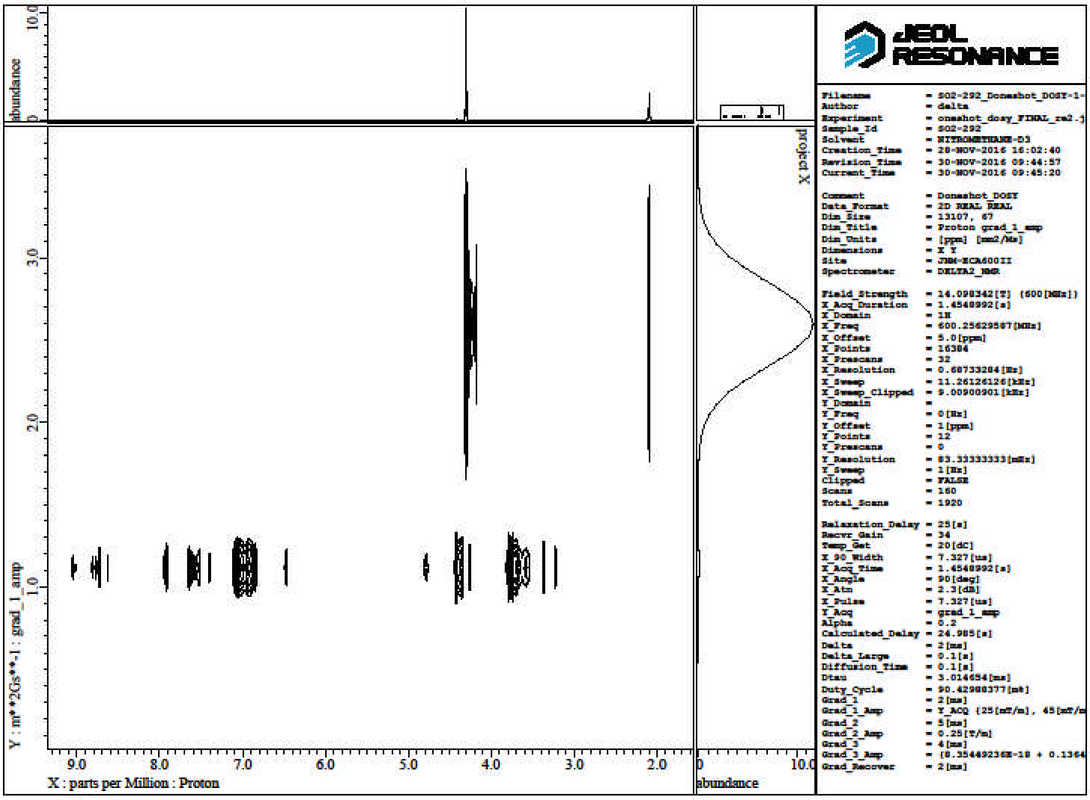

Fig. S79. ^{1}H DOSY NMR (600 MHz, $MeNO_2$-d^3) spectrum of **C3**.

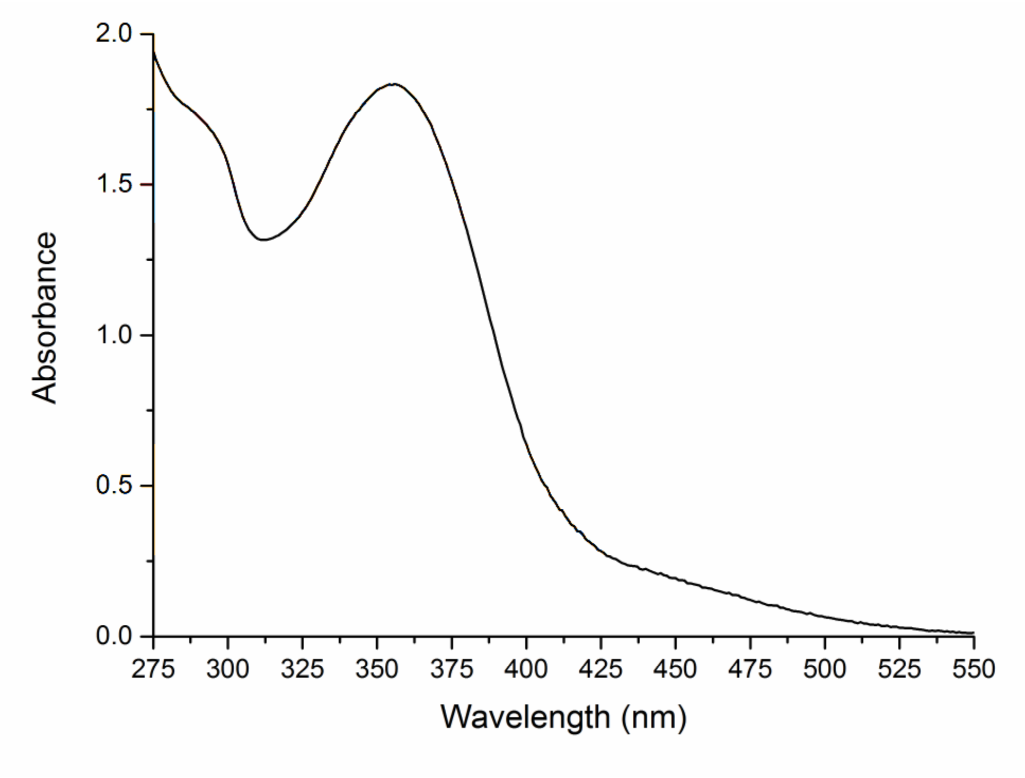

Fig. S80. UV-visible spectrum of cage **C3** in CH_2Cl_2.

3. Table and Spectra to support photoisomerisation experiments

Table S3. Conversion efficiency of photoswitching of ligands and coordination cages determined by UV-visible studies.

Compound	λ_1 (nm)	*E*→*Z* (%)	λ_2 (nm)	*Z*→*E* (%) [c]
**L2**	330	50[a] (78)[b]	450	100
**L3**	350	36[a]	450	91
**L4**	340	56[a] (73)[b]	450	100
**C1**	355	39[b]	450	94
**C1-Me**	355	35[b]	450	95
**C1-F**	355	26[b]	450	94
**C2**	355	16[b]	450	99
**C3**	355	40[b]	450	100

[a] after irradiation with Xe lamp at λ_1 for 45 mins for **L2-L4**; [b] after irradiation with Nd:YAG laser at λ_1 until photostationary state reached; [c] after subsequent irradiation at λ_2 with monochromated Xe lamp for 15 mins.

3.1 Ligand Photoisomerisation

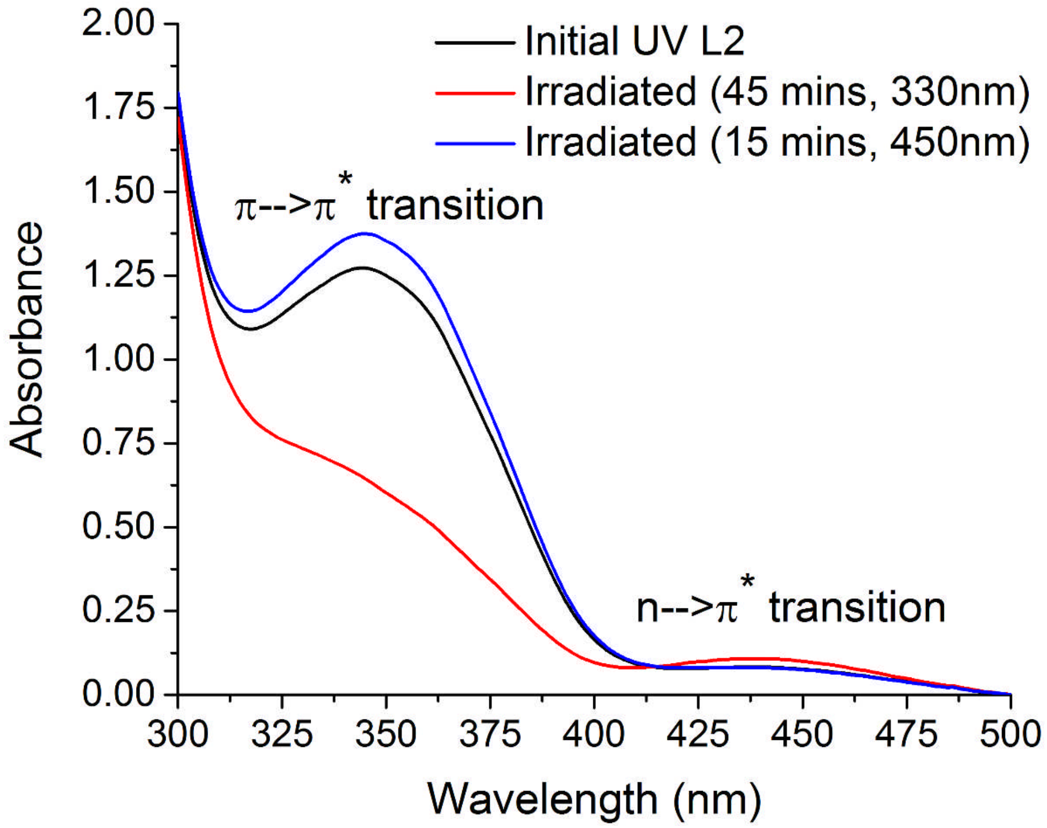

Fig.S81. UV-visible spectrum of **L2** in CD_2Cl_2 on irradiation with Xe lamp at 330 nm for 45 mins.

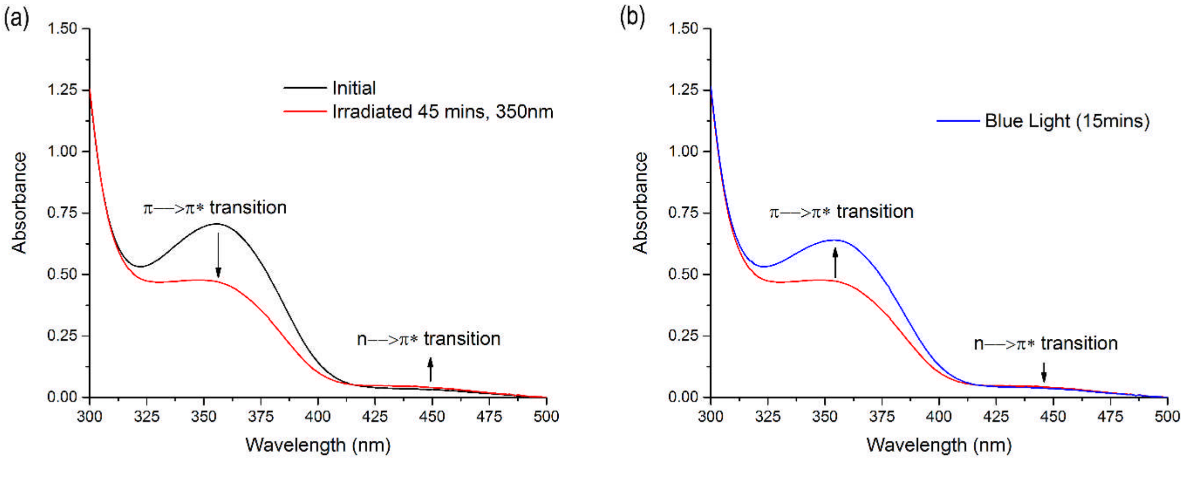

Fig. S82. UV-visible spectra of **L3** in DMSO. (a) black trace initial spectrum, red trace after irradiation with Xe lamp at 350 nm for 45 minutes; (b) blue trace after irradiation at 450 nm for 15 mins.

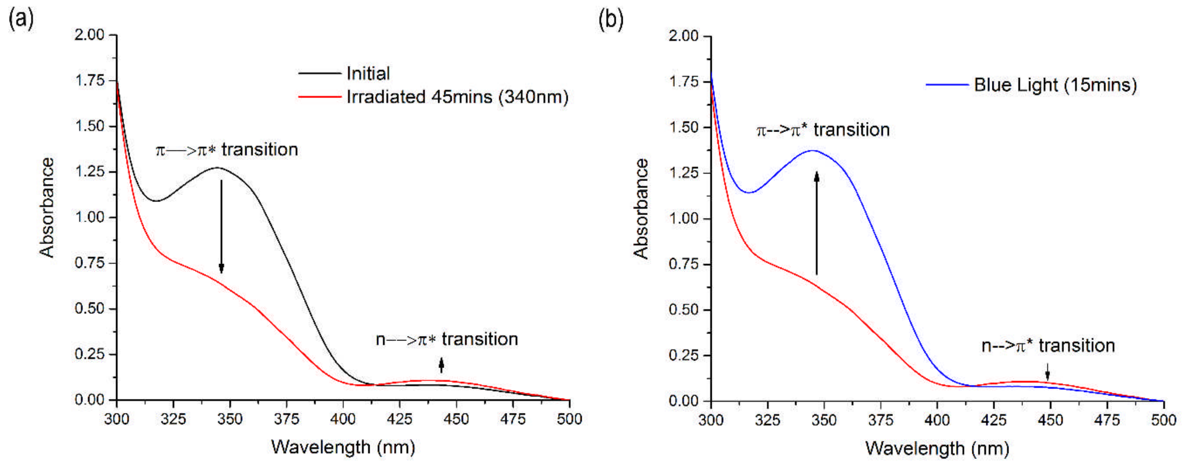

Fig. S83. UV-visible spectra of **L4** in DMSO (a) black trace initial spectrum, red trace after irradiation with Xe lamp at 340 nm for 45 minutes, (b) blue trace after irradiation at 450 nm for 15 mins.

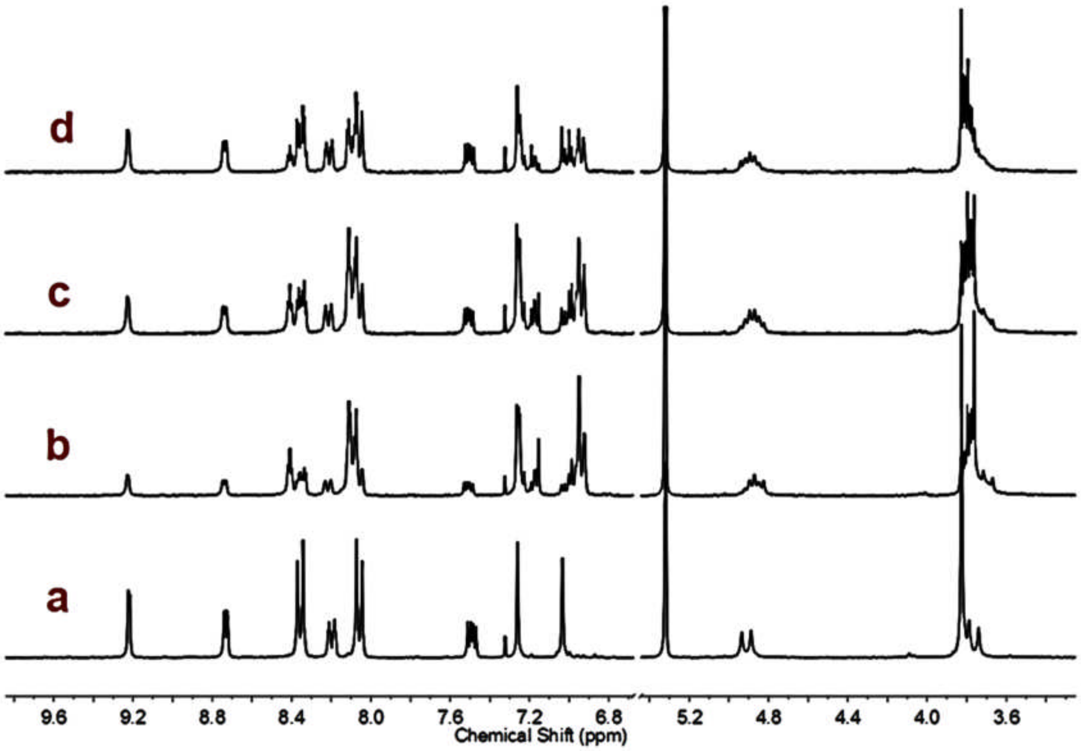

Fig. S84. ^{1}H NMR study showing stability of Z-rich photostationary state of **L2** in CD_2Cl_2: a. initial all *E* isomer; b. After irradiation for 900s with 355 nm Nd:YAG laser; c. Photostationary state after 24 hours in the dark; d. Photostationary state after 48 hours in the dark. The region between 6.7 and 5.3 ppm has been omitted for clarity.

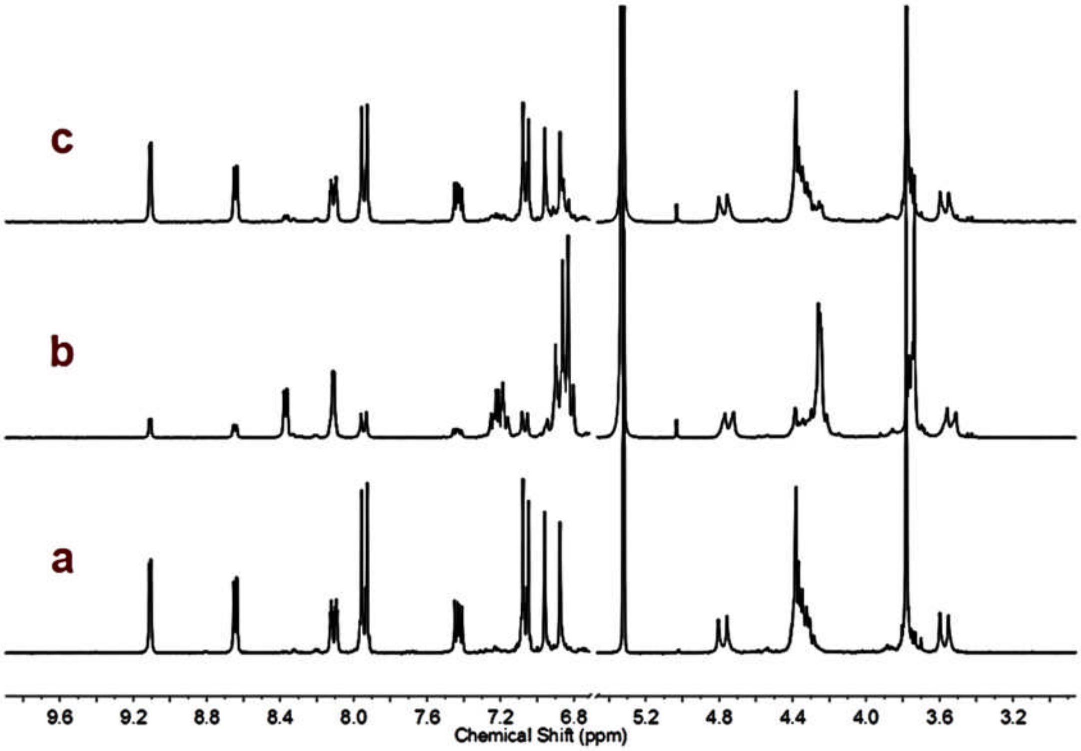

Fig. S85. ^{1}H NMR study of thermal relaxation of ligand **L4** in CD_2Cl_2 showing; **a.** initial all *E* isomer; **b.** After irradiation for 900s with 355 nm Nd:YAG laser; **c.** Photostationary state after 24 hours in the dark. The region between 6.8 and 5.3 ppm has been omitted for clarity.

3.2 Coordination cage and complex Photoisomerisations

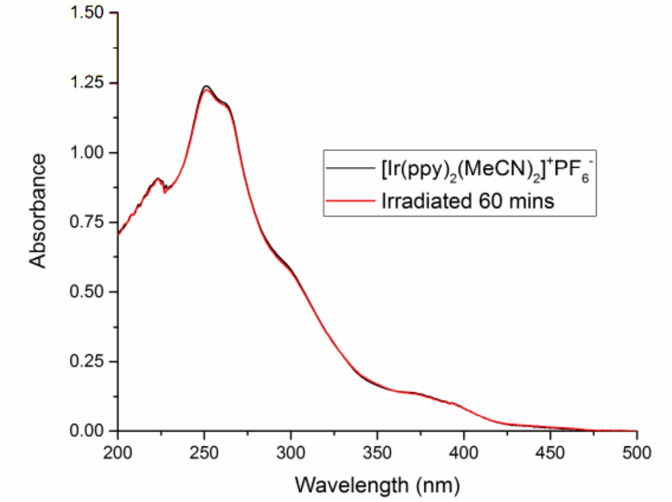

Fig S86. UV-visible spectra of $[Ir(ppy)_2(NCMe)_2].PF_6$ before and after irradiation at 330 nm with Xe lamp.

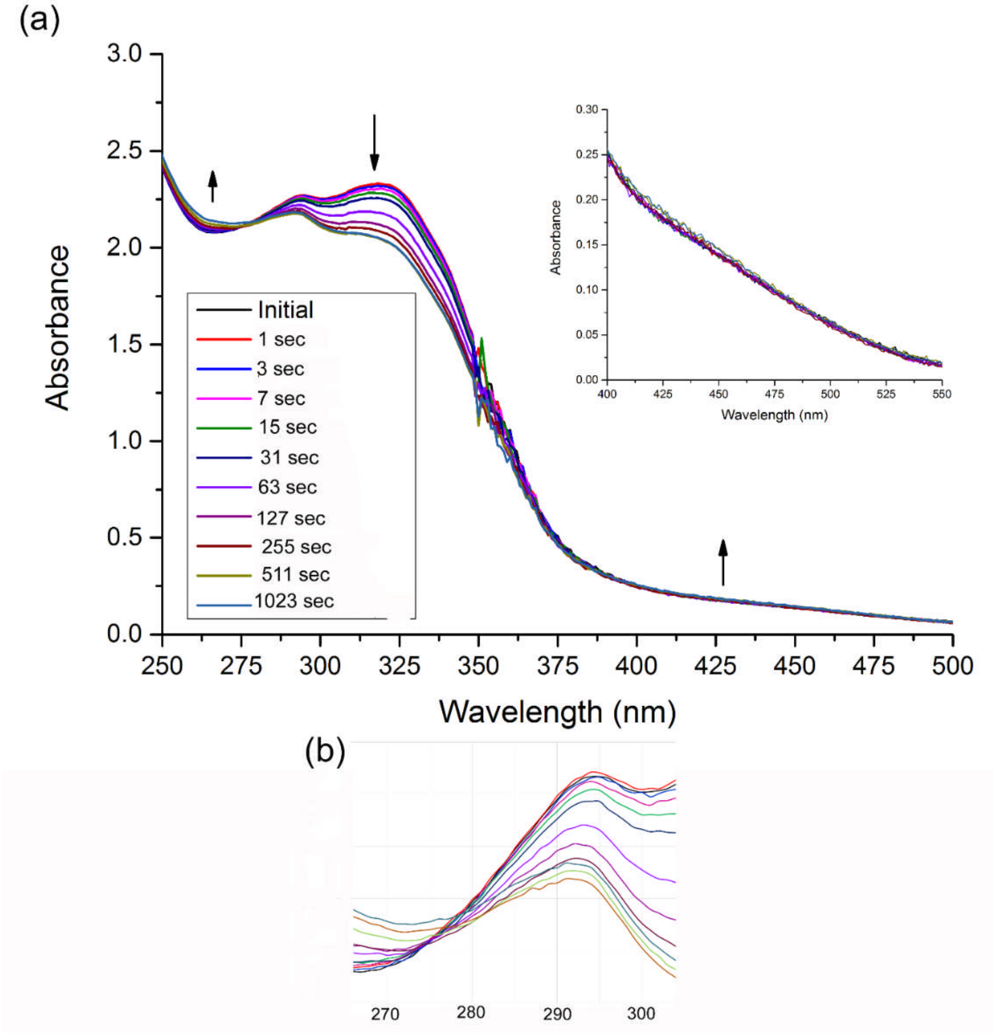

Fig. S87. UV-visible spectra showing *E*→*Z* isomerisation of cage **C2** on irradiation of DCM solution with 355 nm laser. Inset shows growth of n→π* transition. (Noise ca. 350 nm is an artefact of instrument set-up). (b) expansion illustrating apparent isosbestic point is not genuine.

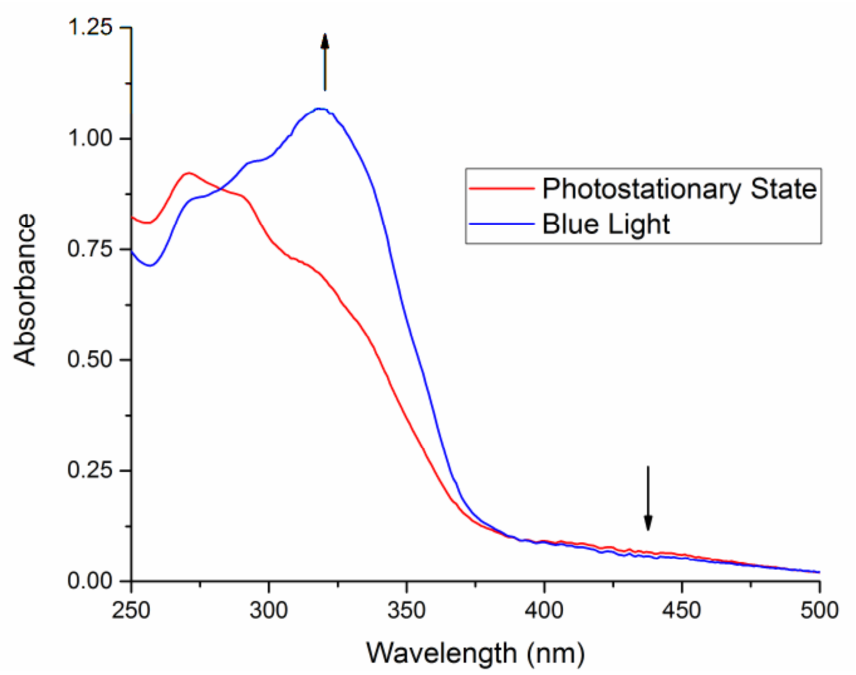

Fig. S88. UV-visible spectra showing reverse $Z \rightarrow E$ isomerisation of cage **C1** on irradiation of DCM solution with Xe lamp at 450 nm for 15 mins, (subsequent to reaching photostationary state from irradiation at 355 nm).

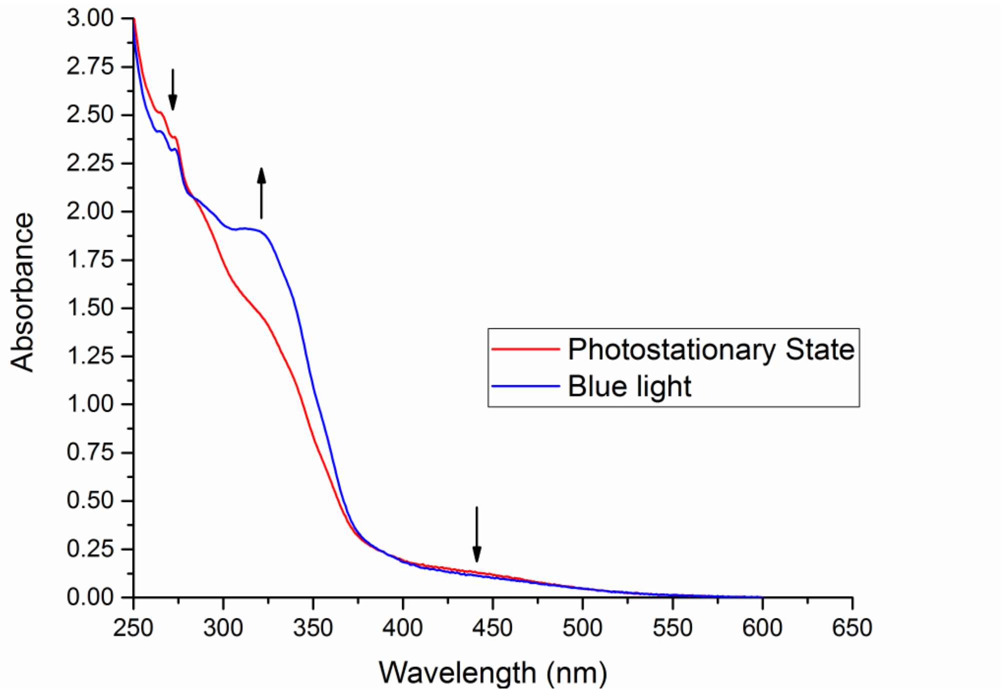

Fig. S89. UV-visible spectra showing reverse $Z \rightarrow E$ isomerisation of cage **C1-Me** on irradiation of DCM solution with Xe lamp at 450 nm for 15 mins, (subsequent to reaching photostationary state from irradiation at 355 nm).

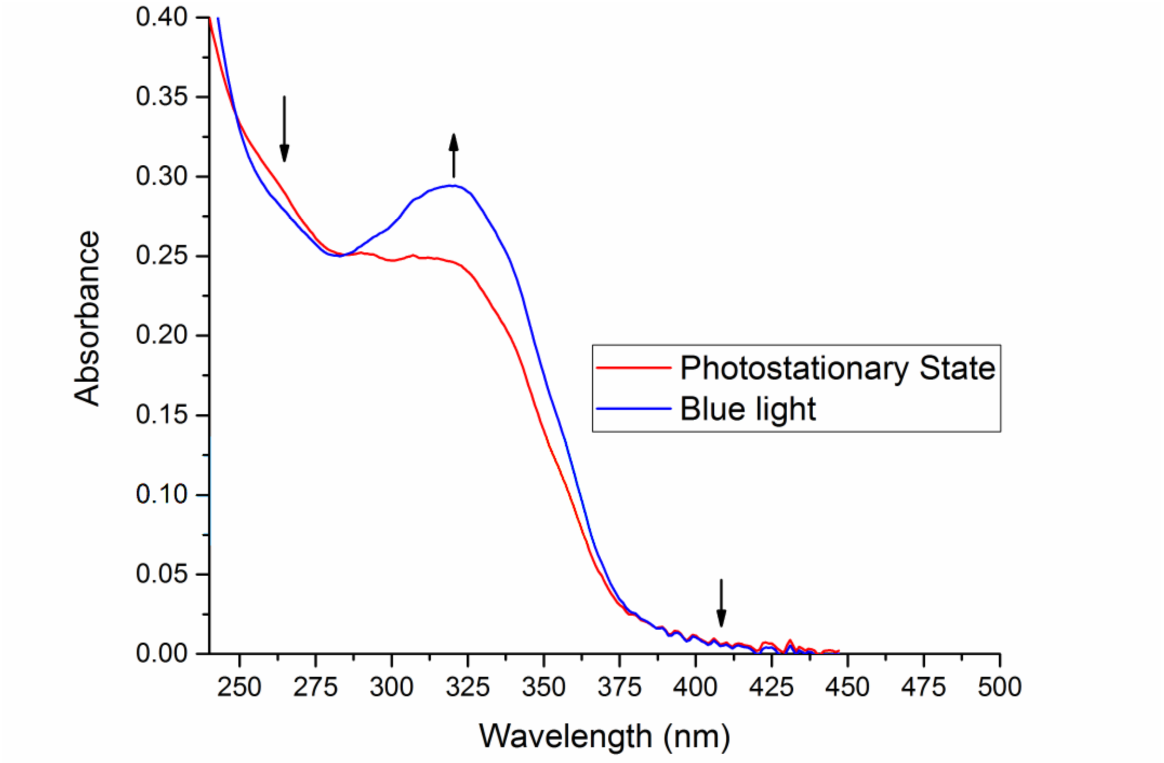

Fig. S90. UV-visible spectra showing reverse $Z \rightarrow E$ isomerisation of cage **C1-F** on irradiation of DCM solution with Xe lamp at 450 nm for 15 mins, (subsequent to reaching photostationary state from irradiation at 355 nm).

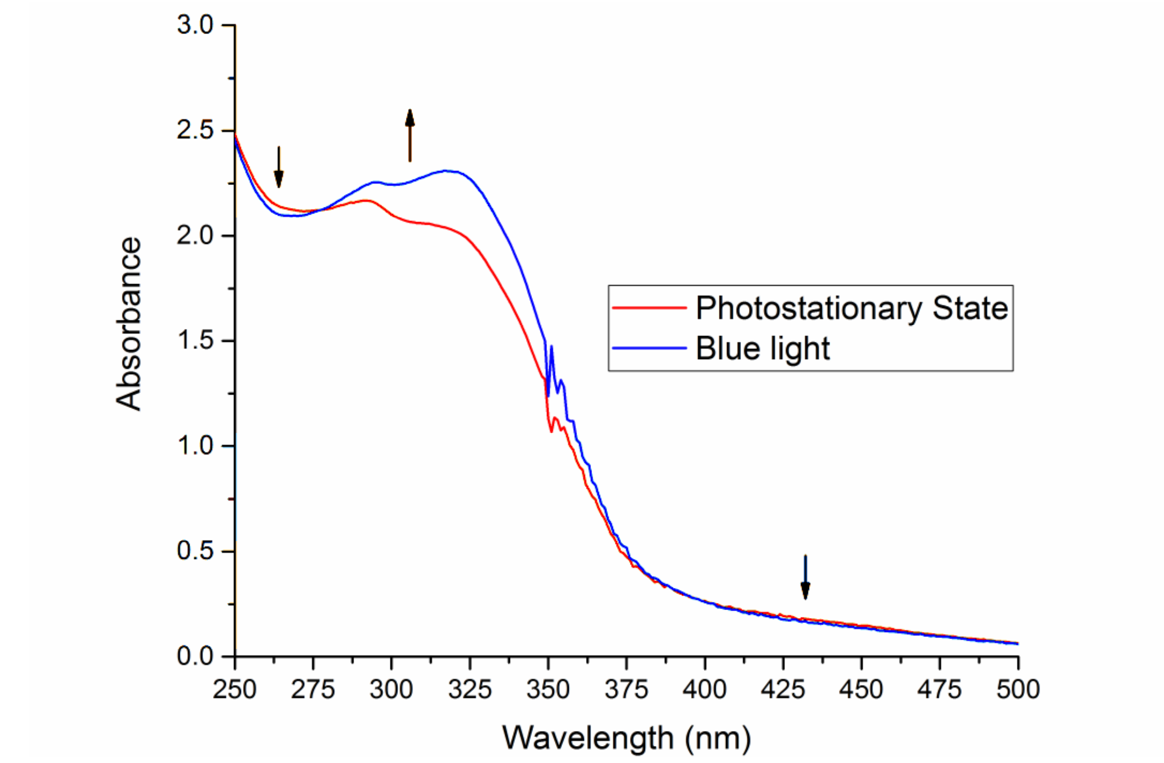

Fig. S91. UV-visible spectra showing reverse $Z \rightarrow E$ isomerisation of cage **C2** on irradiation of DCM solution with Xe lamp at 450 nm for 15 mins, (subsequent to reaching photostationary state from irradiation at 355 nm). (Noise ca. 350 nm is an artefact of instrument set-up).

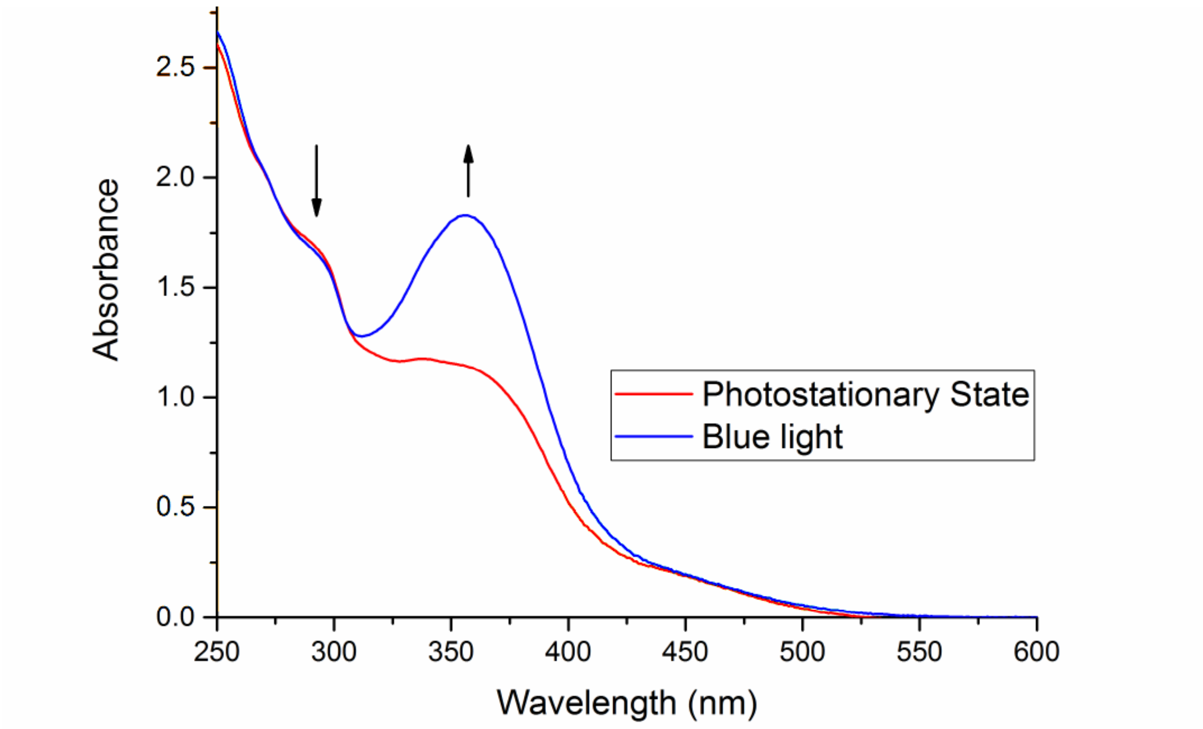

Fig. S92. UV-visible spectra showing reverse *Z*→*E* isomerisation of cage **C3** on irradiation of DCM solution with Xe lamp at 450 nm for 15 mins, (subsequent to reaching photostationary state from irradiation at 355 nm).

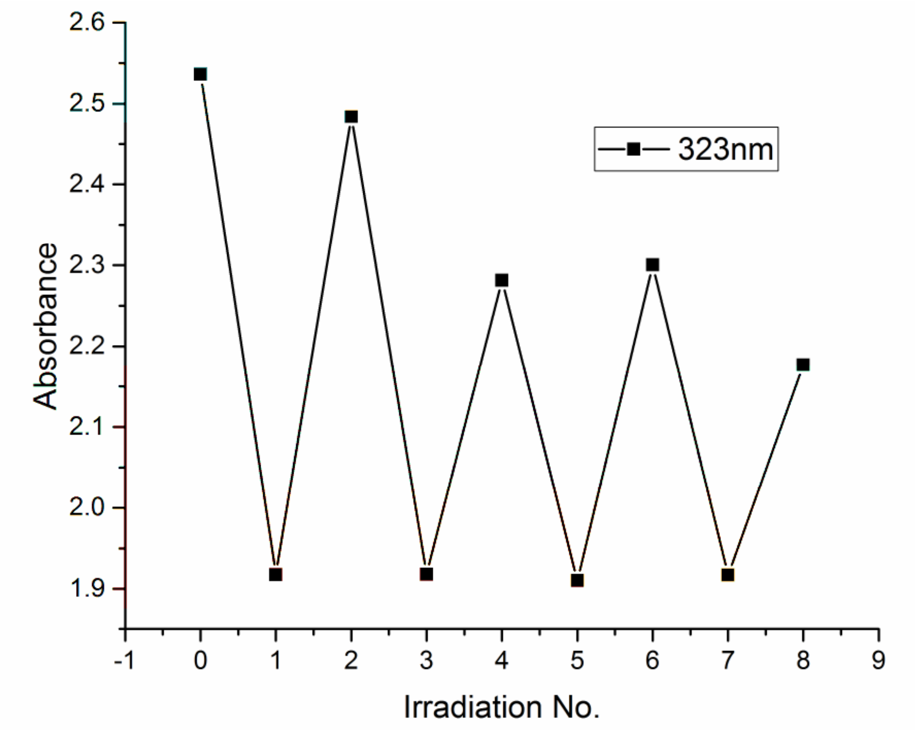

Fig. S93. Plot of absorbance at 323 nm of solution of **C1-Me** in CH_2Cl_2 on cycling of irradiation, odd number irradiations show *E*→*Z* isomerisation at 355 nm, while even numbers are reverse *Z*→*E* isomerisation at 450 nm.

4. Spectra and images supporting emission studies

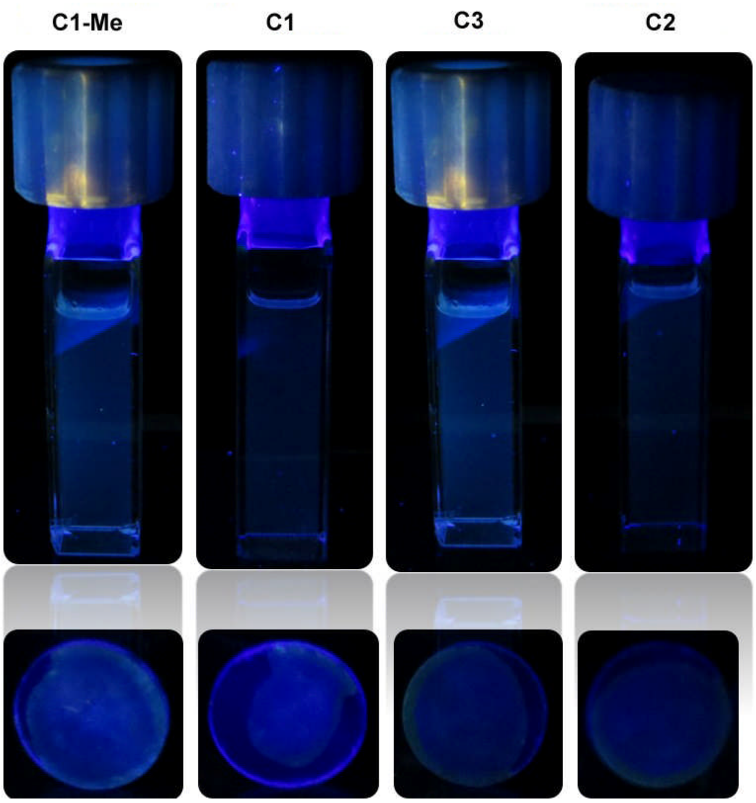

Fig. S94. Dark-room images of luminescence of metallo-cages in DCM solution (upper image) and in PMMA matrix (lower image).

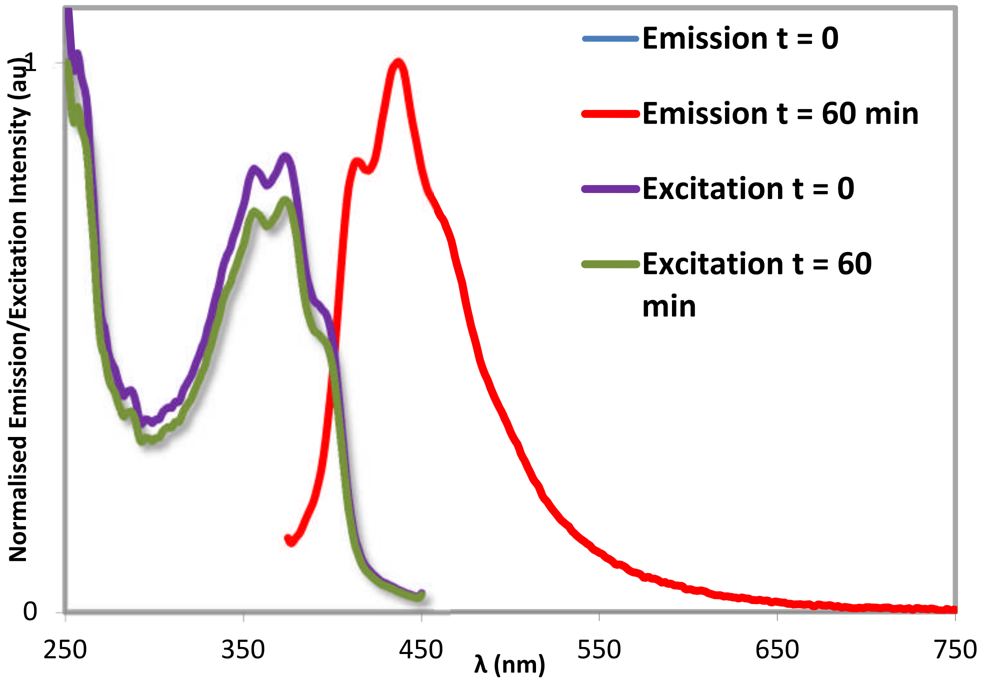

Fig. S95. Emissions and excitation spectra of **C1** in DCM solution after photoexcitation at λ_{exe} = 378 nm and time, t = 0 and 60 min.

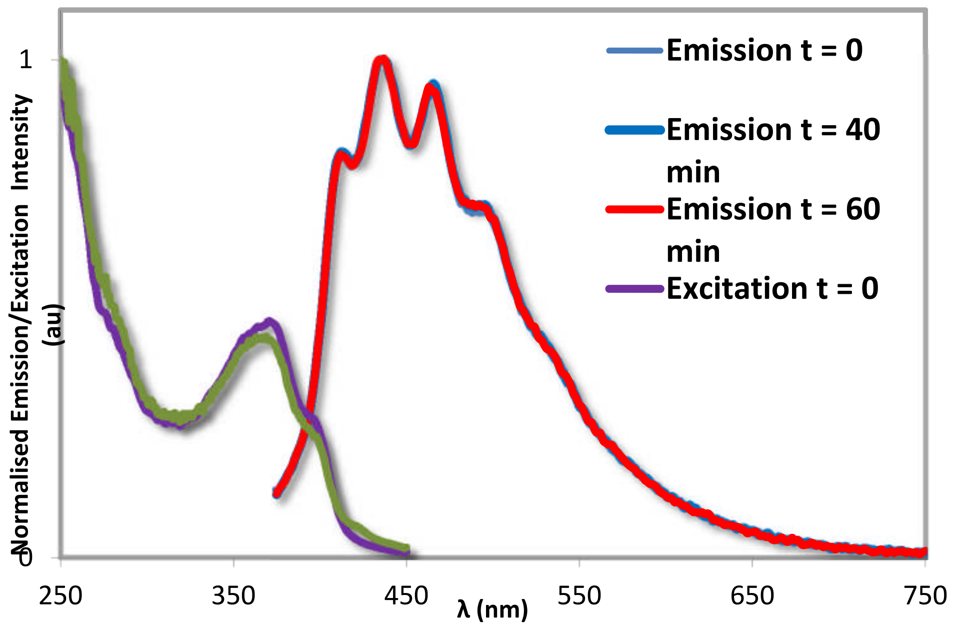

Fig. S96. Emissions and excitation spectra of **C1-Me** in DCM solution after photoexcitation at λ_{exe} = 378 nm.

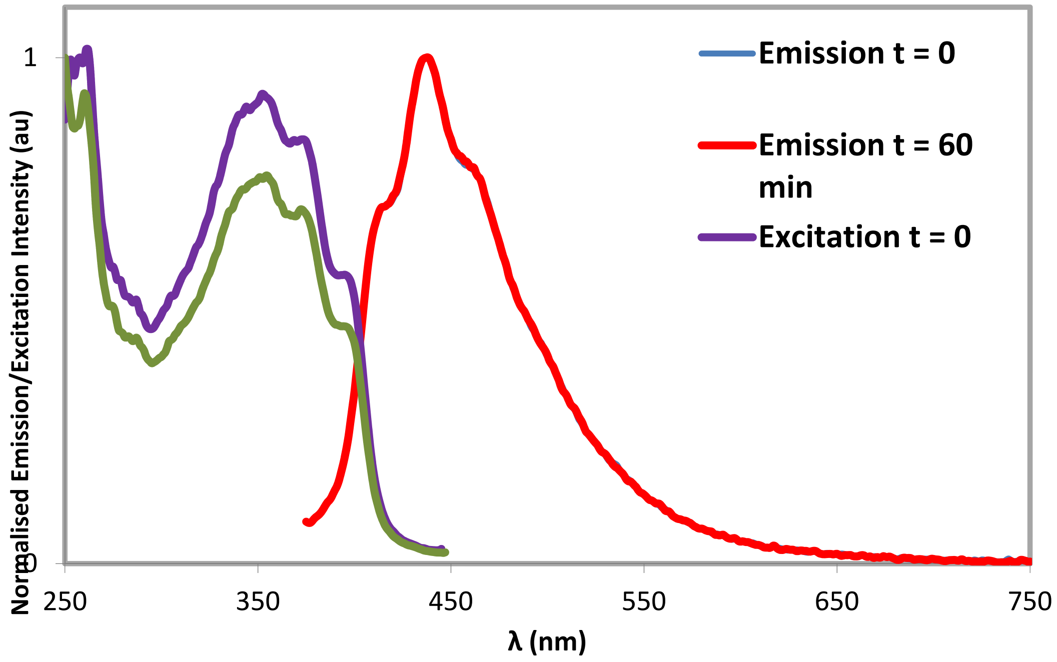

Fig. S97. Emissions and excitation spectra of **C2** in DCM solution after photoexcitation at λ_{exe} = 378 nm.

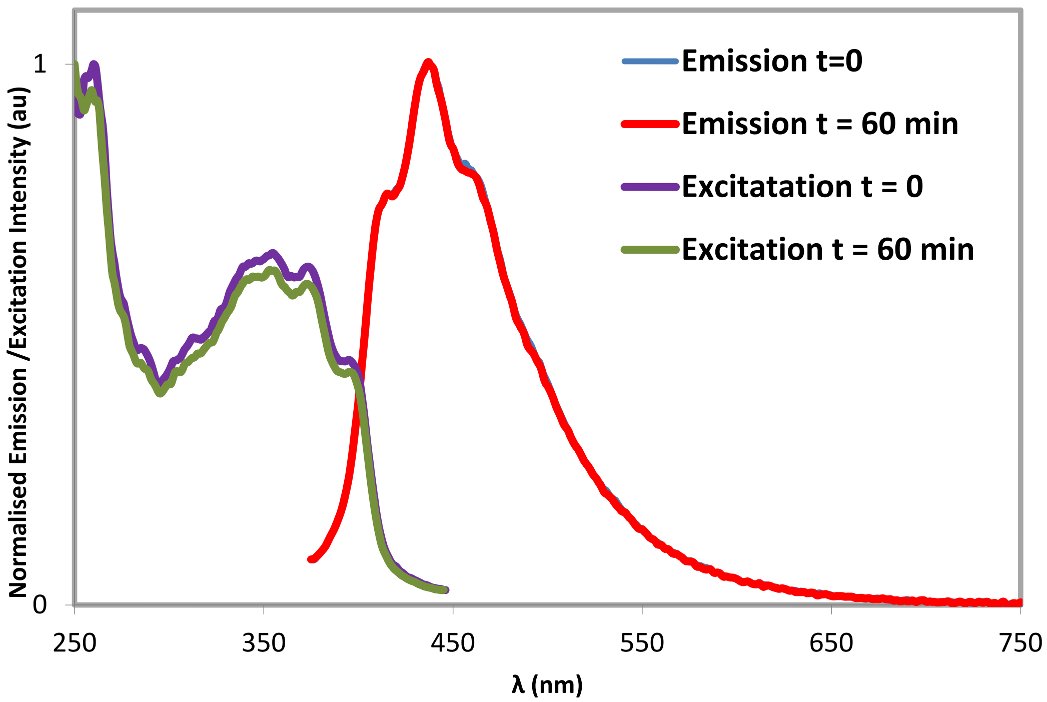

Fig. S98. Emissions and excitation spectra of **C3** in DCM solution after photoexcitation at λ_{exe} = 378 nm.

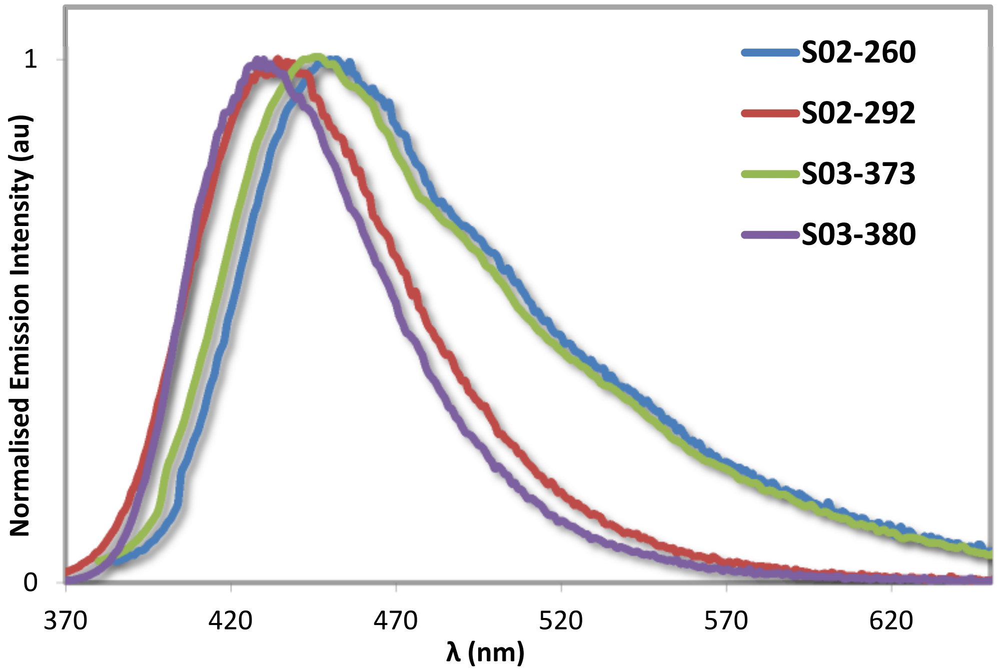

Fig. S99. Photoluminescence emission spectra of PMMA-films doped with metallo-cages. **C1** = SO2-260; **C1-Me** = SO2-373; **C2** = SO3-380; **C3** = SO2-292.

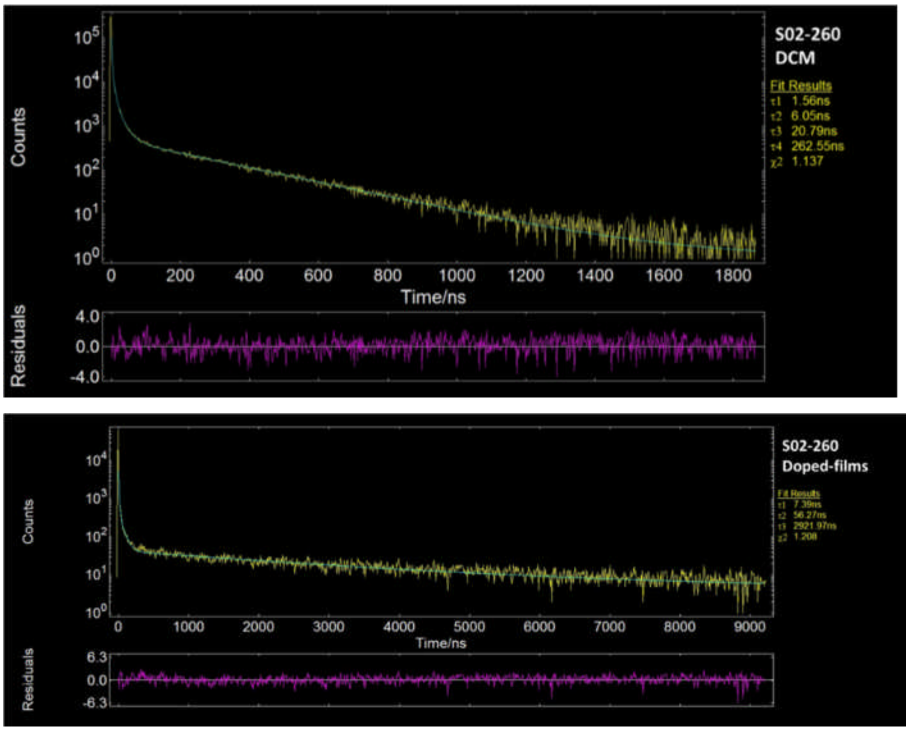

Fig. S100. Luminescence lifetime data for **C1** in DCM solution (upper) and doped PMMA-film (lower).

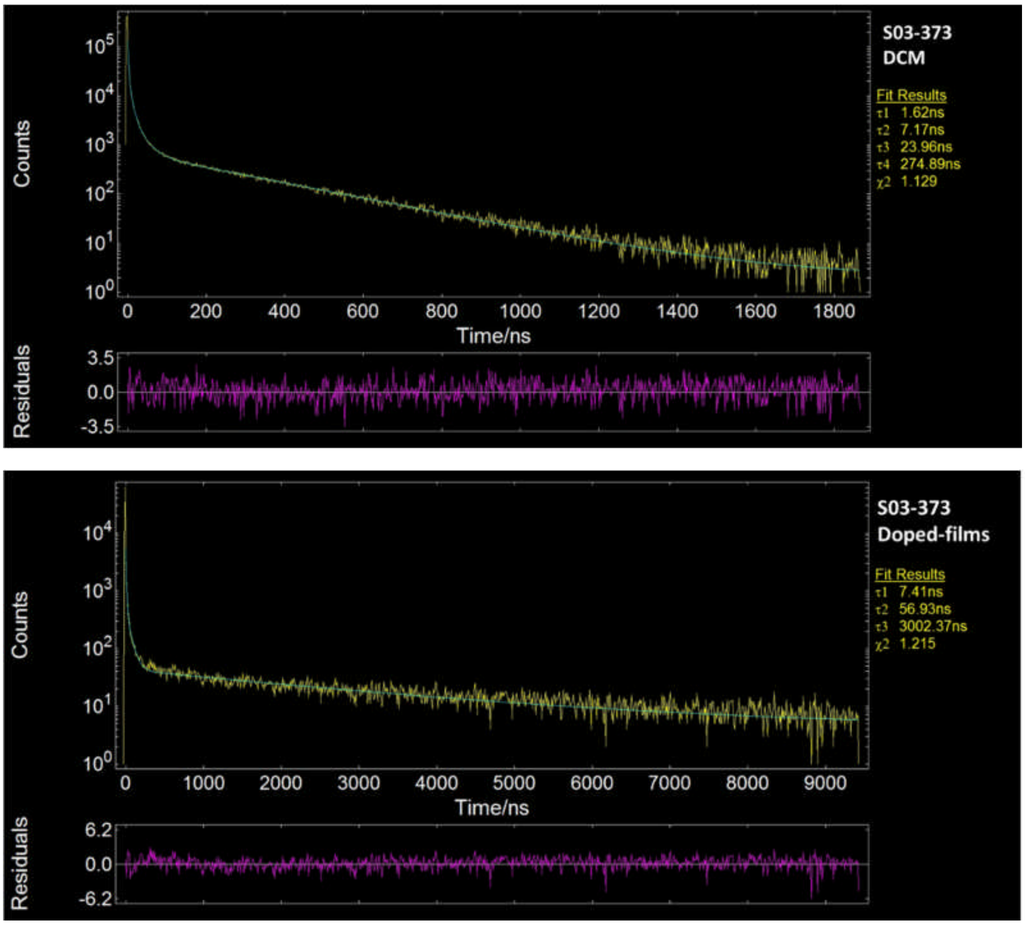

Fig. S101. Luminescence lifetime data for **C1-Me** in DCM solution (upper) and doped PMMA-film (lower).

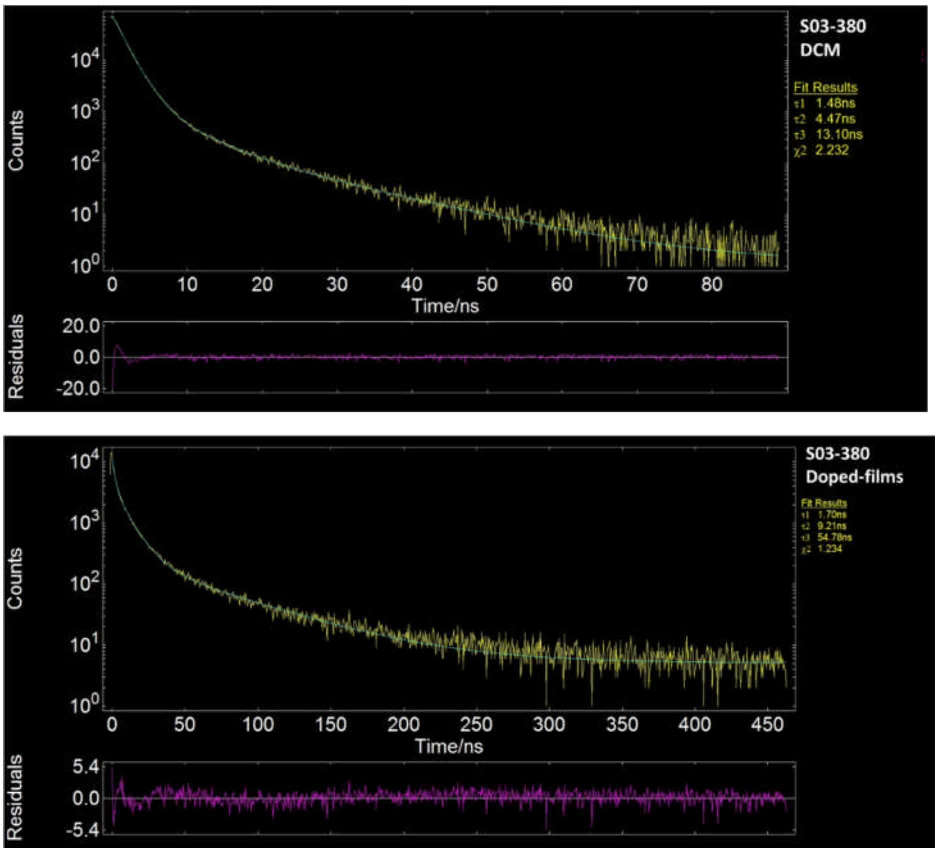

Fig. S102. Luminescence lifetime data for **C2** in DCM solution (upper) and doped PMMA-film (lower).

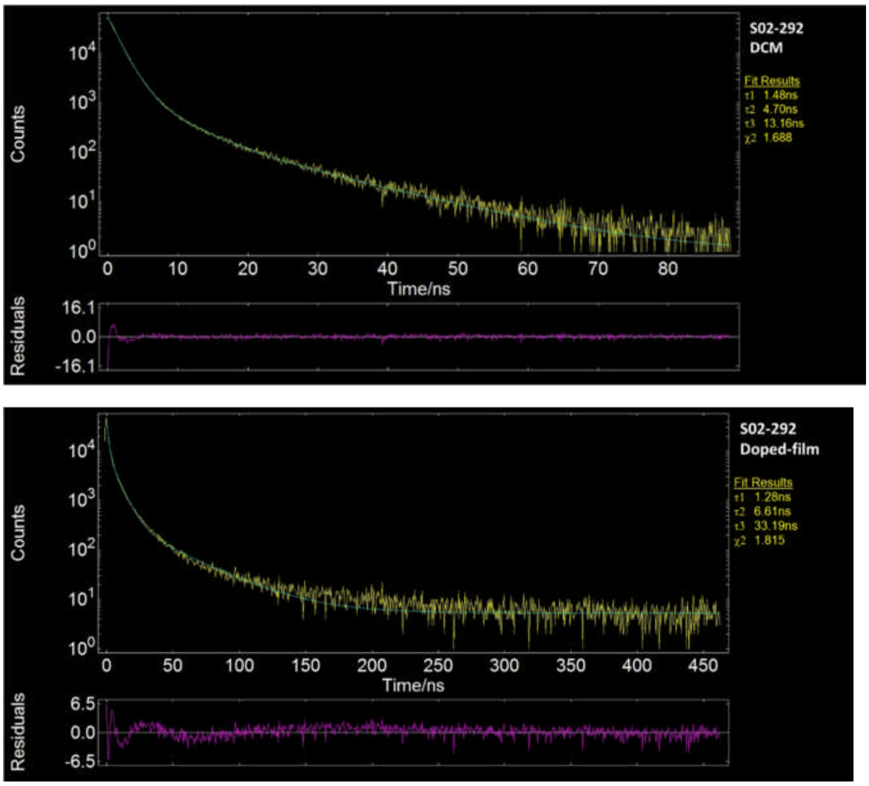

Fig. S103. Luminescence lifetime data for **C3** in DCM solution (upper) and doped PMMA-film (lower).

6. References

1. J. Canceill, J. Gabard and A. Collet, *J. Chem. Soc. Chem. Commun*. **1983**, 122-123.

2. D. Takamatsu, K.-i. Fukui, S. Aroua and Y. Yamakoshi, *Org. Biomol. Chem.* **2010**, **8**, 3655-3664.

3. H. Zhang, R. Hao, J. K. Jackson, M. Chiao and H. Yu, *Chem. Comm.* **2014**, 50, 14843-14846.

4. M. M. Naoum, A. A. Fahmi, A. A. Refaie and M. A. Alaasar, *Liq. Cryst.* **2012**, 39, 47-61.

5. O. Taratula, P. A. Hill, Y. Bai, N. S. Khan and I. J. Dmochowski, *Org. Lett.* **2011**, 13, 1414-1417.

6. K. A. McGee, K. R. Mann, *Inorg. Chem.* **2007**, *46*, 7800-7809.

7. C. Li, Y. Liu, Y. Wu, Y. Sun and F. Li, *Biomaterials*, **2013,** 34, 1223-1234.

8. C.-C. Wang, Y.-M. Jing, T.-Y. Li, Q.-L. Xu, S. Zhang, W.-N. Li, Y.-X. Zheng, J.-L. Zuo, X.-Z. You and X.-Q. Wang, *Eur. J. Inorg. Chem.* **2013**, 5683-5693.

9. G. M. Sheldrick, *Acta Crystallogr. A* **2008**, *A64*, 112-122.

10. P. Van der Sluis and A. L. Spek, *Acta Crystallogr. A* **1990**, *A46*, 194-201.

11. G. A. Crosby, J. N. Demas, *J. Phys. Chem.*, **1971**, *75*, 991-1024.

12. W. H. Melhuish, *J. Phys. Chem.*, **1961**, *65*, 229-235.

13. A. M. Brouwer, *Pure Appl. Chem.*, **2011**, *83*, 2213-2228.

14. N. C. Greenham, I. D. W. Samuel, G. R. Hayes, R. T. Phillips, Y. A. R. R. Kessener, S. C. Moratti, A. B. Holmes and R. H. Friend, *Chem. Phys. Lett*., **1995**, *241*, 89–96.
